# Supplementary material for: TWINGEN: protocol for an observational clinical biobank recall and biomarker cohort study to identify Finnish individuals with high risk of Alzheimer’s disease
Source: BMJ Open. 2024 Jun 12;14(6):e081947. doi: 10.1136/bmjopen-2023-081947 (PMC11177688; doi:10.1136/bmjopen-2023-081947)
Supplement: Supplementary data [file bmjopen-2023-081947supp003.pdf]

BMJ Publishing Group Limited (BMJ) disclaims all liability and responsibility arising from any reliance placed on this supplemental material which has been supplied by the author(s)

Supplementary Table 1. Previous data from the EH-Epi subcohort

Omics	n pairs (singletons)	Platform	Tissue	Study reference
Genotype	199 (4)	Illumina HumanCoreExome BeadChip	WBC	Drouard et al., 2022
DNA methylation	199 (4)	Illumina HumanMethylation450 BeadChip	WBC	Huang et al., 2020
Transcriptomics	195 (4)	Illumina HumanHT-12 v4 Expression BeadChip	WBC	Huang et al., 2018
miRNA	189 (3)	Illumina HiSeq	WBC	
Metabolomics	212 (10)	Nuclear magnetic resonance spectroscopy	Plasma	Drouard et al., 2022
Proteomics	210 (6)	Olink Explore	Plasma	

WBC = white blood cells

Supplementary Table 2. Primary and secondary references for studies on the older Finnish Twin Cohort study data

	Primary reference (PMID)	Secondary references (PMIDs)
Education	27752535	37295953, 28975875, 23344794, 15368602, 11523706
Chronic or serious illness	31462340	3499347, 7629467, 9039240, 10208188, 15935024, 19210186, 19811382, 19952836, 20516052, 20680407, 21522028, 21612334, 30626223
Smoking	3335880	7198244, 7198250, 6984028, 2661052, 10857247, 12446254, 15053857, 15203778, 17174039, 17181913, 17549066, 19246432, 19423697, 19811391, 20418888, 20418890, 23752247, 24570102, 26359785, 26407342, 27134767, 29054888, 28399944, 29730550, 30001359, 31466478, 32157176, 32361366, 32938311, 35143046, 37322846
Alcohol use	26359785	27351919, 22215005, 20858964, 16222166, 12446254, 10857250, 1590545, 3335880, 3307505, 6984028, 7198250
Sleep	31462340	37322846, 37193395, 32907578, 29047168, 25451441, 23729932, 23509990, 21731146, 20883457, 19015202, 18275555, 17969458, 16230426, 14573377, 11525419, 11403523, 8642234
Medications	29311254	12446254, 27136415, 27723547
Anthropometrics (weight, height)	26979986	10702760, 10754982, 11523706, 11665321, 12953173, 14624724, 15368602, 15971946, 16484449, 18239571, 23298777, 27964777, 31364586, 31761633, 35534559
Blood pressure	33154181	6982602, 6540964, 2858745, 3101779, 3630648, 2661052, 1418922, 10781651, 12006398, 14624727, 16268117,17129598, 19952836, 20229313, 20680407, 29311254, 32520614, 35259029
Cholesterol	33154181	2661052, 9630073, 17940543, 22286219, 23031429, 23258601, 23532744, 24082313
Diabetes	33154181	1418922, 1473616, 10768089, 10753052, 14514580, 15105411, 15935024, 19952836, 20401462, 20680407, 20706830, 23124702, 26678054, 26847990, 36638183
Life satisfaction	11092440	22980535, 22215005, 19015202, 15656900, 15583908, 14982128, 12059849, 11576076, 11229985, 11092440, 9806309, 6540964, 6540959
Loneliness	11092440	See life-satisfaction papers, loneliness is one item in the LS scale
Cognition (TELE/TICS-m)	30315123	11893836, 14512720, 14707319, 16222166, 18025784, 19587088, 20858964, 21619517, 21700581, 23532744, 24082313, 26370660, 27752535, 27589524, 30090846, 30315123, 31477183, 32843700, 32954285, 33154181, 34607247, 35134847
Depressive symptoms	Preprint: 10.31234/osf.io/jvmtz	33629475, 32361366, 31469194, 28202098, 27723547, 27161145, 27055784, 25451441, 21330272, 17181913, 15607014, 15583908, 12558538, 10893486,
Personality (EPI)	3346805	8189349, 9806309, 11576076, 12115554, 19811701, 20639285, 24570102, 24828478, 26362575, 27507146, 6540964, 3335880, 23117535, 25993607, 27089181, 33629475

Accelerometer (physical activity)	30445494	35770444, 32259351, 30626223, 30445494, 30090846, 29925959
Multi-omics data	35259029	Also multiple metabolomics, transcriptomics and methylation (EWAS and epigenetic ageing) analyses
DNA	31462340	Multiple GWAS consortia papers, including PMIDs: 17549066, 22241830, 23752247, 26131930, 26407342, 27089181, 27134767, 27225129, 27911795, 28892072, 30038396, 30482948, 30643251, 31294817, 32157176, 36653562

EPI = Eysenck Personality Inventory; EWAS = epigenome-wide association study; GWAS = genome-wide association study; LS = Life Satisfaction; TELE = telephone assessment for dementia; TICS-m = modified Telephone Interview for Cognitive Status
