# Supplementary figures and images for: TWINGEN: protocol for an observational clinical biobank recall and biomarker cohort study to identify Finnish individuals with high risk of Alzheimer’s disease
Source: BMJ Open. 2024 Jun 12;14(6):e081947. doi: 10.1136/bmjopen-2023-081947 (PMC11177688; doi:10.1136/bmjopen-2023-081947)

Supplementary Figure 1. Blood samples collected in TWINGEN

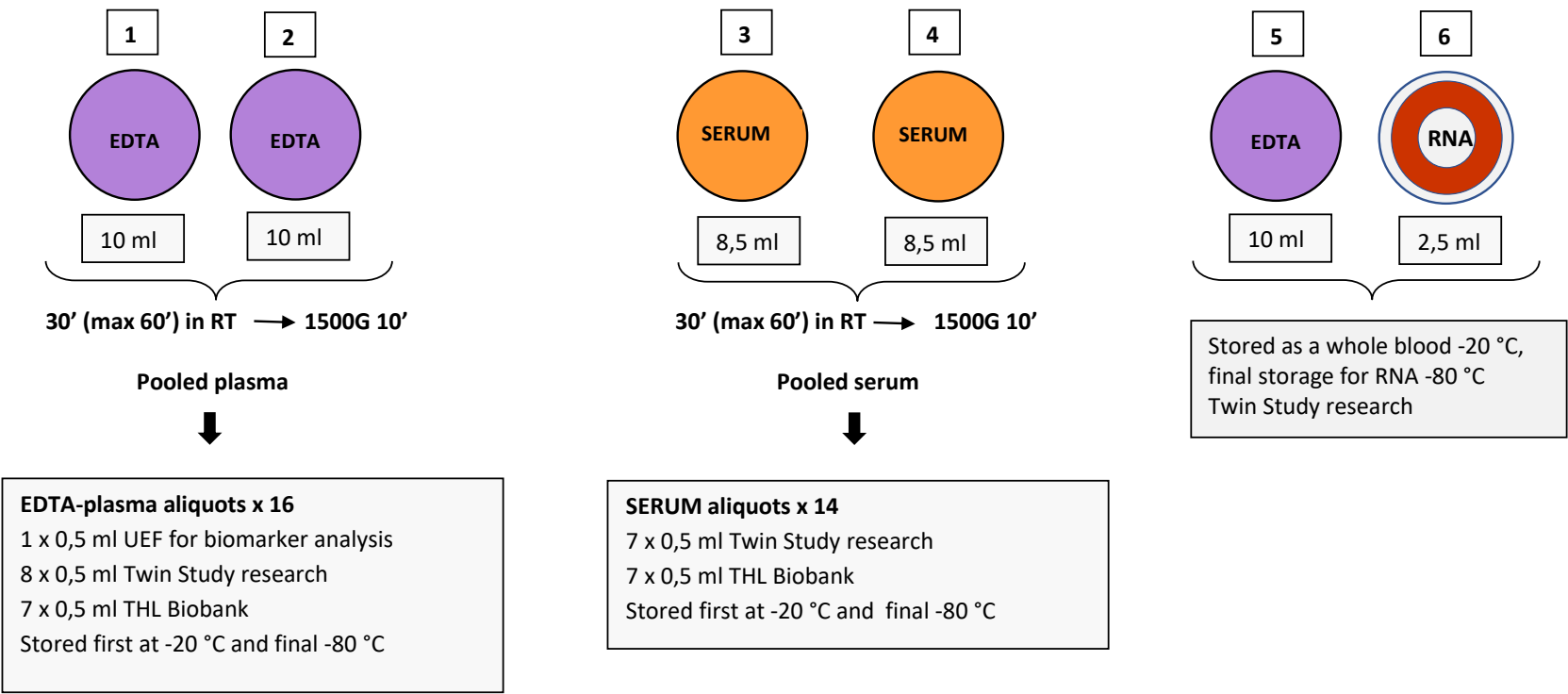

Supplement: Supplementary data [file bmjopen-2023-081947supp001.pdf]

**Supplementary Figure 2. Flow-chart of data and planned sub-studies within TWINGEN**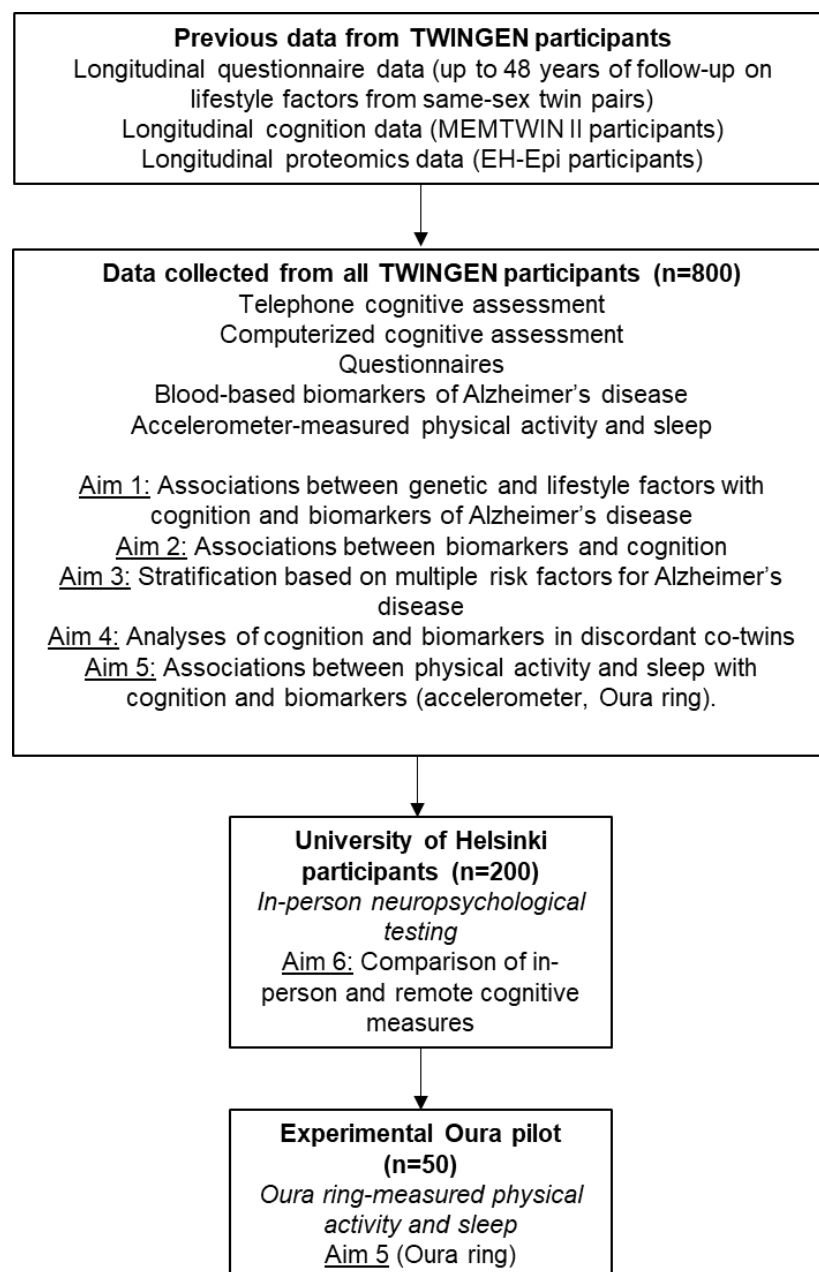

Supplement: Supplementary data [file bmjopen-2023-081947supp002.pdf]
