# Supplementary material for: TWINGEN: protocol for an observational clinical biobank recall and biomarker cohort study to identify Finnish individuals with high risk of Alzheimer’s disease
Source: BMJ Open. 2024 Jun 12;14(6):e081947. doi: 10.1136/bmjopen-2023-081947 (PMC11177688; doi:10.1136/bmjopen-2023-081947)
Supplement: Author's manuscript [file bmjopen-2023-081947.draft_revisions.pdf]

BMJ Open is committed to open peer review. As part of this commitment we make the peer review history of every article we publish publicly available.

When an article is published we post the peer reviewers' comments and the authors' responses online. We also post the versions of the paper that were used during peer review. These are the versions that the peer review comments apply to.

The versions of the paper that follow are the versions that were submitted during the peer review process. They are not the versions of record or the final published versions. They should not be cited or distributed as the published version of this manuscript.

BMJ Open is an open access journal and the full, final, typeset and author-corrected version of record of the manuscript is available on our site with no access controls, subscription charges or pay-per-view fees (http://bmjopen.bmj.com).

If you have any questions on BMJ Open's open peer review process please email info.bmjopen@bmj.com

BMJ Open

TWINGEN – protocol for an observational clinical biobank recall and biomarker study to identify individuals with high risk of Alzheimer's disease

Journal:	*BMJ Open*
Manuscript ID	bmjopen-2023-081947
Article Type:	Protocol
Date Submitted by the Author:	10-Nov-2023
Complete List of Authors:	Vuoksimaa, Eero; University of Helsinki Institute for Molecular Medicine Finland<br>Saari, Toni; University of Helsinki Institute for Molecular Medicine Finland<br>Aaltonen, Aino; University of Helsinki Institute for Molecular Medicine Finland<br>Aaltonen, Sari; University of Helsinki Institute for Molecular Medicine Finland<br>Herukka, Sanna-Kaisa; University of Eastern Finland Institute of Clinical Medicine, Neurology; Kuopio University Hospital, Department of Neurology, NeuroCenter<br>Iso-Markku, Paula; University of Helsinki Institute for Molecular Medicine Finland<br>Kokkola, Tarja; University of Eastern Finland Institute of Clinical Medicine, Neurology<br>Kyttälä, Aija; Finnish Institute for Health and Welfare<br>Kärkkäinen, Sari; University of Eastern Finland Institute of Clinical Medicine, Neurology<br>Liedes, Hilkka; University of Helsinki Institute for Molecular Medicine Finland; VTT Technical Research Centre of Finland Ltd<br>Ollikainen, Miina; University of Helsinki Institute for Molecular Medicine Finland<br>Palviainen, Teemu; University of Helsinki Institute for Molecular Medicine Finland<br>Ruotsalainen, Ilona; University of Helsinki Institute for Molecular Medicine Finland; VTT Technical Research Centre of Finland Ltd<br>Toivola, Auli; Finnish Institute for Health and Welfare<br>Urjansson, Mia; University of Helsinki Institute for Molecular Medicine Finland<br>Vasankari, Tommi ; UKK Institute; Tampere University Faculty of Medicine and Health Technology<br>Vähä-Ypyä, Henri; UKK Institute<br>Forsberg, Markus M.; University of Eastern Finland Faculty of Health Sciences, School of Pharmacy; VTT Technical Research Centre of Finland Ltd<br>Hiltunen, Mikko; University of Eastern Finland Institute of Clinical Medicine, Neurology<br>Jalanko, Anu; University of Helsinki Institute for Molecular Medicine Finland<br>Kälviäinen, Reetta; University of Eastern Finland Institute of Clinical

Medicine, Neurology; Kuopio University Hospital, Department of Neurology, NeuroCenter
Kuopio, Teijo; Central Finland Health Care District, Central Finland Biobank
Lähteenmäki, Jaakko; VTT Technical Research Centre of Finland Ltd
Nyberg, Pia; Wellbeing Services County of North Ostrobothnia, Biobank Borealis of Northern Finland, Oulu University Hospital; University of Oulu, Translational Medicine Research Unit
Männikkö, Minna; University of Oulu, Arctic Biobank, Infrastructure for Population Studies, Faculty of Medicine
Serpi, Raisa; Wellbeing Services County of North Ostrobothnia, Biobank Borealis of Northern Finland, Oulu University Hospital
Siltanen, Sanna; Wellbeing Services County of Pirkanmaa, Finnish Clinical Biobank Tampere, Tampere University Hospital
FinnGen, -; -
Palotie, Aarno; University of Helsinki Institute for Molecular Medicine Finland; Broad Institute Stanley Center for Psychiatric Research
Kaprio, Jaakko; University of Helsinki Institute for Molecular Medicine Finland
Runz, Heiko; University of Helsinki Institute for Molecular Medicine Finland; Biogen Inc, Translational Sciences
Julkunen, Valtteri; University of Eastern Finland Institute of Clinical Medicine, Department of Neurology; Kuopio University Hospital, Department of Neurology, NeuroCenter

Keywords: Dementia, GENETICS, Observational Study

SCHOLARONE™
Manuscripts

TWINGEN – protocol for an observational clinical biobank recall and biomarker study to identify individuals with high risk of Alzheimer's disease

Eero Vuoksimaa[1]*, Toni T. Saari[1]*, Aino Aaltonen[1], Sari Aaltonen[1], Sanna-Kaisa Herukka[2,3], Paula Iso-Markku[1], Tarja Kokkola[2], Aija Kyttälä[4], Sari Kärkkäinen[2], Hilkka Liedes[1,5], Miina Ollikainen[1], Teemu Palviainen[1], Ilona Ruotsalainen[1,6], Auli Toivola[4], Mia Urjansson[1], Tommi Vasankari[7,8], Henri Vähä-Ypyä[7], Markus M. Forsberg[9,10], Mikko Hiltunen[2], Anu Jalanko[1], Reetta Kälviäinen[2,3], Teijo Kuopio[11], Jaakko Lähteenmäki[6], Pia Nyberg[12,13], Minna Männikkö[14], Raisa Serpi[12], Sanna Siltanen[15], FinnGen[†], Aarno Palotie[1,16,17], Jaakko Kaprio[1], Heiko Runz[1,18] & Valtteri Julkunen[2,3]

*Contributed equally, corresponding authors

[†]A list of authors and their affiliations appears at the end of the paper

[1]*Institute for Molecular Medicine Finland (FIMM), HiLIFE, University of Helsinki, Helsinki, Finland*

[2]*Department of Neurology, Institute of Clinical Medicine, University of Eastern Finland, Kuopio, Finland*

[3]*Department of Neurology, Neurocenter, Kuopio University Hospital, Kuopio, Finland*

[4]*Finnish Institute for Health and Welfare, Helsinki, Finland*

[5]*VTT Technical Research Centre of Finland Ltd., Oulu, Finland*

[6]*VTT Technical Research Centre of Finland Ltd., Espoo, Finland*

[7]*UKK Institute for Health Promotion Research, Tampere, Finland*

[8]*Faculty of Medicine and Health Technology, Tampere University, Tampere, Finland*

[9]*School of Pharmacy, Faculty of Health Sciences, University of Eastern Finland, Kuopio, Finland*

[10]*VTT Technical Research Centre of Finland Ltd., Kuopio, Finland*

[11]*Central Finland Biobank, Central Finland Health Care District, Jyväskylä, Finland*

[12]*Biobank Borealis of Northern Finland, Oulu University Hospital, Wellbeing Services county of North Ostrobothnia, Oulu, Finland*

[13]*Translational Medicine Research Unit, University of Oulu, Oulu, Finland*

[14]*Arctic Biobank, Infrastructure for Population Studies, Faculty of Medicine, University of Oulu, Oulu, Finland*

For peer review only - http://bmjopen.bmj.com/site/about/guidelines.xhtml

[15]*Finnish Clinical Biobank Tampere, Tampere University Hospital, Wellbeing Services County of Pirkanmaa, Tampere, Finland*

[16]*Analytic and Translational Genetics Unit, Department of Medicine, Department of Neurology and Department of Psychiatry, Massachusetts General Hospital, Boston, MA, USA.*

[17]*The Stanley Center for Psychiatric Research and Program in Medical and Population Genetics, The Broad Institute of MIT and Harvard, Cambridge, MA, USA*

[18]*Translational Sciences, Biogen, Cambridge, MA, USA*

For peer review only

Abstract

Introduction: A better understanding of the earliest stages of Alzheimer's disease (AD) could expedite the development or administration of treatments. Large population biobanks hold the promise to identify individuals at an elevated risk of AD and related dementias based on health registry information. Here, we establish the protocol for an observational clinical recall and biomarker study called TWINGEN with the aim to identify individuals at high risk of AD by assessing cognition, health and AD-related biomarkers. Suitable candidates were identified and invited to participate in the new study among Finnish biobank donors according to TWINGEN study criteria.

Methods and analysis: A multi-center study (n=800) to obtain blood-based biomarkers, telephone-administered and web-based memory and cognitive parameters, questionnaire information on lifestyle, health and psychological factors, and accelerometer data for measures of physical activity, sedentary behavior and sleep. A sub-cohort are being asked to participate in an in-person neuropsychological assessment (n=200) and wear an Oura ring (n=50). All participants in the TWINGEN study have genome-wide genotyping data and up to 48 years of follow-up data from the population-based older Finnish Twin Cohort (FTC) study of the University of Helsinki. TWINGEN data will be transferred to Finnish Institute of Health and Welfare (THL) biobank and we aim to further to transfer it to the FinnGen study where it will be combined with health registry data for prediction of AD.

Ethics and dissemination: This recall study consists of FTC/THL/FinnGen participants whose data were acquired in accordance with the Finnish Biobank Act. The recruitment protocols followed the biobank protocols approved by Finnish Medicines Agency. The TWINGEN study plan was approved by the Ethics Committee of Hospital District of Helsinki and Uusimaa (number 16831/2022). THL Biobank approved the research plan with the permission no: THLBB2022_83.

For peer review only

Keywords: Alzheimer's disease, biobank, recall study, cognition, biomarkers

Article summary

Strengths and limitations of this study

- A large sample of individuals is recruited from a representative biobank database
- Using health registry information, we exclude those with documented AD or other neurological or psychiatric diseases that can affect cognition. Pre-screening limits the sending of unnecessary invitations and saves costs
- Participants have up to 48 years of follow-up questionnaire and clinical data from the Finnish Twin Cohort study and these data can be combined with multifaceted Finnish health registry information. Previous genotype data is available in the biobank from all TWINGEN study participants.
- We assess the feasibility of remote cognitive testing and blood samples in large-scale screening of AD risk, translating to the requirements of intervention trials and clinical practice
- Limitations of the study are a lack of gold standard biomarkers (cerebrospinal fluid, positron emission tomography imaging) and neurological examinations

Word count: 3996

For peer review only

Introduction

Alzheimer's disease (AD) – the most common cause of dementia – is characterized by pathological accumulation of beta-amyloid (Aβ) and tau in the brain.[1] As populations age, the prevalence of dementia is projected to nearly double every two decades[1,2] and AD and related dementias are becoming one of the most common causes of death in many countries (20% of deaths in Finland ranking it as the third most common cause of death).[3] The AD disease process starts up to 20-30 years before the diagnosis, so intervention trials targeted at preclinical or prodromal stages of AD are of high priority, but time-consuming and costly with screen-failure rates of 78-88%.[4]

In clinical practice, AD is diagnosed mainly based on the clinical phenotype, episodic memory impairment being the cognitive hallmark, while in research there has been a shift from clinical diagnosis to biological classification independent of the cognitive status.[5] Evidence of diagnostic properties of different blood-based biomarkers is rapidly accumulating,[6] but population-based studies are still scarce. In one population-based study, 11% of older adults (median age 74 years) without dementia were found to have Aβ pathology using blood-based biomarkers.[7]

Our earlier pilot study explored the feasibility of FinnGen, a nationwide Finnish biobank study[8] in recruiting individuals with AD to an observational study via the biobank.[9] Our protocol included blood-based biomarkers and remote cognitive assessment, approaches that are suggested to improve the recruitment of participants for AD trials.[10–12] Another FinnGen study showed that biobank participants could be recontacted for additional data collection in a larger scale as well.[13] The full potential of population biobank datasets lies in the large cohorts of undiagnosed individuals who are at the risk of developing AD in near future and might be suitable for targeted screening for early diagnostics and interventional trials encompassing both pharmacological treatments and lifestyle interventions.

For peer review only - http://bmjopen.bmj.com/site/about/guidelines.xhtml

After showing in our pilot study that recall of biobank participants with AD to a clinical study including multimodal (remote and in-person) cognitive assessment and blood-based biomarker analyses is feasible,[9] we modified the protocol of the pilot study to target cognitively unimpaired older adults (based on health registry data) in the current study. Additionally, we augmented the assessment battery with passive technology for measuring physical activity, sedentary behavior, and sleep.

This protocol paper describes TWINGEN, a population-based follow-up study investigating the utility of easily implementable methods for assessing the risk of AD. We aim to conduct a proof-of-principle study for using biobank registries as a platform for recruiting participants suitable for clinical trials, particularly in diseases where recruitment and screening has generally been challenging. We also focus on the remote cognitive assessment methods and blood-based biomarkers of AD. The study also aims to enrich existing biobank data derived from a long-standing prospective twin study with cognitive and lifestyle measures. The research setting is unique as it utilizes the pre-screening and recall option based on the data of the biobank in combination with long preceding population-based follow-up data from the twin study.

Methods and analysis

Study participant selection

The target group of the TWINGEN study are individuals who have participated in the older Finnish Twin Cohort (FTC) study of the University of Helsinki (UH), and whose samples and data have been transferred to the Finnish Institute of Health and Welfare (THL) biobank in 2018. The main selection criteria in the biobank were previous participation in FTC, age (66-85), place of current residence in Finland, Finnish as the first language, and no known diagnosis affecting cognition in biobank records. Selection also included participation in the FinnGen biobank study.8 This criterion

For peer review only

was important so that the TWINGEN data to be returned to THL Biobank can later be requested from the biobank for the FinnGen study and combined with its extensive gene and health register data. Below, we describe each of the data sources and the study protocol.

The older FTC study

The older FTC study from the University of Helsinki is a population-based study that includes all Finnish same-sex twins born before 1958 and living in Finland at the start of the study in 1974 (Figure 1).[14] The baseline survey was conducted in 1975 via postal questionnaire and 27750 individuals participated with an 89% participation rate. Follow-up questionnaires were sent in 1981 (n=24684 with a 84% response rate), 1990 for those born in 1930 or later (n=12502; 77%), and in 2011–2012 for those born in 1945–1957 (n=8410; 72%).[15] Those born in 1938–1944 have also participated in MEMTWIN II study (n=1772) that utilized telephone interview to assess cognition.[16] Some of the twins born in 1945–1957 have participated in Essential Hypertension EPIgenetics study (EH-EPI, n=445).[17] The MEMTWIN II and EH-EPI study participants are the primary groups of interest in TWINGEN because they have either earlier cognitive (MEMTWIN II; Table 1) or multi-omics data (EH-EPI; Supplementary Table 1) available.

For peer review only - http://bmjopen.bmj.com/site/about/guidelines.xhtml

Table 1. Cognitive measures used in the TWINGEN study

In-person neuropsychological assessment	Telephone (TELE/TICS-m3)	Web-based computerized assessment (cCOG)
**Memory and learning**		
CERAD Word List Learning	Word List Learning	Episodic Memory Learning
CERAD Word List Recall	Word List Recall	Episodic Memory Recall
CERAD Word List Recognition		Episodic Memory Recognition
CERAD Constructional Praxis Recall		
WMS-III Logical Memory Story A		
WMS-III Logical Memory Story A Recall		
**Executive function**		
Trail Making Test-B		Modified Trail Making Test-B
Stroop Interference		
Stroop Set-Shifting		
**Visuospatial skills & visuoconstruction**		
CERAD Constructional Praxis		Fragmented Letters
CERAD Clock Drawing Test		
**Language skills & fluency**		
CERAD Naming Test	Similarities	
CERAD Semantic Fluency	Semantic Fluency	
**Processing speed**		
Trail Making Test-A		Modified Trail Making Test-A
Stroop Word Reading		
Stroop Color Naming		
**Global cognition**		
CERAD MMSE	TELE Global Score	cCOG Global Score
	TICS Global Score	
	TICS-m3 Global Score	

MEMTWIN-II participants have prior TELE/TICS-m data. CERAD-nb = Consortium to Establish a Registry for Alzheimer's Disease – neuropsychological battery; MMSE = Mini-Mental State Examination; TELE = telephone assessment for dementia; TICS = Telephone Interview for Cognitive Status; TICS-m = modified Telephone Interview for Cognitive Status; TICS-m3 = TICS-m including three learning trials in the Word List Learning; WMS-III = Wechsler Memory Scale 3rd edition

For peer review only

To achieve the target number of 800 participants, the biobank selection was expanded to the twins born in 1945–1952 who had not participated in MEMTWIN II or EH-EPI. Most of the invited twins were from same-sex pairs, but we also invited twins from opposite-sex pairs included in the older FTC study in year 1995–96, when they replied to a brief health questionnaire (Figure 1). We prioritized invitations to participants who lived closest to one of the six study sites. All TWINGEN participants from same-sex twin pairs have longitudinal questionnaire data on health and health-related behaviors from years 1975, 1981 and 1990, and those born in 1945–1957 have data also from year 2011–2012. An overview of longitudinal data available for the MEMTWIN II and EH-EPI and other TWINGEN participants are presented in Table 2. Necessary inclusion criteria for TWINGEN were available DNA sample in THL Biobank and not having any of the exclusion criteria (Table 3). Additional references for studies using the older FTC data are found in Supplementary Table 2.

For peer review only

For peer review only - http://bmjopen.bmj.com/site/about/guidelines.xhtml

Table 2. Previous data from the participants recruited in TWINGEN

	Baseline assessment (1975)	First follow-up (1981)	Second follow-up (1990)	Third follow-up (2011-2012)	MEMTWIN-II (2013-2017)*	EH-Epi (2012-2015)	Primary study reference
Education	x	x					Vuoksimaa et al., 2016b
Chronic or serious illness	x	x	x	x	x	x	Kaprio et al., 2019
Physical activity	x	x	x			x	Piirtola et al., 2017
Smoking	x	x	x	x		x	Kaprio & Koskenvuo 1988
Alcohol use	x	x	x	x		x	Sipilä et al., 2016
Sleep	x	x	x	x			Kaprio et al., 2019
Medications	x	x	x	x	x	x	Huang et al., 2018
Anthropometrics (weight, height)	x	x	x	x		x	Piirtola et al., 2017
Dietary habits		x		x		x	Kaprio et al., 2019
Blood pressure		x	x	x		x	Iso-Markku et al., 2021
Diabetes	x	x	x	x		x	Iso-Markku et al., 2021
Subjective memory complaints					x		
Life satisfaction	x	x	x	x			Koivumaa-Honkanen et al., 2000
Loneliness	x	x	x	x			Koivumaa-Honkanen et al., 2000
Social support			x	x			Romanov et al., 2003
Cognition (TELE/TICS-m)					x		Lindgren et al., 2019
Depressive symptoms			BDI	CES-D	CES-D		Saari et al., 2023
Personality (EPI)	x	x		x			Rose et al., 1988
Accelerometer (physical activity)					x		Waller et al., 2019
Multi-omics data						x	Drouard et al., 2022
DNA					x	x	Kaprio et al., 2019

For peer review only

BDI = Beck Depression Inventory; CES-D = Center for Epidemiological Studies-Depression scale; EPI = Eysenck Personality Inventory; LS = Life Satisfaction; TELE = telephone assessment for dementia; TICS-m = modified Telephone Interview for Cognitive Status. See Supplementary Table 2 for additional references on previous studies using older Finnish Twin Cohort study data. *Third follow-up assessments for those born in 1938–1944.

For peer review only

For peer review only - http://bmjopen.bmj.com/site/about/guidelines.xhtml

Table 3. Exclusion criteria for the TWINGEN study

ICD-10 code	Explanation
G30 and F00	Any variant of Alzheimer's disease or any dementia relating to Alzheimer's disease, dementia with Lewy bodes, frontotemporal dementia and mixed dementia
F01-F03	Dementia, any etiology
G20	Parkinson's disease
G35	Multiple sclerosis
I60, I61 and I63	Intracerebral hemorrhage, subarachnoid hemorrhage or ischemic stroke and their subcategories
S06.1-S06.7	Traumatic brain injuries other than concussion (S06.0)
F20	Schizophrenia
F31	Bipolar disorder
F33, F32.1-F32.3, F34	Recurrent depression, moderate or severe depression and long-lasting mood disorders
F60	Personality disorders
F10	All diagnoses relating to excessive usage of alcohol
F11-F19	Intoxication because of opioids, cannabinoids, sedative medication, cocaine or hallucinogens
F70-73	Different stages of intellectual disabilities

For peer review only

THL Biobank and the FinnGen study

DNA samples of FTC participants were transferred to the FinnGen project, a nationwide biobank research study including sample donors with DNA samples from Finnish biobanks. In TWINGEN, seven biobanks were included: THL Biobank, Arctic Biobank, Biobank Borealis, Biobank of Eastern Finland, Central Finland Biobank, Finnish Clinical Biobank Tampere and Helsinki Biobank. FinnGen has combined all biobank samples and linked these to registry data including e.g., medication history, hospital discharge, and mortality.[8] The biobank sample donors included in FinnGen provide a good representation of the Finnish population (risteys.finregistry.fi).

Recall procedure

The results of a THL Biobank feasibility assessment indicated that there were 6053 individuals aged 65–85 who have participated in the FTC of UH and have data in the THL Biobank. Approximately 1000 individuals were excluded due to our exclusion criteria of Alzheimer's disease and other neurological or psychiatric diseases that can affect cognition (Figure 1; Table 3). Furthermore, the genotype data of approximately 100 individuals have been verified by FinnGen, but these data have not been returned to their respective biobanks at the time of the feasibility assessment; thus, these individuals are excluded from the pool of potentially eligible participants. The target sample size of 800 participants was chosen as it fits the timeframe and resources of the study and is sufficiently large for stratifying participants at varying risks for AD.[7]

As a biobank recall study, TWINGEN participants are contacted by the THL Biobank by an invitation letter. The invitation letter includes information about the individuals' prior participation to the older FTC study and the transfer to and storage of samples and data at the THL Biobank, information about the participation in the FinnGen study via the biobank, and information about the new TWINGEN study. The invitation letter also contains two separate consent forms: one for the participation in the TWINGEN study and the biobank consent for THL Biobank. The consent for

For peer review only - http://bmjopen.bmj.com/site/about/guidelines.xhtml

THL Biobank is needed to store the new samples and data obtained in TWINGEN to THL Biobank and to confirm the biobank participation with a written biobank consent, which is the primary basis for storing samples and data into a biobank. After receiving consent forms, UH research staff contacts the potential participants to verify that the individuals have understood the purpose and procedures of the study. Additionally, as the health registry data available in the biobank is not up to date but reflects status at the end of year 2022, research staff verifies (via telephone) that the exclusion criteria are not met. Eligibility of each individual is assessed independently of their co-twin's vital status or eligibility.

Data collection

Data collection started on March 29th 2023 and is planned to be completed by the end of 2023. Data collection is conducted in six locations across Finland: at the Institute for Molecular Medicine Finland (FIMM), University of Helsinki in Helsinki, biobanks in four locations across Finland: in Jyväskylä (Central Finland biobank), Kuopio (Biobank of Eastern Finland), Oulu (Arctic Biobank and Biobank Borealis of Northern Finland) and Tampere (Finnish Clinical Biobank Tampere) and at the clinical laboratory of Turku University of Applied Sciences. Protocol for all participants includes telephone- and computer-administered cognitive testing, blood-draw and self-report questionnaire. Additionally, a waist-worn accelerometer will be given to participants who are willing to complete one week at-home measurement. Furthermore, individuals living in greater Helsinki area are invited to participate in in-person neuropsychological testing (target sample size n=200) and measurement of weight, height, waist circumference and blood pressure. In Helsinki, we also provide Oura rings for 50 participants who are willing and able to wear the ring and use the associated mobile app. After participating in the study, participants receive a report based on their performance in telephone interview-based and computerized tests of cognition and about their physical activity. A detailed description of the data collections is provided below.

For peer review only - http://bmjopen.bmj.com/site/about/guidelines.xhtml

Telephone interview for assessing cognitive status and function

Two validated telephone-administered cognitive screening instruments are used: a telephone assessment for dementia (TELE)[18] and the modified Telephone Interview for Cognitive Status (TICS-m).[19] TELE and TICS have been translated to Finnish and adapted to Finnish culture[20] and both have been used in the older FTC study. TICS-m, a modified version of TICS includes an additional delayed free recall of the 10-word list and has been used in the MEMTWIN II sub-study.[21] We further modified the TICS-m by including three learning trials of the 10-word list and this instrument is later referred to as TICS-m3.[9] We also included semantic fluency. Three trial word list task yields immediate and delayed free recall measures of episodic memory. Semantic fluency score is the number of animals named in 1 minute (Table 1). In the telephone interview, the participants are also asked about their functional abilities regarding household maintenance, ambulation, shopping, dressing and undressing, use of mobility aids, memory problems and possible visits to the doctor regarding memory problems. Telephone interviews were conducted by trained study nurses or psychologists.

Computerized web-based cognitive testing

Web-based cCOG tool created by Combinostics is used for computerized cognitive testing.[22] It includes six subtests: Episodic Memory (learning & recall), Reaction Time, Modified Trail Making A and B and Distorted Letters. The tasks measure visual processing, memory, processing speed, attention, and executive function (Table 1). In addition to cognitive tests, cCOG includes background questions (education) and a seven-item questionnaire designed to assess probable dementia with Lewy bodies.[23] The test battery takes about 25 minutes to complete and is performed via a keyboard and a mouse or a touchscreen device.

For peer review only - http://bmjopen.bmj.com/site/about/guidelines.xhtml

In-person neuropsychological tests

The participants in Helsinki study site undergo an in-person neuropsychological assessment, with a target sample size of 200 individuals. The minimum sample size to detect medium correlations (r = .3) between cognitive measures with a significance level = .05 and power = .8 is n = 85, thus, the target sample size of 200 individuals with in-person cognitive assessment is adequate for examining correlations between in-person and remote cognitive measures. The larger sample size is expected to be adequate for factor analyses of the neuropsychological battery.

The in-person neuropsychological assessment consists of the Consortium to Establish a Registry for Alzheimer's Disease neuropsychological battery (CERAD-nb)[24] and tests measuring executive functions, processing speed and episodic memory. The CERAD-nb includes Mini-Mental State Examination and Semantic Fluency, abbreviated Boston Naming Test, Word List Learning, Recall and Recognition, and Constructional Praxis (Copy and Recall). The Finnish version of the CERAD-nb also includes Clock Drawing test.[25] Finnish education adjusted cut-offs are available for total score and for each sub-test.[26] In addition to the CERAD-nb, we include the following tests: Logical Memory story A from the Wechsler Memory Scale 3rd edition,[27] Trail Making Test A and B,[28] and Stroop test.[29] The version of Stroop used in this study is the 40-item version used in the FINGER study[30] with an additional fourth “set-shifting” condition whereby the task is to name the color of ink (as in the classical Stroop condition) or to read out the color-word when the word is inside a rectangle. Neuropsychological tests are administered by trained psychologists. A summary of key cognitive measures of all three modalities are presented in Table 1.

Blood sample

A venous blood sample is drawn from the inside of the elbow or alternatively from the back of the hand. A total of six tubes are collected: three BD Vacutainer K2EDTA (10/10ml) tubes, two BD Vacutainer SSTII Advance-serum gel tubes (10/8.5ml), and one BD PAXgene Blood RNA (7/2.5ml)

For peer review only - http://bmjopen.bmj.com/site/about/guidelines.xhtml

tube (Supplementary Figure 1). Processing will be done immediately after the samples have been taken. Serum tubes are allowed to clot at least 30 minutes (max. 60') before separating. EDTA-plasma tubes do not clot but two EDTA tubes are also let sit for the same time as serum because of the easier workflow. Serum and two EDTA-plasma tubes are centrifuged (1500G) for 10' and the supernatants are pooled within serum and plasma. Samples are apportioned into 0.5 ml aliquots and stored at -20°C. One EDTA tube and PAXgene RNA-tube will be stored as a whole blood at -20°C. Serum, plasma and RNA samples final storage temperature is at -80°C. One of the 0.5 ml EDTA-plasma aliquot will be sent to University of Eastern Finland for biomarker analysis. RNA, EDTA whole blood samples, and half of the serum and plasma aliquots are dedicated to the Twin Study, while the other half will be available for research via THL Biobank.

Blood-based AD biomarkers

The primary blood-based biomarkers include phosphorylated-tau181 (p-tau181), phosphorylated-tau217 (p-tau217), Aβ1-42/40, glial fibrillary acidic protein (GFAP) and neurofilament light chain (NfL); all measured using Simoa HD-X analyzer (Quanterix, Billerica, Massachusetts, USA). Plasma p-tau181 levels are quantified using Simoa p-tau181 Advantage V2.1 Kit(Ref# 104111, Quanterix),[31] Aβ1-40, Aβ1-42, GFAP and NfL levels using Simoa Neurology 4-Plex E Advantage Kit (Ref# 103670, Quanterix),[32] and p-tau217 levels using ALZpath Simoa pTau-217 v2 Assay Kit (Ref# 104371, Quanterix).[33] Prior to analyses, EDTA plasma samples are thawed, mixed and centrifuged (10,000xg, 5 min, +20 °C). These biomarkers are determined at the Biomarker Laboratory of University of Eastern Finland.

Apolipoprotein E (APOE) genotype and polygenic risk scores

APOE status (ε4-carrier vs. non-carrier and number of ε4-alleles) is defined by two single-nucleotide polymorphisms, rs429358 and rs7412, in chromosome 19[34] and polygenic risk score (PRS; with and without APOE) of AD is based on the Bellenguez et al.[35] or newer meta-analysis if

For peer review only

For peer review only - http://bmjopen.bmj.com/site/about/guidelines.xhtml

available. Genetic data will be used to calculate also PRS's for diseases and traits (such as cardiovascular disease and educational attainment) that are related to risk and protective factors of dementia.[36]

Accelerometer-measured physical activity, sedentary behavior, and sleep

A tri-axial accelerometer (UKK RM42, UKK Terveyspalvelut Oy, Tampere, Finland) is used to monitor participants' daily physical activity, sedentary behavior, and sleep for seven consecutive days (Table 4).[37] In addition to total time spent in physical activity of different intensities and sedentary behavior, we will also measure number of bouts and length of the bouts of physical activity and sedentary behavior. The participants receive the devices during their in-person visit at FIMM or by mail if participating in other location. Participants are asked to wear the accelerometer on the hip during waking hours and on the wrist during sleep. At least a 4-day monitoring period with a minimum of 10 hours wear-time a day will be required for the adequate accelerometer data collection.[38]

The UKK RM42 device and its closely related counterpart Hookie AM 20 accelerometer have been used in samples with over 18,000 Finnish 18–85-year-old adults.[37,39] Thus, the UKK RM42 accelerometer is usable in the TWINGEN sample of 63–85-year-olds, and we will get an opportunity to compare the measurements against normative data of Finnish adults. The analyses of raw acceleration data of the UKK RM42 are based on validated algorithms; the technical details related to the recording and analyzing of raw acceleration data are given elsewhere.[40–42]

For peer review only - http://bmjopen.bmj.com/site/about/guidelines.xhtml

Table 4. Core UKK RM42 accelerometer and Oura ring parameters

The UKK RM42 accelerometer	Oura ring
Target n = 800	Target n = 50
Participants from all sites	Participants from Helsinki site
Wearing time = 1 week	Wearing time = 2 weeks
**Physical activity parameters**	
Light physical activity	Low intensity activity
Moderate physical activity	Medium intensity activity
Vigorous physical activity	High intensity activity
Total physical activity	Total physical activity
Number of steps	Number of steps
Standing time	
**Sedentary behavior parameters**	
Lying time	Inactive time
Reclining time	Resting time
Sitting time	
Number of breaks during sedentary time	
**Sleep parameters**	
Total sleep time	Total sleep time
Restless sleep time	Restless sleep percentage
Restful sleep time	Total amount of deep sleep
	Total amount or REM sleep
	Total amount of light sleep

Oura-measured physical activity, sedentary behavior and sleep

Oura ring (Gen3 Heritage, Ōura Health Ltd., Oulu, Finland) will be given to 50 participants in Helsinki study collection site. First, participants use a ring-size kit to determine optimal ring size and then they receive the ring either at their in-person visit or by mail. Participants are asked to wear the ring (width: 7.9mm, thickness: 2.55 mm, weight: 4–6g) in any finger for two weeks during

For peer review only

day and night, except when charging the ring every 4–6 days (20–80 minutes to fully charge). To monitor participant's sleep, sedentary behavior and physical activity (Table 4), the Oura ring uses infrared photoplethysmography sensors, negative temperature coefficient sensor and 3D accelerometer. Participants receive written instructions on using the Oura ring and downloading the Oura mobile application, through which they can access their own data. If necessary, the research staff provides phone guidance for both using the ring and installing the application. The data from Oura ring is transferred to participant's Oura application when opening the application and to a cloud server. Data collection will be monitored from Oura cloud server and participants' will be sent a reminder if there are no data from previous 2 days.

The Oura ring's sleep stage detection algorithm (wake, light non-rapid eye movement [NREM] sleep, deep NREM sleep, rapid eye movement [REM] sleep) has been validated against polysomnography and it showed 80–96% accuracy, 74–82% sensitivity and 79–98% specificity.[43] Furthermore, moderate-to-vigorous intensity physical activity and step count of Oura ring has shown strong correlations with accelerometer-measured corresponding values.[44]

Questionnaire

Participants are given a 16-page self-report questionnaire that includes many of the same measures as in previous questionnaires in years, 1975, 1981, 1990 and 2011 (Table 2). Questions cover anthropometrics, demographics, social relationships, chronotype, health (general, cardiovascular, dementia, memory, medications, vision, hearing, balance and mobility) and health-related behavior including sleep, physical activity, smoking and alcohol use. Psychological wellbeing scales included in the questionnaire are: 8-item Center for Epidemiologic Studies Depression;[45,46] 7-item Purpose in Life subscale from Ryff's Scales of Psychological Well-Being;[47,48] Extraversion (9 items) and Neuroticism (10 items) from the short version of Eysenck

For peer review only - http://bmjopen.bmj.com/site/about/guidelines.xhtml

Personality Inventory;[49,50] and four-item life satisfaction scale derived from questionnaires by Allardt.[51,52]

Patient and public involvement statement

None.

Aims, data analysis and future directions

In addition to the overarching aim of assessing the feasibility of biobank recall in the context of preclinical AD, we also have more focused research questions. By combining all data, we aim to stratify our participants in sub-groups of low, intermediate and high AD risk based on genetic, biomarker, cognitive, lifestyle and symptom data. This information is potentially useful for improving participant selection for AD drug and intervention trials. These methods would also be valuable in clinical settings where non-invasive and widely available tools for evaluating the presence of AD pathology underlying cognitive symptoms is important, especially once disease-modifying treatments become available. TWINGEN will also establish a baseline cohort that can be used in follow-up studies with neuroimaging and cerebrospinal fluid biomarkers. Through FinnGen, we will be able to follow these individuals based on their health records and thus will be ultimately able to predict progression to AD.

We aim to assess the comparability of in-person, telephone-based and computerized cognitive assessments using correlation analysis. We will also explore the distributions and correlations among blood-based biomarkers of AD-related pathologies and investigate the associations between cognition and biomarkers. Cognition will be treated both categorically (cognitive status) and as continuous outcomes for domain-specific measures (e.g., episodic memory, executive function). For the domain-specific cognitive composites, factor scores will be calculated similar to previous studies on preclinical AD and mild cognitive impairment.[53,54]

For peer review only

For peer review only - http://bmjopen.bmj.com/site/about/guidelines.xhtml

Additionally, the effect of genetic and lifestyle (with up to 48 years of follow-up) factors can be used in predicting cognitive and biomarker status. The measures of physical activity, sedentary behavior and sleep (Table 4) allow us to explore the relationships between physical activity and sleep with cognition and biomarkers. Additional parameters of physical activity and sleep are also available for detailed analyses. Although inclusion in the study was not dependent on the co-twin's participation, it is expected that full twin pairs will also participate. This will allow studying if the between-family associations are also evident in within-family comparisons and we can identify twin pairs who are discordant for cognition or biomarkers[55]

Ethics and dissemination

According to the Finnish Biobank Act, research collections, which have been collected, or whose collection started before the Biobank Act became into force on 1 September 2013, can be transferred to a biobank by a specific procedure, which includes an ethical evaluation and informing the sample donors either personally or by public announcement. Accordingly, biological samples of the FTC, including DNA and associated data were transferred to THL biobank in December 2018 to facilitate biobank research. The action of transferring FTC data to THL Biobank was publicly announced in major newspapers.

Recruitment protocols followed the biobank protocols approved by Fimea. The FinnGen study was approved by the Coordinating Ethics Committee of Hospital District of Helsinki and Uusimaa (HUS; statement number HUS/990/2017). The permit numbers of the decisions made by Finnish Institute for Health and Welfare and the Biobank Access Decisions for FinnGen samples and data utilized in FinnGen Data Freeze 9 are presented in Acknowledgements.

Before entering the TWINGEN study all potential study participants received detailed information regarding this study by a formal information letter. All study participants were asked for and

For peer review only

For peer review only - http://bmjopen.bmj.com/site/about/guidelines.xhtml

provided written informed consent. The recall study was reviewed and approved by the ethics committee of HUS (approval number 16831/2022). THL Biobank approved the research plan with the permission no: THLBB2022_83.

The data acquired in this study are managed and initially stored by UH. Keeping with the Biobank Act and the consent given by the participants, the data are also transferred to the THL Biobank for later research use. FinnGen request to use the TWINGEN data from THL Biobank, after which the data acquired in this study are linked with existing genetic and register data in a controlled FinnGen sandbox environment.

The data acquired in this study is subject to conditions of the IRB protocols and the policies of the Finnish biobank legislation and therefore unavailable for unsupervised usage. The data is stored in FIMM and THL Biobank, where approved researchers can access the data. Eventually the data will also be transferred to the secure FinnGen sandbox environment and linked to the registers available in FinnGen. The results of the study will be published in peer-reviewed journals and presented at scientific conferences.

Discussion

The TWINGEN study uses biobank registries for a recall study with the aim of identifying individuals at risk of AD. The collected data comprise many known risk factors[36] and scalable screening methods of AD. While these data are expected to yield novel insights even in isolation, the unique potential for discovery comes from combining the existing follow-up and newly collected data of TWINGEN participants with the existing data.

Since its inception, the FinnGen project has created new expansion areas with the aim of enriching phenotype information. The TWINGEN study addresses this goal by collecting cognitive, physical

For peer review only

activity, lifestyle and biological data that are returned to FinnGen via THL biobank, whereas FinnGen would allow for a registry-based follow-up of the TWINGEN study participants.

Author contributions

TTS: writing the original draft, revision and data collection. EV: writing the original draft, revision, resources, concept, design and supervision. AA: writing, revision and data collection. IR, HL, S-KH, SK, TK, SA: writing. MO, P-IM: revision. AK: design, administration and revision. AT: study coordination and revision. TV, HV-Y, MM, RS, PN, SS: resources and revision. V-MK, TK, EP, MR: resources. JK: concept, design, revision and resources. MU: study coordination, data collection. VJ: concept, design, revision, supervision. MMF, MH, JL, RK: consultation in the design of the study. HR: concept, design, revision. AP: concept, resources, revision. TP: data management. AJ: administration. TS and EV contributed equally to this paper.

Acknowledgements

We thank THL Biobank and biobank directors Eero Punkka (Helsinki Biobank) and Veli-Matti Kosma (Biobank of Eastern Finland) for providing resources to carry out this study. Biobank personnel who have collected the data and processed the samples: Sabrina Belgasem (Helsinki Biobank), Henna Palin, Minttu Virolainen and Anna-Kaisa Pohjonen (Finnish Clinical Biobank Tampere), Anu Outinen-Tuuponen, Marja-Leena Kytökangas, Riikka-Mari Siiro-Virtanen (Arctic Biobank and Biobank Borealis of Northern Finland), Senni Lipponen (Central Finland Biobank), Nina Hurula (Biobank of Eastern Finland) and Heidi Kalve, Anniina Friman and biomedical laboratory scientist students from the Turku University of Applied Sciences' clinical laboratory. The participants of the TWINGEN study were recruited through THL Biobank (study number THLBB2022_83). We thank Jyrki Tammerluoto and Steffi Besselink for legal services and Huei-Yi Shen for administrative work. We thank the participants of the Finnish Twin Cohort study for their participation to TWINGEN and all previous data collection waves.

For peer review only

Funding statement

TWINGEN study was funded by the FinnGen project with the aim to enrich the phenotype information in FinnGen to achieve the goals of the project. Finnish Twin Cohort study has been supported by the Academy of Finland (Grants 265240, 263278, 308248), the Sigrid Jusélius Foundation, NIH/NHLBI grant HL104125 and NIH Grant R01 AG060470. EV was supported by the Academy of Finland (grants 314639 and 345988). JK was supported by Centre of Excellence in Complex Disease Genetics (grants #312073 and #336823 from the Academy of Finland).

The FinnGen project is funded by Business Finland and AbbVie, AstraZeneca UK, Biogen, Bristol Myers Squibb (and Celgene Corporation & Celgene International II), Genentech, Merck Sharp & Dohme LLC, a subsidiary of Merck & Co., Inc., Rahway, NJ, USA, Pfizer, GlaxoSmithKline Intellectual Property Development, Sanofi US Services, Maze Therapeutics, Janssen Biotech, Novartis, and Boehringer Ingelheim. All Finnish biobanks are members of the BBMRI.fi infrastructure (https://www.bbmri.fi). The FINBB (https://finbb.fi/) is the coordinator of BBMRI-ERIC operations in Finland. The Finnish biobank data can be accessed through the Fingenious services (https://site.fingenious.fi/en/) managed by FINBB.

Competing interests statement

AP is the Chief Scientific Officer of the FinnGen project that is funded by thirteen pharmaceutical companies. HR was a full-time employee of Biogen during study planning and manuscript drafting and has stocks at Merck & Co. and Biogen Inc. MMF has received development funding from the Regional Council of Northern Savo and Business Finland for a data-driven tool related to memory disorders and healthcare decision tools, Charles River DRS Finland Ltd. and Orion Pharma have donated equipment for nonclinical cognition testing at the University of Eastern Finland. PI-M has received funding from Orion Research Foundation and Helsinki Biomedicum Foundation outside the present work. RK declares funding paid to the institution from Academy of Finland,

For peer review only

Government research funding, Saastamoinen Foundation, Vaajasalo foundation and Jane and Aatos Erkko foundation outside the present work; consulting fees from Orion Pharma; payment or honoraria from Angelini Pharma, Jazz Pharma, Lundbeck, Eisai, Orion Pharma, OmaMedical, Takeda, UCB; participation in monitoring or advisory board from Marinus Pharma and UCB; and leadership or fiduciary role in European Academy of Neurology Epilepsy scientific panel management group, European Epilepsy Reference network Epicare Steering Group and International League Against Epilepsy Career Development Commission. RS declares stocks or stock options at Orion Pharma. S-KH declares payment or honoraria and support for attending meetings or travel from Roche, consulting fees and participation in monitoring or advisory board from Novartis. TK declares payment or honoraria from Novartis Finland and Bayer Nordic SE. The authors declare no other competing financial or non-financial Interests.

References

1. Nichols E, Szoeke CEI, Vollset SE, et al. Global, regional, and national burden of Alzheimer's disease and other dementias, 1990–2016: a systematic analysis for the Global Burden of Disease Study 2016. *Lancet Neurol*. 2019;18(1):88-106. doi:10.1016/S1474-4422(18)30403-4

2. Prince M, Bryce R, Albanese E, Wimo A, Ribeiro W, Ferri CP. The global prevalence of dementia: A systematic review and metaanalysis. *Alzheimers Dement*. 2013;9(1):63-75.e2. doi:10.1016/j.jalz.2012.11.007

3. Official Statistics of Finland (OSF). Causes of death [online publication]. ISSN=1799-5078. Published online 2023. Accessed September 29, 2023. https://www.stat.fi/en/statistics/ksyyt

4. Malzbender K, Lavin-Mena L, Hughes L, Bose N, Goldman D, Patel D. Key Barriers to Clinical Trials for Alzheimer's Disease. Published online 2020. https://healthpolicy.usc.edu/research/key-barriers-for-clinical-trialsfor- alzheimers-disease/

5. Jack CR, Bennett DA, Blennow K, et al. NIA-AA Research Framework: Toward a biological definition of Alzheimer's disease. *Alzheimers Dement*. 2018;14(4):535-562. doi:10.1016/j.jalz.2018.02.018

6. Hansson O, Edelmayer RM, Boxer AL, et al. The Alzheimer's Association appropriate use recommendations for blood biomarkers in Alzheimer's disease. *Alzheimers Dement*. 2022;18(12):2669-2686. doi:10.1002/alz.12756

For peer review only

For peer review only - http://bmjopen.bmj.com/site/about/guidelines.xhtml

7. Ferreira PCL, Zhang Y, Snitz B, et al. Plasma biomarkers identify older adults at risk of Alzheimer’s disease and related dementias in a real-world population-based cohort. *Alzheimers Dement*. Published online March 6, 2023:alz.12986. doi:10.1002/alz.12986

8. Kurki MI, Karjalainen J, Palta P, et al. FinnGen provides genetic insights from a well-phenotyped isolated population. *Nature*. 2023;613(7944):508-518. doi:10.1038/s41586-022-05473-8

9. Julkunen V, Schwarz C, Kalapudas J, et al. A FinnGen pilot clinical recall study for Alzheimer’s disease. *Sci Rep*. 2023;13(1):12641. doi:10.1038/s41598-023-39835-7

10. Langbaum JB, Zissimopoulos J, Au R, et al. Recommendations to address key recruitment challenges of Alzheimer’s disease clinical trials. *Alzheimers Dement*. 2023;19(2):696-707. doi:10.1002/alz.12737

11. Zetterberg H, Blennow K. Blood Biomarkers: Democratizing Alzheimer’s Diagnostics. *Neuron*. 2020;106(6):881-883. doi:10.1016/j.neuron.2020.06.004

12. Husain M. Screening for Alzheimer’s disease: plasma biomarkers and what else? *Brain*. 2023;146(2):407-408. doi:10.1093/brain/awac490

13. Rodosthenous RS, Niemi MEK, Kallio L, et al. Recontacting biobank participants to collect lifestyle, behavioural and cognitive information via online questionnaires: lessons from a pilot study within FinnGen. *BMJ Open*. 2022;12(10):e064695. doi:10.1136/bmjopen-2022-064695

14. Kaprio J, Bollepalli S, Buchwald J, et al. The Older Finnish Twin Cohort — 45 Years of Follow-up. *Twin Res Hum Genet*. 2019;22(4):240-254. doi:10.1017/thg.2019.54

15. Tuomela J, Kaprio J, Sipilä PN, et al. Accuracy of self-reported anthropometric measures — Findings from the Finnish Twin Study. *Obes Res Clin Pract*. 2019;13(6):522-528. doi:10.1016/j.orcp.2019.10.006

16. Lindgren N, Rinne JO, Palviainen T, Kaprio J, Vuoksimaa E. Prevalence and correlates of dementia and mild cognitive impairment classified with different versions of the modified Telephone Interview for Cognitive Status (TICS-m). *Int J Geriatr Psychiatry*. 2019;34(12):1883-1891. doi:10.1002/gps.5205

17. Huang Y, Ollikainen M, Muniandy M, et al. Supplementary Materials: Identification, Heritability, and Relation With Gene Expression of Novel DNA Methylation Loci for Blood Pressure. *Hypertension*. 2020;76(1):195-205. doi:10.1161/HYPERTENSIONAHA.120.14973

18. Gatz M, Reynolds C, Nikolic J, Lowe B, Karel M, Pedersen N. An Empirical Test of Telephone Screening to Identify Potential Dementia Cases. *Int Psychogeriatr*. 1995;7(3):429-438. doi:10.1017/S1041610295002171

19. Brandt J, Spencer M, Folstein M. The Telephone Interview for Cognitive Status. Published online 1988.

20. Järvenpää T, Rinne JO, Räihä I, et al. Characteristics of Two Telephone Screens for Cognitive Impairment. *Dement Geriatr Cogn Disord*. 2002;13(3):149-155. doi:10.1159/000048646

For peer review only

For peer review only - http://bmjopen.bmj.com/site/about/guidelines.xhtml

21. Lindgren N, Kaprio J, Rinne JO, Vuoksimaa E. Immediate verbal recall and familial dementia risk: population-based study of over 4000 twins. *J Neurol Neurosurg Psychiatry*. 2019;90(1):90-97. doi:10.1136/jnnp-2018-319122

22. Rhodius-Meester HFM, Paajanen T, Koikkalainen J, et al. cCOG: A web-based cognitive test tool for detecting neurodegenerative disorders. *Alzheimers Dement Diagn Assess Dis Monit*. 2020;12(1). doi:10.1002/dad2.12083

23. Van Gils AM, Van De Beek M, Van Unnik AAJM, et al. Optimizing cCOG, a Web-based tool, to detect dementia with Lewy Bodies. *Alzheimers Dement Diagn Assess Dis Monit*. 2022;14(1):e12379. doi:10.1002/dad2.12379

24. Morris JC, Heyman A, Mohs RC, et al. The Consortium to Establish a Registry for Alzheimer's Disease (CERAD): Part I. Clinical and neuropsychological assessment of Alzheimer's disease. *Neurology*. Published online 1989. doi:10.1212/wnl.39.9.1159

25. Hazan E, Frankenburg F, Brenkel M, Shulman K. The test of time: a history of clock drawing. *Int J Geriatr Psychiatry*. 2018;33(1). doi:10.1002/gps.4731

26. Hallikainen I, Alenius M, Hokkanen L, et al. CERAD-tehtäväsarjaan koulutustason huomioivat katkaisurajat ja kokonaispistemäärä käyttöön. *Suom Lääkärilehti*. 2023;78(e35809).

27. Weschler D. WMS-III - Wechsler Memory Scale - 3rd ed. Published online 2008.

28. Armitage SG. An Analysis of Certain Psychological Tests Used for the Evaluation of Brain Injury. Published online 1946.

29. Stroop JR. Studies of inference in serial verbal reactions. *J Exp Psychol*. 1935;18:643-662.

30. Ngandu T, Lehtisalo J, Solomon A, et al. A 2 year multidomain intervention of diet, exercise, cognitive training, and vascular risk monitoring versus control to prevent cognitive decline in at-risk elderly people (FINGER): a randomised controlled trial. *The Lancet*. 2015;385(9984):2255-2263. doi:10.1016/S0140-6736(15)60461-5

31. Karikari TK, Pascoal TA, Ashton NJ, et al. Blood phosphorylated tau 181 as a biomarker for Alzheimer's disease: a diagnostic performance and prediction modelling study using data from four prospective cohorts. *Lancet Neurol*. 2020;19(5):422-433. doi:10.1016/S1474-4422(20)30071-5

32. Thijssen EH, Verberk IMW, Kindermans J, et al. Differential diagnostic performance of a panel of plasma biomarkers for different types of dementia. *Alzheimers Dement Diagn Assess Dis Monit*. 2022;14(1):e12285. doi:10.1002/dad2.12285

33. Ashton NJ, Brum WS, Di Molfetta G, et al. Diagnostic Accuracy of the Plasma ALZpath PTau217 Immunoassay to Identify Alzheimer's Disease Pathology. Neurology; 2023. doi:10.1101/2023.07.11.23292493

34. Vuoksimaa E, Palviainen T, Lindgren N, Rinne JO, Kaprio J. Accuracy of Imputation for Apolipoprotein E ε Alleles in Genome-Wide Genotyping Data. *JAMA Netw Open*. 2020;3(1):e1919960. doi:10.1001/jamanetworkopen.2019.19960

For peer review only

35. Bellenguez C. New insights into the genetic etiology of Alzheimer's disease and related dementias. *Nat Genet*. 2022;54.

36. Livingston G, Huntley J, Sommerlad A, et al. Dementia prevention, intervention, and care: 2020 report of the Lancet Commission. *The Lancet*. 2020;396(10248):413-446. doi:10.1016/S0140-6736(20)30367-6

37. Husu P, Tokola K, Vähä-Ypyä H, et al. Physical Activity, Sedentary Behavior, and Time in Bed Among Finnish Adults Measured 24/7 by Triaxial Accelerometry. *J Meas Phys Behav*. 2021;4(2):163-173. doi:10.1123/jmpb.2020-0056

38. Matthews CE, Hagströmer M, Pober DM, Bowles HR. Best Practices for Using Physical Activity Monitors in Population-Based Research. *Med Sci Sports Exerc*. 2012;44(1S):S68-S76. doi:10.1249/MSS.0b013e3182399e5b

39. Husu P, Suni J, Vähä-Ypyä H, et al. Objectively measured sedentary behavior and physical activity in a sample of Finnish adults: a cross-sectional study. *BMC Public Health*. 2016;16(1):920. doi:10.1186/s12889-016-3591-y

40. Vähä-Ypyä H, Husu P, Suni J, Vasankari T, Sievänen H. Reliable recognition of lying, sitting, and standing with a hip-worn accelerometer. *Scand J Med Sci Sports*. 2018;28(3):1092-1102. doi:10.1111/sms.13017

41. Vähä-Ypyä H, Vasankari T, Husu P, Suni J, Sievänen H. A universal, accurate intensity-based classification of different physical activities using raw data of accelerometer. *Clin Physiol Funct Imaging*. 2015;35(1):64-70. doi:10.1111/cpf.12127

42. Vähä-Ypyä H, Vasankari T, Husu P, et al. Validation of Cut-Points for Evaluating the Intensity of Physical Activity with Accelerometry-Based Mean Amplitude Deviation (MAD). Miller PJO, ed. *PLOS ONE*. 2015;10(8):e0134813. doi:10.1371/journal.pone.0134813

43. Altini M, Kinnunen H. The Promise of Sleep: A Multi-Sensor Approach for Accurate Sleep Stage Detection Using the Oura Ring. *Sensors*. 2021;21(13):4302. doi:10.3390/s21134302

44. Henriksen A, Svartdal F, Grimsgaard S, Hartvigsen G, Hopstock LA. Polar Vantage and Oura Physical Activity and Sleep Trackers: Validation and Comparison Study. *JMIR Form Res*. 2022;6(5):e27248. doi:10.2196/27248

45. Radloff LS. The CES-D Scale: A Self-Report Depression Scale for Research in the General Population. *Appl Psychol Meas*. 1977;1(3):385-401. doi:10.1177/014662167700100306

46. Saari TT, Piirtola M, Aaltonen A, et al. Self-Reported Depression Symptoms in Old Age across Cohorts and Cognitive Spectrum and Associations with Genetic Risk. PsyArXiv; 2023. doi:10.31234/osf.io/jvmtz

47. Ryff CD. Psychological Well-Being Revisited: Advances in the Science and Practice of Eudaimonia. *Psychother Psychosom*. 2014;83(1):10-28. doi:10.1159/000353263

48. Ryff CD. Happiness Is Everything, or Is It? Explorations on the Meaning of Psychological Well-Being. *J Pers Soc Psychol*. 1989;57(6):1069-1801.

For peer review only

49. Floderus B. Psycho-social factors in relation to coronary heart disease and associated risk factors. *Nord Hyg Tidskr*. 1974;(Suppl. 6).

50. Rose RJ, Koskenvuo M, Kaprio J, Sarna S, Langinvainio H. Shared Genes, Shared Experiences,and Similarity of Personality: Data From 14,288 Adult Finnish Co-Twins. *J Pers Soc Psychol*. 1988;54(1):161-171.

51. Allardt E. About Dimensions of Welfare: Explanatory Analysis of the Comparative Scandinavian Survey. Research Group of Comparative Sociology. University of Helsinki; 1973.

52. Koivumaa-Honkanen H, Kaprio J, Honkanen R, Viinam�ki H, Koskenvuo M. Life satisfaction and depression in a 15-year follow-up of healthy adults. *Soc Psychiatry Psychiatr Epidemiol*. 2004;39(12):994-999. doi:10.1007/s00127-004-0833-6

53. Park LQ, Gross AL, McLaren DG, et al. Confirmatory factor analysis of the ADNI neuropsychological battery. *Brain Imaging Behav*. 2012;6(4):528-539. doi:10.1007/s11682-012-9190-3

54. Dowling NM, Hermann B, La Rue A, Sager MA. Latent structure and factorial invariance of a neuropsychological test battery for the study of preclinical Alzheimer’s disease. *Neuropsychology*. 2010;24(6):742-756. doi:10.1037/a0020176

55. Varjonen A, Schwarz C, Vuoksimaa E. Co-twin design in brain imaging—review on biomarkers of Alzheimer’s disease. *Cereb Cortex*. 2023;33(14):9054-9066. doi:10.1093/cercor/bhad181

For peer review only

For peer review only - http://bmjopen.bmj.com/site/about/guidelines.xhtml

FinnGen Collaborators

Full Name	Affiliation	E-mail	Role 1	Role 2
Aarno Palotie	Institute for Molecular Medicine Finland (FIMM), HiLIFE, University of Helsinki, Helsinki, Finland; Broad Institute of MIT and Harvard; Massachusetts General Hospital	aarno.palotie@helsinki.fi	Steering Committee	Steering Committee
Mark Daly	Institute for Molecular Medicine Finland (FIMM), HiLIFE, University of Helsinki, Helsinki, Finland; Broad Institute of MIT and Harvard; Massachusetts General Hospital	mark.daly@helsinki.fi	Steering Committee	Steering Committee
Bridget Riley-Gills	Abbvie, Chicago, IL, United States	bridget.rileygillis@abbvie.com	Steering Committee	Pharmaceutical companies
Howard Jacob	Abbvie, Chicago, IL, United States	howard.jacob@abbvie.com	Steering Committee	Pharmaceutical companies
Dirk Paul	Astra Zeneca, Cambridge, United Kingdom	dirk.paul@astrazeneca.com	Steering Committee	Pharmaceutical companies
Athena Matakidou	Astra Zeneca, Cambridge, United Kingdom	athena.x.matakidou@gsk.com	Steering Committee	Pharmaceutical companies

For peer review only - http://bmjopen.bmj.com/site/about/guidelines.xhtml

Adam Platt	Astra Zeneca, Cambridge, United Kingdom	adam.platt@astrazeneca.com	Steering Committee	Pharmaceutical companies
Heiko Runz	Biogen, Cambridge, MA, United States	heiko.runz@biogen.com	Steering Committee	Pharmaceutical companies
Sally John	Biogen, Cambridge, MA, United States	sally.john@biogen.com	Steering Committee	Pharmaceutical companies
George Okafo	Boehringer Ingelheim, Ingelheim am Rhein, Germany	george.okafo@boehringer-ingelheim.com	Steering Committee	Pharmaceutical companies
Nathan Lawless	Boehringer Ingelheim, Ingelheim am Rhein, Germany	nathan.lawless@boehringer-ingelheim.com	Steering Committee	Pharmaceutical companies
Heli Salminen-Mankonen	Boehringer Ingelheim, Ingelheim am Rhein, Germany	heli.salminen-mankonen@boehringer-ingelheim.com	Steering Committee	Pharmaceutical companies
Robert Plenge	Bristol Myers Squibb, New York, NY, United States	robert.plenge@bms.com	Steering Committee	Pharmaceutical companies
Joseph Maranville	Bristol Myers Squibb, New York, NY, United States	joseph.maranville@bms.com	Steering Committee	Pharmaceutical companies
Mark McCarthy	Genentech, San Francisco, CA, United States	mccarthy.mark@gene.com	Steering Committee	Pharmaceutical companies

For peer review only

Julie Hunkapiller	Genentech, San Francisco, CA, United States	hunkapiller.julie@gene.com	Steering Committee	Pharmaceutical companies
Margaret G. Ehm	GlaxoSmithKline, Collegeville, PA, United States	meg.g.ehm@gsk.com	Steering Committee	Pharmaceutical companies
Kirsi Auro	GlaxoSmithKline, Espoo, Finland	kirsi.m.auro@gsk.com	Steering Committee	Pharmaceutical companies
Simonne Longerich	Merck, Kenilworth, NJ, United States	simonne.longerich@merck.com	Steering Committee	Pharmaceutical companies
Caroline Fox	Merck, Kenilworth, NJ, United States	caroline.fox@merck.com	Steering Committee	Pharmaceutical companies
Anders Mälarstig	Pfizer, New York, NY, United States	anders.malarstig@pfizer.com	Steering Committee	Pharmaceutical companies
Katherine Klinger	Translational Sciences, Sanofi R&D, Framingham, MA, USA	katherine.klinger@sanofi.com	Steering Committee	Pharmaceutical companies
Deepak Raipal	Translational Sciences, Sanofi R&D, Framingham, MA, USA	deepak.rajpal@sanofi.com	Steering Committee	Pharmaceutical companies
Eric Green	Maze Therapeutics, San Francisco, CA, United States	egreen@mazetx.com	Steering Committee	Pharmaceutical companies
Robert Graham	Maze Therapeutics, San Francisco, CA, United States	rgraham@mazetx.com	Steering Committee	Pharmaceutical companies
Robert Yang	Janssen Biotech, Beerse, Belgium	ryang31@its.jnj.com	Steering Committee	Pharmaceutical companies

For peer review only

For peer review only - http://bmjopen.bmj.com/site/about/guidelines.xhtml

Chris O´Donnell	Novartis Institutes for BioMedical Research, Cambridge, MA, United States	chris.odonnell@novartis.com	Steering Committee	Pharmaceutical companies
Tomi P. Mäkelä	HiLIFE, University of Helsinki, Finland, Finland	tomi.makela@helsinki.fi	Steering Committee	University of Helsinki & Biobanks
Jaakko Kaprio	Institute for Molecular Medicine Finland (FIMM), HiLIFE, University of Helsinki, Helsinki, Finland	jaakko.kaprio@helsinki.fi	Steering Committee	University of Helsinki & Biobanks
Petri Virolainen	Auria Biobank / University of Turku / Hospital District of Southwest Finland, Turku, Finland	petri.virolainen@tyks.fi	Steering Committee	University of Helsinki & Biobanks
Antti Hakanen	Auria Biobank / University of Turku / Hospital District of Southwest Finland, Turku, Finland	antti.hakanen@tyks.fi	Steering Committee	University of Helsinki & Biobanks
Terhi Kilpi	THL Biobank / Finnish Institute for Health and Welfare (THL), Helsinki, Finland	terhi.kilpi@thl.fi	Steering Committee	University of Helsinki & Biobanks
Markus Perola	THL Biobank / Finnish Institute for Health and Welfare (THL), Helsinki, Finland	markus.perola@thl.fi	Steering Committee	University of Helsinki & Biobanks

For peer review only

Jukka Partanen	Finnish Red Cross Blood Service / Finnish Hematology Registry and Clinical Biobank, Helsinki, Finland	jukka.partanen@veripalvelu.fi	Steering Committee	University of Helsinki & Biobanks
Anne Pitkäranta	Helsinki Biobank / Helsinki University and Hospital District of Helsinki and Uusimaa, Helsinki	anne.pitkaranta@hus.fi	Steering Committee	University of Helsinki & Biobanks
Taneli Raivio	Helsinki Biobank / Helsinki University and Hospital District of Helsinki and Uusimaa, Helsinki	taneli.raivio@hus.fi	Steering Committee	University of Helsinki & Biobanks
Jani Tikkanen	Northern Finland Biobank Borealis / University of Oulu / Wellbeing Services county of North Ostrobothnia , Oulu, Finland	jani.tikkanen@pohde.fi	Steering Committee	University of Helsinki & Biobanks
Raisa Serpi	Northern Finland Biobank Borealis / University of Oulu / Northern Ostrobothnia Hospital District, Oulu, Finland	raisa.serpi@ppshp.fi	Steering Committee	University of Helsinki & Biobanks
Tarja Laitinen	Finnish Clinical Biobank Tampere / University of Tampere / The Wellbeing Services County of Pirkanmaa, Tampere, Finland	tarja.laitinen@pirha.fi	Steering Committee	University of Helsinki & Biobanks

For peer review only - http://bmjopen.bmj.com/site/about/guidelines.xhtml

Veli-Matti Kosma	Biobank of Eastern Finland / University of Eastern Finland / Northern Savo Hospital District, Kuopio, Finland	veli-matti.kosma@uef.fi	Steering Committee	University of Helsinki & Biobanks
Jari Laukkanen	Central Finland Biobank / University of Jyväskylä / Central Finland Health Care District, Jyväskylä, Finland	jari.laukkanen@ksshp.fi	Steering Committee	University of Helsinki & Biobanks
Marco Hautalahti	FINBB - Finnish biobank cooperative	marco.hautalahti@finbb.fi	Steering Committee	University of Helsinki & Biobanks
Outi Tuovila	Business Finland, Helsinki, Finland	outi.tuovila@businessfinland.fi	Steering Committee	Other Experts/ Non-Voting Members
Raimo Pakkanen	Business Finland, Helsinki, Finland	raimo.pakkanen@businessfinland.fi	Steering Committee	Other Experts/ Non-Voting Members
Jeffrey Waring	Abbvie, Chicago, IL, United States	jeff.waring@abbvie.com	Scientific Committee	Pharmaceutical companies
Bridget Riley-Gillis	Abbvie, Chicago, IL, United States	bridget.rileygillis@abbvie.com	Scientific Committee	Pharmaceutical companies
Fedik Rahimov	Abbvie, Chicago, IL, United States	fedik.rahimov@abbvie.com	Scientific Committee	Pharmaceutical companies

For peer review only

Ioanna Tachmazidou	Astra Zeneca, Cambridge, United Kingdom	ioanna.tachmazidou@astrazeneca.com	Scientific Committee	Pharmaceutical companies
Chia-Yen Chen	Biogen, Cambridge, MA, United States	chiayen.chen@biogen.com	Scientific Committee	Pharmaceutical companies
Heiko Runz	Biogen, Cambridge, MA, United States	heiko.runz@biogen.com	Scientific Committee	Pharmaceutical companies
Zhihao Ding	Boehringer Ingelheim, Ingelheim am Rhein, Germany	zhihao.ding@boehringer-ingelheim.com	Scientific Committee	Pharmaceutical companies
Marc Jung	Boehringer Ingelheim, Ingelheim am Rhein, Germany	marc_oliver.jung@boehringer-ingelheim.com	Scientific Committee	Pharmaceutical companies
Shameek Biswas	Bristol Myers Squibb, New York, NY, United States	Shameek.Biswas@bms.com	Scientific Committee	Pharmaceutical companies
Rion Pendergrass	Genentech, San Francisco, CA, United States	penders2@gene.com	Scientific Committee	Pharmaceutical companies
Julie Hunkapiller	Genentech, San Francisco, CA, United States	hunkapiller.julie@gene.com	Scientific Committee	Pharmaceutical companies
Margaret G. Ehm	GlaxoSmithKline, Collegeville, PA, United States	meg.g.ehm@gsk.com	Scientific Committee	Pharmaceutical companies
David Pulford	GlaxoSmithKline, Stevenage, United Kingdom	david.x.pulford@gsk.com	Scientific Committee	Pharmaceutical companies

For peer review only
For peer review only - http://bmjopen.bmj.com/site/about/guidelines.xhtml

Neha Raghavan	Merck, Kenilworth, NJ, United States	neha.raghavan@merck.com	Scientific Committee	Pharmaceutical companies
Adriana Huertas-Vazquez	Merck, Kenilworth, NJ, United States	adriana.huertas.vazquez@merck.com	Scientific Committee	Pharmaceutical companies
Jae-Hoon Sul	Merck, Kenilworth, NJ, United States	jae.hoon.sul@merck.com	Scientific Committee	Pharmaceutical companies
Anders Mälarstig	Pfizer, New York, NY, United States	anders.malarstig@pfizer.com	Scientific Committee	Pharmaceutical companies
Xinli Hu	Pfizer, New York, NY, United States	xinli.hu@pfizer.com	Scientific Committee	Pharmaceutical companies
Katherine Klinger	Translational Sciences, Sanofi R&D, Framingham, MA, USA	katherine.klinger@sanofi.com	Scientific Committee	Pharmaceutical companies
Robert Graham	Maze Therapeutics, San Francisco, CA, United States	rgraham@mazetx.com	Scientific Committee	Pharmaceutical companies
Eric Green	Maze Therapeutics, San Francisco, CA, United States	egreen@mazetx.com	Scientific Committee	Pharmaceutical companies
Sahar Mozaffari	Maze Therapeutics, San Francisco, CA, United States	smozaffari@mazetx.com	Scientific Committee	Pharmaceutical companies
Dawn Waterworth	Janssen Research & Development, LLC, Spring House, PA, United States	dwaterwo@its.jnj.com	Scientific Committee	Pharmaceutical companies

For peer review only - http://bmjopen.bmj.com/site/about/guidelines.xhtml

Nicole Renaud	Novartis Institutes for BioMedical Research, Cambridge, MA, United States	nicole.renaud@novartis.com	Scientific Committee	Pharmaceutical companies
Ma´en Obeidat	Novartis Institutes for BioMedical Research, Cambridge, MA, United States	maen.obeidat@novartis.com	Scientific Committee	Pharmaceutical companies
Samuli Ripatti	Institute for Molecular Medicine Finland (FIMM), HiLIFE, University of Helsinki, Helsinki, Finland	samuli.ripatti@helsinki.fi	Scientific Committee	University of Helsinki & Biobanks
Johanna Schleutker	Auria Biobank / Univ. of Turku / Hospital District of Southwest Finland, Turku, Finland	johanna.schleutker@utu.fi	Scientific Committee	University of Helsinki & Biobanks
Markus Perola	THL Biobank / Finnish Institute for Health and Welfare (THL), Helsinki, Finland	markus.perola@thl.fi	Scientific Committee	University of Helsinki & Biobanks
Mikko Arvas	Finnish Red Cross Blood Service / Finnish Hematology Registry and Clinical Biobank, Helsinki, Finland	mikko.arvas@veripalvelu.fi	Scientific Committee	University of Helsinki & Biobanks
Olli Carpén	Helsinki Biobank / Helsinki University and Hospital District of Helsinki and Uusimaa, Helsinki	olli.carpen@helsinki.fi	Scientific Committee	University of Helsinki & Biobanks

For peer review only - http://bmjopen.bmj.com/site/about/guidelines.xhtml

Reetta Hinttala	Northern Finland Biobank Borealis / University of Oulu / Northern Ostrobothnia Hospital District, Oulu, Finland	reetta.hinttala@oulu.fi	Scientific Committee	University of Helsinki & Biobanks
Johannes Kettunen	Northern Finland Biobank Borealis / University of Oulu / Northern Ostrobothnia Hospital District, Oulu, Finland	johannes.kettunen@oulu.fi	Scientific Committee	University of Helsinki & Biobanks
Arto Mannermaa	Biobank of Eastern Finland / University of Eastern Finland / Northern Savo Hospital District, Kuopio, Finland	arto.mannermaa@uef.fi	Scientific Committee	University of Helsinki & Biobanks
Katriina Aalto-Setälä	Faculty of Medicine and Health Technology, Tampere University, Tampere, Finland	katriina.aalto-setala@tuni.fi	Scientific Committee	University of Helsinki & Biobanks
Mika Kähönen	Finnish Clinical Biobank Tampere / University of Tampere / Pirkanmaa Hospital District, Tampere, Finland	mika.kahonen@uta.fi	Scientific Committee	University of Helsinki & Biobanks
Jari Laukkanen	Central Finland Biobank / University of Jyväskylä / Central Finland Health Care District, Jyväskylä, Finland	jari.laukkanen@ksshp.fi	Scientific Committee	University of Helsinki & Biobanks

For peer review only

For peer review only - http://bmjopen.bmj.com/site/about/guidelines.xhtml

Johanna Mäkelä	FINBB - Finnish biobank cooperative	johanna.makela@finbb.fi	Scientific Committee	University of Helsinki & Biobanks
Reetta Kälviäinen	Northern Savo Hospital District, Kuopio, Finland	reetta.kalviainen@kuh.fi	Clinical Groups	Neurology Group
Valtteri Julkunen	Northern Savo Hospital District, Kuopio, Finland	valtteri.julkunen@kuh.fi	Clinical Groups	Neurology Group
Hilkka Soininen	Northern Savo Hospital District, Kuopio, Finland	hilkka.soininen@uef.fi	Clinical Groups	Neurology Group
Anne Remes	Clinical Neurosciences, University of Helsinki, Helsinki, Finland	anne.remes@oulu.fi	Clinical Groups	Neurology Group
Mikko Hiltunen	University of Eastern Finland, Kuopio, Finland	mikko.hiltunen@uef.fi	Clinical Groups	Neurology Group
Jukka Peltola	Pirkanmaa Hospital District, Tampere, Finland	jukka.peltola@pshp.fi	Clinical Groups	Neurology Group
Minna Raivio	Hospital District of Helsinki and Uusimaa, Helsinki, Finland	minna.raivio@geri.fi	Clinical Groups	Neurology Group
Pentti Tienari	Hospital District of Helsinki and Uusimaa, Helsinki, Finland	pentti.tienari@hus.fi	Clinical Groups	Neurology Group
Juha Rinne	Hospital District of Southwest Finland, Turku, Finland	juha.rinne@tyks.fi	Clinical Groups	Neurology Group

For peer review only
For peer review only - http://bmjopen.bmj.com/site/about/guidelines.xhtml

Roosa Kallionpää	Hospital District of Southwest Finland, Turku, Finland	roosa.kallionpaa@tyks.fi	Clinical Groups	Neurology Group
Juulia Partanen	Institute for Molecular Medicine Finland, HiLIFE, University of Helsinki, Finland	juulia.partanen@helsinki.fi	Clinical Groups	Neurology Group
Ali Abbasi	Abbvie, Chicago, IL, United States	ali.abbasi@abbvie.com	Clinical Groups	Neurology Group
Adam Ziemann	Abbvie, Chicago, IL, United States	adam.ziemann@abbvie.com	Clinical Groups	Neurology Group
Nizar Smaoui	Abbvie, Chicago, IL, United States	nizar.smaoui@abbvie.com	Clinical Groups	Neurology Group
Anne Lehtonen	Abbvie, Chicago, IL, United States	anne.lehtonen@abbvie.com	Clinical Groups	Neurology Group
Susan Eaton	Biogen, Cambridge, MA, United States	susan.eaton@biogen.com	Clinical Groups	Neurology Group
Heiko Runz	Biogen, Cambridge, MA, United States	heiko.runz@biogen.com	Clinical Groups	Neurology Group
Sanni Lahdenperä	Biogen, Cambridge, MA, United States	sanni.lahdenpera@biogen.com	Clinical Groups	Neurology Group
Shameek Biswas	Bristol Myers Squibb, New York, NY, United States	shameek.biswas@bms.com	Clinical Groups	Neurology Group

For peer review only - http://bmjopen.bmj.com/site/about/guidelines.xhtml

Julie Hunkapiller	Genentech, San Francisco, CA, United States	hunkapiller.julie@gene.com	Clinical Groups	Neurology Group
Natalie Bowers	Genentech, San Francisco, CA, United States	bowersn1@gene.com	Clinical Groups	Neurology Group
Edmond Teng	Genentech, San Francisco, CA, United States	teng.edmond@gene.com	Clinical Groups	Neurology Group
Rion Pendergrass	Genentech, San Francisco, CA, United States	penders2@gene.com	Clinical Groups	Neurology Group
Fanli Xu	GlaxoSmithKline, Brentford, United Kingdom	chun-fang.2.xu@gsk.com	Clinical Groups	Neurology Group
David Pulford	GlaxoSmithKline, Stevenage, United Kingdom	david.x.pulford@gsk.com	Clinical Groups	Neurology Group
Kirsi Auro	GlaxoSmithKline, Espoo, Finland	kirsi.m.auro@gsk.com	Clinical Groups	Neurology Group
Laura Addis	GlaxoSmithKline, Brentford, United Kingdom	laura.x.addis@gsk.com	Clinical Groups	Neurology Group
John Eicher	GlaxoSmithKline, Brentford, United Kingdom	john.d.eicher@gsk.com	Clinical Groups	Neurology Group
Qingqin S Li	Janssen Research & Development, LLC, Titusville, NJ 08560, United States	QLi2@its.jnj.com	Clinical Groups	Neurology Group

For peer review only

For peer review only - http://bmjopen.bmj.com/site/about/guidelines.xhtml

Karen He	Janssen Research & Development, LLC, Spring House, PA, United States	khe2@its.jnj.com	Clinical Groups	Neurology Group
Ekaterina Khramtsova	Janssen Research & Development, LLC, Spring House, PA, United States	ekhramts@its.jnj.com	Clinical Groups	Neurology Group
Neha Raghavan	Merck, Kenilworth, NJ, United States	neha.raghavan@merck.com	Clinical Groups	Neurology Group
Martti Färkkilä	Hospital District of Helsinki and Uusimaa, Helsinki, Finland	martti.farkkila@hus.fi	Clinical Groups	Gastroenterology Group
Jukka Koskela	Hospital District of Helsinki and Uusimaa, Helsinki, Finland	jukka.koskela@helsinki.fi	Clinical Groups	Gastroenterology Group
Sampsa Pikkarainen	Hospital District of Helsinki and Uusimaa, Helsinki, Finland	sampsa.pikkarainen@hus.fi	Clinical Groups	Gastroenterology Group
Airi Jussila	Pirkanmaa Hospital District, Tampere, Finland	airi.jussila@pshp.fi	Clinical Groups	Gastroenterology Group
Katri Kaukinen	Pirkanmaa Hospital District, Tampere, Finland	katri.kaukinen@tuni.fi	Clinical Groups	Gastroenterology Group
Timo Blomster	Wellbeing Services county of North Ostrobothnia, Oulu, Finland	timo.blomster@ppshp.fi	Clinical Groups	Gastroenterology Group
Mikko Kiviniemi	Northern Savo Hospital District, Kuopio, Finland	mikko.kiviniemi@kuh.fi	Clinical Groups	Gastroenterology Group

For peer review only

Markku Voutilainen	Hospital District of Southwest Finland, Turku, Finland	markku.voutilainen@tyks.fi	Clinical Groups	Gastroenterology Group
Mark Daly	Institute for Molecular Medicine, Finland (FIMM), HiLIFE, University of Helsinki, Helsinki, Finland; Broad Institute of MIT and Harvard; Massachusetts General Hospital	mark.daly@helsinki.fi	Clinical Groups	Gastroenterology Group
Ali Abbasi	Abbvie, Chicago, IL, United States	ali.abbasi@abbvie.com	Clinical Groups	Gastroenterology Group
Jeffrey Waring	Abbvie, Chicago, IL, United States	jeff.waring@abbvie.com	Clinical Groups	Gastroenterology Group
Nizar Smaoui	Abbvie, Chicago, IL, United States	nizar.smaoui@abbvie.com	Clinical Groups	Gastroenterology Group
Fedik Rahimov	Abbvie, Chicago, IL, United States	fedik.rahimov@abbvie.com	Clinical Groups	Gastroenterology Group
Anne Lehtonen	Abbvie, Chicago, IL, United States	anne.lehtonen@abbvie.com	Clinical Groups	Gastroenterology Group
Tim Lu	Genentech, San Francisco, CA, United States	lut8@gene.com	Clinical Groups	Gastroenterology Group
Natalie Bowers	Genentech, San Francisco, CA, United States	bowersn1@gene.com	Clinical Groups	Gastroenterology Group

For peer review only - http://bmjopen.bmj.com/site/about/guidelines.xhtml

Rion Pendergrass	Genentech, San Francisco, CA, United States	penders2@gene.com	Clinical Groups	Gastroenterology Group
Linda McCarthy	GlaxoSmithKline, Brentford, United Kingdom	linda.c.mccarthy@gsk.com	Clinical Groups	Gastroenterology Group
Amy Hart	Janssen Research & Development, LLC, Spring House, PA, United States	ahart13@its.jnj.com	Clinical Groups	Gastroenterology Group
Meijian Guan	Janssen Research & Development, LLC, Spring House, PA, United States	mguan4@its.jnj.com	Clinical Groups	Gastroenterology Group
Jason Miller	Merck, Kenilworth, NJ, United States	jason.miller4@merck.com	Clinical Groups	Gastroenterology Group
Kirsi Kalpala	Pfizer, New York, NY, United States	kirsi.kalpala@pfizer.com	Clinical Groups	Gastroenterology Group
Melissa Miller	Pfizer, New York, NY, United States	melissa.r.miller@pfizer.com	Clinical Groups	Gastroenterology Group
Xinli Hu	Pfizer, New York, NY, United States	xinli.hu@pfizer.com	Clinical Groups	Gastroenterology Group
Kari Eklund	Hospital District of Helsinki and Uusimaa, Helsinki, Finland	kari.eklund@hus.fi	Clinical Groups	Rheumatology Group
Antti Palomäki	Hospital District of Southwest Finland, Turku, Finland	ajpalo@utu.fi	Clinical Groups	Rheumatology Group

For peer review only - http://bmjopen.bmj.com/site/about/guidelines.xhtml

Pia Isomäki	Pirkanmaa Hospital District, Tampere, Finland	pia.isomaki@pshp.fi	Clinical Groups	Rheumatology Group
Laura Pirilä	Hospital District of Southwest Finland, Turku, Finland	laura.pirila@fimnet.fi,laura.pirila@tyks.fi	Clinical Groups	Rheumatology Group
Oili Kaipiainen-Seppänen	Northern Savo Hospital District, Kuopio, Finland	oili.kaipiainen-seppanen@kuh.fi	Clinical Groups	Rheumatology Group
Johanna Huhtakangas	Department of Internal Medicine, Kuopio University Hospital, Kuopio, Finland	johanna.huhtakangas@kuh.fi	Clinical Groups	Rheumatology Group
Nina Mars	Institute for Molecular Medicine Finland (FIMM), HiLIFE, University of Helsinki, Helsinki, Finland	nina.mars@helsinki.fi	Clinical Groups	Rheumatology Group
Ali Abbasi	Abbvie, Chicago, IL, United States	ali.abbasi@abbvie.com	Clinical Groups	Rheumatology Group
Jeffrey Waring	Abbvie, Chicago, IL, United States	jeff.waring@abbvie.com	Clinical Groups	Rheumatology Group
Fedik Rahimov	Abbvie, Chicago, IL, United States	fedik.rahimov@abbvie.com	Clinical Groups	Rheumatology Group
Apinya Lertratanakul	Abbvie, Chicago, IL, United States	apinya.lertratanakul@abbvie.com	Clinical Groups	Rheumatology Group

For peer review only

Nizar Smaoui	Abbvie, Chicago, IL, United States	nizar.smaoui@abbvie.com	Clinical Groups	Rheumatology Group
Anne Lehtonen	Abbvie, Chicago, IL, United States	anne.lehtonen@abbvie.com	Clinical Groups	Rheumatology Group
Marla Hochfeld	Bristol Myers Squibb, New York, NY, United States	mhochfeld@celgene.com	Clinical Groups	Rheumatology Group
Natalie Bowers	Genentech, San Francisco, CA, United States	bowersn1@gene.com	Clinical Groups	Rheumatology Group
Rion Pendergrass	Genentech, San Francisco, CA, United States	penders2@gene.com	Clinical Groups	Rheumatology Group
Jorge Esparza Gordillo	GlaxoSmithKline, Brentford, United Kingdom	jorge.x.esparza-gordillo@gsk.com	Clinical Groups	Rheumatology Group
Kirsi Auro	GlaxoSmithKline, Espoo, Finland	kirsi.m.auro@gsk.com	Clinical Groups	Rheumatology Group
Dawn Waterworth	Janssen Research & Development, LLC, Spring House, PA, United States	dwaterwo@its.jnj.com	Clinical Groups	Rheumatology Group
Fabiana Farias	Merck, Kenilworth, NJ, United States	fabiana.farias@merck.com	Clinical Groups	Rheumatology Group
Kirsi Kalpala	Pfizer, New York, NY, United States	kirsi.kalpala@pfizer.com	Clinical Groups	Rheumatology Group

For peer review only - http://bmjopen.bmj.com/site/about/guidelines.xhtml

Nan Bing	Pfizer, New York, NY, United States	nan.bing@pfizer.com	Clinical Groups	Rheumatology Group
Xinli Hu	Pfizer, New York, NY, United States	xinli.hu@pfizer.com	Clinical Groups	Rheumatology Group
Tarja Laitinen	The Wellbeing Services County of Pirkanmaa, Tampere, Finland	tarja.laitinen@pirha.fi	Clinical Groups	Pulmonology Group
Margit Pelkonen	Northern Savo Hospital District, Kuopio, Finland	margit.pelkonen@kuh.fi	Clinical Groups	Pulmonology Group
Paula Kauppi	Hospital District of Helsinki and Uusimaa, Helsinki, Finland	paula.kauppi@hus.fi	Clinical Groups	Pulmonology Group
Hannu Kankaanranta	University of Gothenburg, Gothenburg, Sweden/ Seinäjoki Central Hospital, Seinäjoki, Finland/ Tampere University, Tampere, Finland	hannu.kankaanranta@tuni.fi	Clinical Groups	Pulmonology Group
Terttu Harju	Northern Ostrobothnia Hospital District, Oulu, Finland	terttu.harju@oulu.fi	Clinical Groups	Pulmonology Group
Riitta Lahesmaa	Hospital District of Southwest Finland, Turku, Finland	rilahes@utu.fi	Clinical Groups	Pulmonology Group
Nizar Smaoui	Abbvie, Chicago, IL, United States	nizar.smaoui@abbvie.com	Clinical Groups	Pulmonology Group

For peer review only - http://bmjopen.bmj.com/site/about/guidelines.xhtml

Glenda Lassi	Astra Zeneca, Cambridge, United Kingdom	glenda.lassi@astrazeneca.com	Clinical Groups	Pulmonology Group
Susan Eaton	Biogen, Cambridge, MA, United States	susan.eaton@biogen.com	Clinical Groups	Pulmonology Group
Hubert Chen	Genentech, San Francisco, CA, United States	chenh37@gene.com	Clinical Groups	Pulmonology Group
Rion Pendergrass	Genentech, San Francisco, CA, United States	penders2@gene.com	Clinical Groups	Pulmonology Group
Natalie Bowers	Genentech, San Francisco, CA, United States	bowersn1@gene.com	Clinical Groups	Pulmonology Group
Joanna Betts	GlaxoSmithKline, Brentford, United Kingdom	joanna.c.betts@gsk.com	Clinical Groups	Pulmonology Group
Kirsi Auro	GlaxoSmithKline, Espoo, Finland	kirsi.m.auro@gsk.com	Clinical Groups	Pulmonology Group
Rajashree Mishra	GlaxoSmithKline, Brentford, United Kingdom	rajashree.x.mishra@gsk.com	Clinical Groups	Pulmonology Group
Majd Mouded	Novartis, Basel, Switzerland	majd.mouded@novartis.com	Clinical Groups	Pulmonology Group
Debby Ngo	Novartis, Basel, Switzerland	debby.ngo@novartis.com	Clinical Groups	Pulmonology Group

For peer review only

For peer review only - http://bmjopen.bmj.com/site/about/guidelines.xhtml

Teemu Niiranen	Finnish Institute for Health and Welfare (THL), Helsinki, Finland	teemu.niiranen@thl.fi	Clinical Groups	Cardiometabolic Diseases Group
Felix Vaura	Finnish Institute for Health and Welfare (THL), Helsinki, Finland	fechva@utu.fi	Clinical Groups	Cardiometabolic Diseases Group
Veikko Salomaa	Finnish Institute for Health and Welfare (THL), Helsinki, Finland	veikko.salomaa@thl.fi	Clinical Groups	Cardiometabolic Diseases Group
Kaj Metsärinne	Hospital District of Southwest Finland, Turku, Finland	kaj.metsarinne@tyks.fi	Clinical Groups	Cardiometabolic Diseases Group
Jenni Aittokallio	Hospital District of Southwest Finland, Turku, Finland	jemato@utu.fi	Clinical Groups	Cardiometabolic Diseases Group
Mika Kähönen	Pirkanmaa Hospital District, Tampere, Finland	mika.kahonen@uta.fi	Clinical Groups	Cardiometabolic Diseases Group
Jussi Hernesniemi	Pirkanmaa Hospital District, Tampere, Finland	jussi.hernesniemi@tuni.fi	Clinical Groups	Cardiometabolic Diseases Group

For peer review only
For peer review only - http://bmjopen.bmj.com/site/about/guidelines.xhtml

Daniel Gordin	Hospital District of Helsinki and Uusimaa, Helsinki, Finland	daniel.gordin@hus.fi	Clinical Groups	Cardiometabolic Diseases Group
Juha Sinisalo	Hospital District of Helsinki and Uusimaa, Helsinki, Finland	juha.sinisalo@hus.fi	Clinical Groups	Cardiometabolic Diseases Group
Marja-Riitta Taskinen	Hospital District of Helsinki and Uusimaa, Helsinki, Finland	marja-riitta.taskinen@helsinki.fi	Clinical Groups	Cardiometabolic Diseases Group
Tiinamaija Tuomi	Hospital District of Helsinki and Uusimaa, Helsinki, Finland	tiinamaija.tuomi@hus.fi	Clinical Groups	Cardiometabolic Diseases Group
Timo Hiltunen	Hospital District of Helsinki and Uusimaa, Helsinki, Finland	timo.hiltunen@hus.fi	Clinical Groups	Cardiometabolic Diseases Group
Jari Laukkanen	Central Finland Health Care District, Jyväskylä, Finland	jari.laukkanen@ksshp.fi	Clinical Groups	Cardiometabolic Diseases Group
Amanda Elliott	Institute for Molecular Medicine Finland (FIMM), HiLIFE, University of Helsinki, Helsinki, Finland; Broad Institute, Cambridge, MA, USA and	aelliott@broadinstitute.org	Clinical Groups	Cardiometabolic Diseases Group

For peer review only

	Massachusetts General Hospital, Boston, MA, USA			
Mary Pat Reeve	Institute for Molecular Medicine Finland (FIMM), HiLIFE, University of Helsinki, Helsinki, Finland	mary.reeve@helsinki.fi	Clinical Groups	Cardiometabolic Diseases Group
Sanni Ruotsalainen	Institute for Molecular Medicine Finland (FIMM), HiLIFE, University of Helsinki, Helsinki, Finland	sanni.ruotsalainen@helsinki.fi	Clinical Groups	Cardiometabolic Diseases Group
Benjamin Challis	Astra Zeneca, Cambridge, United Kingdom	benjamin.challis@astrazeneca.com	Clinical Groups	Cardiometabolic Diseases Group
Dirk Paul	Astra Zeneca, Cambridge, United Kingdom	dirk.paul@astrazeneca.com	Clinical Groups	Cardiometabolic Diseases Group
Julie Hunkapiller	Genentech, San Francisco, CA, United States	hunkapiller.julie@gene.com	Clinical Groups	Cardiometabolic Diseases Group
Natalie Bowers	Genentech, San Francisco, CA, United States	bowersn1@gene.com	Clinical Groups	Cardiometabolic Diseases Group

For peer review only

Rion Pendergrass	Genentech, San Francisco, CA, United States	penders2@gene.com	Clinical Groups	Cardiometabolic Diseases Group
Audrey Chu	GlaxoSmithKline, Brentford, United Kingdom	audrey.y.chu@gsk.com	Clinical Groups	Cardiometabolic Diseases Group
Kirsi Auro	GlaxoSmithKline, Espoo, Finland	kirsi.m.auro@gsk.com	Clinical Groups	Cardiometabolic Diseases Group
Dermot Reilly	Janssen Research & Development, LLC, Boston, MA, United States	dreill11@its.jnj.com	Clinical Groups	Cardiometabolic Diseases Group
Mike Mendelson	Novartis, Boston, MA, United States	mike.mendelson@novartis.com	Clinical Groups	Cardiometabolic Diseases Group
Jaakko Parkkinen	Pfizer, New York, NY, United States	jaakko.parkkinen@pfizer.com	Clinical Groups	Cardiometabolic Diseases Group
Melissa Miller	Pfizer, New York, NY, United States	melissa.r.miller@pfizer.com	Clinical Groups	Cardiometabolic Diseases Group
Tuomo Meretoja	Hospital District of Helsinki and Uusimaa, Helsinki, Finland	tuomo.meretoja@hus.fi	Clinical Groups	Oncology Group

For peer review only

Heikki Joensuu	Hospital District of Helsinki and Uusimaa, Helsinki, Finland	heikki.joensuu@hus.fi	Clinical Groups	Oncology Group
Olli Carpén	Hospital District of Helsinki and Uusimaa, Helsinki, Finland	olli.carpen@helsinki.fi	Clinical Groups	Oncology Group
Johanna Mattson	Hospital District of Helsinki and Uusimaa, Helsinki, Finland	johanna.mattson@hus.fi	Clinical Groups	Oncology Group
Eveliina Salminen	Hospital District of Helsinki and Uusimaa, Helsinki, Finland	eveliina.e.salminen@hus.fi	Clinical Groups	Oncology Group
Annika Auranen	Pirkanmaa Hospital District , Tampere, Finland	annika.auranen@pirha.fi	Clinical Groups	Oncology Group
Peeter Karihtala	Helsinki University Hospital Comprehensive Cancer Centre, Helsinki, Finland	peeter.karihtala@oulu.fi	Clinical Groups	Oncology Group
Päivi Auvinen	Northern Savo Hospital District, Kuopio, Finland	paivi.auvinen@kuh.fi	Clinical Groups	Oncology Group
Klaus Elenius	Hospital District of Southwest Finland, Turku, Finland	klaus.elenius@utu.fi	Clinical Groups	Oncology Group
Johanna Schleutker	Hospital District of Southwest Finland, Turku, Finland	johanna.schleutker@utu.fi	Clinical Groups	Oncology Group
Esa Pitkänen	Institute for Molecular Medicine Finland (FIMM), HiLIFE,	esa.pitkanen@helsinki.fi	Clinical Groups	Oncology Group

For peer review only

For peer review only - http://bmjopen.bmj.com/site/about/guidelines.xhtml

	University of Helsinki, Helsinki, Finland			
Nina Mars	Institute for Molecular Medicine Finland (FIMM), HiLIFE, University of Helsinki, Helsinki, Finland	nina.mars@helsinki.fi	Clinical Groups	Oncology Group
Mark Daly	Institute for Molecular Medicine Finland (FIMM), HiLIFE, University of Helsinki, Helsinki, Finland; Broad Institute of MIT and Harvard; Massachusetts General Hospital	mark.daly@helsinki.fi	Clinical Groups	Oncology Group
Relja Popovic	Abbvie, Chicago, IL, United States	relja.popovic@abbvie.com	Clinical Groups	Oncology Group
Jeffrey Waring	Abbvie, Chicago, IL, United States	jeff.waring@abbvie.com	Clinical Groups	Oncology Group
Bridget Riley-Gillis	Abbvie, Chicago, IL, United States	bridget.rileygillis@abbvie.com	Clinical Groups	Oncology Group
Anne Lehtonen	Abbvie, Chicago, IL, United States	anne.lehtonen@abbvie.com	Clinical Groups	Oncology Group
Jennifer Schutzman	Genentech, San Francisco, CA, United States	schutzman.jennifer@gene.com	Clinical Groups	Oncology Group

For peer review only - http://bmjopen.bmj.com/site/about/guidelines.xhtml

Julie Hunkapiller	Genentech, San Francisco, CA, United States	hunkapiller.julie@gene.com	Clinical Groups	Oncology Group
Natalie Bowers	Genentech, San Francisco, CA, United States	bowersn1@gene.com	Clinical Groups	Oncology Group
Rion Pendergrass	Genentech, San Francisco, CA, United States	penders2@gene.com	Clinical Groups	Oncology Group
Diptee Kulkarni	GlaxoSmithKline, Brentford, United Kingdom	diptee.a.kulkarni@gsk.com	Clinical Groups	Oncology Group
Kirsi Auro	GlaxoSmithKline, Espoo, Finland	kirsi.m.auro@gsk.com	Clinical Groups	Oncology Group
Alessandro Porello	Janssen Research & Development, LLC, Spring House, PA, United States	APorrell@ITS.JNJ.com	Clinical Groups	Oncology Group
Andrey Loboda	Merck, Kenilworth, NJ, United States	andrey_loboda@merck.com	Clinical Groups	Oncology Group
Heli Lehtonen	Pfizer, New York, NY, United States	heli.lehtonen@pfizer.com	Clinical Groups	Oncology Group
Stefan McDonough	Pfizer, New York, NY, United States	stefan.McDonough@pfizer.com	Clinical Groups	Oncology Group
Sauli Vuoti	Janssen-Cilag Oy, Espoo, Finland	svuoti@its.jnj.com	Clinical Groups	Oncology Group

46

Kai Kaarniranta	Northern Savo Hospital District, Kuopio, Finland	kai.kaarniranta@uef.fi	Clinical Groups	Opthalmology Group
Joni A Turunen	Helsinki University Hospital and University of Helsinki, Helsinki, Finland; Eye Genetics Group, Folkhälsan Research Center, Helsinki, Finland	joni.turunen@helsinki.fi	Clinical Groups	Opthalmology Group
Terhi Ollila	Hospital District of Helsinki and Uusimaa, Helsinki, Finland	terhi.ollila@hus.fi	Clinical Groups	Opthalmology Group
Hannu Uusitalo	Pirkanmaa Hospital District, Tampere, Finland	hannu.uusitalo@tuni.fi	Clinical Groups	Opthalmology Group
Juha Karjalainen	Institute for Molecular Medicine Finland (FIMM), HiLIFE, University of Helsinki, Helsinki, Finland	juha.karjalainen@helsinki.fi	Clinical Groups	Opthalmology Group
Esa Pitkänen	Institute for Molecular Medicine Finland (FIMM), HiLIFE, University of Helsinki, Helsinki, Finland	esa.pitkanen@helsinki.fi	Clinical Groups	Opthalmology Group
Mengzhen Liu	Abbvie, Chicago, IL, United States	mengzhen.liu@abbvie.com	Clinical Groups	Opthalmology Group
Heiko Runz	Biogen, Cambridge, MA, United States	heiko.runz@biogen.com	Clinical Groups	Opthalmology Group

For peer review only

Stephanie Loomis	Biogen, Cambridge, MA, United States	stephanie.loomis@biogen.com	Clinical Groups	Opthalmology Group
Erich Strauss	Genentech, San Francisco, CA, United States	strauss.erich@gene.com	Clinical Groups	Opthalmology Group
Natalie Bowers	Genentech, San Francisco, CA, United States	bowersn1@gene.com	Clinical Groups	Opthalmology Group
Hao Chen	Genentech, San Francisco, CA, United States	haoc@gene.com	Clinical Groups	Opthalmology Group
Rion Pendergrass	Genentech, San Francisco, CA, United States	penders2@gene.com	Clinical Groups	Opthalmology Group
Kaisa Tasanen	Wellbeing Services County of North Ostrobothnia, University of Oulu, Oulu, Finland	kaisa.tasanen-maatta@oulu.fi	Clinical Groups	Dermatology Group
Laura Huilaja	Wellbeing Services County of North Ostrobothnia, University of Oulu, Oulu, Finland	laura.huilaja@oulu.fi	Clinical Groups	Dermatology Group
Katariina Hannula-Jouppi	Hospital District of Helsinki and Uusimaa, Helsinki, Finland	katariina.hannula-jouppi@hus.fi	Clinical Groups	Dermatology Group
Teea Salmi	Pirkanmaa Hospital District, Tampere, Finland	teea.salmi@pshp.fi	Clinical Groups	Dermatology Group

For peer review only

Sirkku Peltonen	Hospital District of Southwest Finland, Turku, Finland	sipelto@utu.fi	Clinical Groups	Dermatology Group
Leena Koulu	Hospital District of Southwest Finland, Turku, Finland	leena.koulu@tyks.fi	Clinical Groups	Dermatology Group
Nizar Smaoui	Abbvie, Chicago, IL, United States	nizar.smaoui@abbvie.com	Clinical Groups	Dermatology Group
Fedik Rahimov	Abbvie, Chicago, IL, United States	fedik.rahimov@abbvie.com	Clinical Groups	Dermatology Group
Anne Lehtonen	Abbvie, Chicago, IL, United States	anne.lehtonen@abbvie.com	Clinical Groups	Dermatology Group
David Choy	Genentech, San Francisco, CA, United States	choy.david@gene.com	Clinical Groups	Dermatology Group
Rion Pendergrass	Genentech, San Francisco, CA, United States	penders2@gene.com	Clinical Groups	Dermatology Group
Dawn Waterworth	Janssen Research & Development, LLC, Spring House, PA, United States	dwaterwo@its.jnj.com	Clinical Groups	Dermatology Group
Kirsi Kalpala	Pfizer, New York, NY, United States	kirsi.kalpala@pfizer.com	Clinical Groups	Dermatology Group
Ying Wu	Pfizer, New York, NY, United States	ying.wu3@pfizer.com	Clinical Groups	Dermatology Group

For peer review only

Pirkko Pussinen	Hospital District of Helsinki and Uusimaa, Helsinki, Finland	pirkko.pussinen@helsinki.fi	Clinical Groups	Odontology Group
Aino Salminen	Hospital District of Helsinki and Uusimaa, Helsinki, Finland	aino.m.salminen@helsinki.fi	Clinical Groups	Odontology Group
Tuula Salo	Hospital District of Helsinki and Uusimaa, Helsinki, Finland	tuula.salo@helsinki.fi	Clinical Groups	Odontology Group
David Rice	Hospital District of Helsinki and Uusimaa, Helsinki, Finland	david.rice@helsinki.fi	Clinical Groups	Odontology Group
Pekka Nieminen	Hospital District of Helsinki and Uusimaa, Helsinki, Finland	pekka.nieminen@helsinki.fi	Clinical Groups	Odontology Group
Ulla Palotie	Hospital District of Helsinki and Uusimaa, Helsinki, Finland	ulla.palotie@helsinki.fi	Clinical Groups	Odontology Group
Maria Siponen	Northern Savo Hospital District, Kuopio, Finland	maria.siponen@uef.fi	Clinical Groups	Odontology Group
Liisa Suominen	Northern Savo Hospital District, Kuopio, Finland	liisa.suominen@uef.fi	Clinical Groups	Odontology Group
Päivi Mäntylä	Northern Savo Hospital District, Kuopio, Finland	paivi.mantyla@uef.fi	Clinical Groups	Odontology Group
Ulvi Gursoy	Hospital District of Southwest Finland, Turku, Finland	ulvi.gursoy@utu.fi	Clinical Groups	Odontology Group

For peer review only - http://bmjopen.bmj.com/site/about/guidelines.xhtml

Vuokko Anttonen	Wellbeing Services County of North Ostrobothnia, University of Oulu, Oulu, Finland	vuokko.anttonen@oulu.fi	Clinical Groups	Odontology Group
Kirsi Sipilä	Research Unit of Oral Health Sciences Faculty of Medicine, University of Oulu, Oulu, Finland; Medical Research Center, Oulu, Oulu University Hospital and University of Oulu, Oulu, Finland	kirsi.sipila@oulu.fi	Clinical Groups	Odontology Group
Rion Pendergrass	Genentech, San Francisco, CA, United States	pendergrass.sarah@gene.com	Clinical Groups	Odontology Group
Hannele Laivuori	Institute for Molecular Medicine Finland (FIMM), HiLIFE, University of Helsinki, Helsinki, Finland	hannele.laivuori@helsinki.fi	Clinical Groups	Women's Health and Reproduction Group
Venla Kurra	Pirkanmaa Hospital District, Tampere, Finland	venla.kurra@tuni.fi	Clinical Groups	Women's Health and Reproduction Group
Laura Kotaniemi-Talonen	Pirkanmaa Hospital District, Tampere, Finland	laura.kotaniemi-talonen@tuni.fi	Clinical Groups	Women's Health and Reproduction Group

For peer review only

Oskari Heikinheimo	Hospital District of Helsinki and Uusimaa, Helsinki, Finland	oskari.heikinheimo@helsinki.fi	Clinical Groups	Women's Health and Reproduction Group
Ilkka Kalliala	Hospital District of Helsinki and Uusimaa, Helsinki, Finland	ilkka.kalliala@hus.fi	Clinical Groups	Women's Health and Reproduction Group
Lauri Aaltonen	Hospital District of Helsinki and Uusimaa, Helsinki, Finland	lauri.aaltonen@helsinki.fi	Clinical Groups	Women's Health and Reproduction Group
Varpu Jokimaa	Hospital District of Southwest Finland, Turku, Finland	varpu.jokimaa@utu.fi	Clinical Groups	Women's Health and Reproduction Group
Johannes Kettunen	Wellbeing Services County of North Ostrobothnia, Oulu, Finland	Johannes.Kettunen@oulu.fi	Clinical Groups	Women's Health and Reproduction Group
Marja Vääräsmäki	Wellbeing Services County of North Ostrobothnia, Oulu, Finland	marja.vaarasmaki@oulu.fi	Clinical Groups	Women's Health and

				Reproduction Group
Outi Uimari	University of Oulu, Wellbeing Services County of North Ostrobothnia, Oulu, Finland	outi.uimari@oulu.fi	Clinical Groups	Women's Health and Reproduction Group
Laure Morin-Papunen	Wellbeing Services County of North Ostrobothnia, Oulu, Finland	lmp@cc.oulu.fi	Clinical Groups	Women's Health and Reproduction Group
Maarit Niinimäki	Wellbeing Services County of North Ostrobothnia, Oulu, Finland	maarit.niinimaki@oulu.fi	Clinical Groups	Women's Health and Reproduction Group
Terhi Piltonen	University of Oulu, Wellbeing Services County of North Ostrobothnia, Oulu, Finland	terhi.piltonen@oulu.fi	Clinical Groups	Women's Health and Reproduction Group
Katja Kivinen	Institute for Molecular Medicine Finland (FIMM), HiLIFE, University of Helsinki, Helsinki, Finland	katja.kivinen@helsinki.fi	Clinical Groups	Women's Health and Reproduction Group

For peer review only

Elisabeth Widen	Institute for Molecular Medicine Finland (FIMM), HiLIFE, University of Helsinki, Helsinki, Finland	elisabeth.widen@helsinki.fi	Clinical Groups	Women's Health and Reproduction Group
Taru Tukiainen	Institute for Molecular Medicine Finland (FIMM), HiLIFE, University of Helsinki, Helsinki, Finland	taru.tukiainen@helsinki.fi	Clinical Groups	Women's Health and Reproduction Group
Mary Pat Reeve	Institute for Molecular Medicine Finland (FIMM), HiLIFE, University of Helsinki, Helsinki, Finland	mary.reeve@helsinki.fi	Clinical Groups	Women's Health and Reproduction Group
Mark Daly	Institute for Molecular Medicine Finland (FIMM), HiLIFE, University of Helsinki, Helsinki, Finland; Broad Institute of MIT and Harvard; Massachusetts General Hospital	mark.daly@helsinki.fi	Clinical Groups	Women's Health and Reproduction Group
Niko Välimäki	University of Helsinki, Helsinki, Finland	niko.valimaki@helsinki.fi	Clinical Groups	Women's Health and Reproduction Group

For peer review only

For peer review only - http://bmjopen.bmj.com/site/about/guidelines.xhtml

Eija Laakkonen	University of Jyväskylä, Jyväskylä, Finland	eija.k.laakkonen@jyu.fi	Clinical Groups	Women's Health and Reproduction Group
Jaakko Tyrmi	University of Oulu, Oulu, Finland / University of Tampere, Tampere, Finland	jaakko.tyrmi@oulu.fi	Clinical Groups	Women's Health and Reproduction Group
Heidi Silven	University of Oulu, Oulu, Finland	heidi.silven@student.oulu.fi	Clinical Groups	Women's Health and Reproduction Group
Eeva Sliz	University of Oulu, Oulu, Finland	eeva.sliz@oulu.fi	Clinical Groups	Women's Health and Reproduction Group
Riikka Arffman	University of Oulu, Oulu, Finland	riikka.arffman@oulu.fi	Clinical Groups	Women's Health and Reproduction Group
Susanna Savukoski	University of Oulu, Oulu, Finland	susanna.savukoski@oulu.fi	Clinical Groups	Women's Health and

For peer review only

				Reproduction Group
Triin Laisk	Estonian biobank, Tartu, Estonia	triin.laisk@ut.ee	Clinical Groups	Women's Health and Reproduction Group
Natalia Pujol	Estonian biobank, Tartu, Estonia	natalia.pujolgualdo@oulu.fi	Clinical Groups	Women's Health and Reproduction Group
Mengzhen Liu	Abbvie, Chicago, IL, United States	mengzhen.liu@abbvie.com	Clinical Groups	Women's Health and Reproduction Group
Bridget Riley-Gillis	Abbvie, Chicago, IL, United States	bridget.rileygillis@abbvie.com	Clinical Groups	Women's Health and Reproduction Group
Rion Pendergrass	Genentech, San Francisco, CA, United States	penders2@gene.com	Clinical Groups	Women's Health and Reproduction Group

56

For peer review only

Janet Kumar	GlaxoSmithKline, Collegeville, PA, United States	janet.x.kumar@gsk.com	Clinical Groups	Women's Health and Reproduction Group
Kirsi Auro	GlaxoSmithKline, Espoo, Finland	kirsi.m.auro@gsk.com	Clinical Groups	Women's Health and Reproduction Group
Iiris Hovatta	University of Helsinki, Finland	iiris.hovatta@helsinki.fi	Clinical Groups	Depression group
Chia-Yen Chen	Biogen, Cambridge, MA, United States	chiayen.chen@biogen.com	Clinical Groups	Depression group
Erkki Isometsä	Hospital District of Helsinki and Uusimaa, Helsinki, Finland	erkki.isometsa@hus.fi	Clinical Groups	Depression group
Hanna Ollila	Institute for Molecular Medicine Finland (FIMM), HiLIFE, University of Helsinki, Helsinki, Finland	hanna.m.ollila@helsinki.fi	Clinical Groups	Depression group
Jaana Suvisaari	Finnish Institute for Health and Welfare (THL), Helsinki, Finland	jaana.suvisaari@thl.fi	Clinical Groups	Depression group
Thomas Damm Als	Aarhus University, Denmark	tda@biomed.au.dk	Clinical Groups	Depression group

For peer review only

Antti Mäkitie	Department of Otorhinolaryngology - Head and Neck Surgery, University of Helsinki and Helsinki University Hospital, Helsinki, Finland	antti.makitie@helsinki.fi	Clinical Groups	ENT (ear, nose and throat) Group
Argyro Bizaki-Vallaskangas	Pirkanmaa Hospital District, Tampere, Finland	argyro.bizaki-vallaskangas@tuni.fi	Clinical Groups	ENT (ear, nose and throat) Group
Sanna Toppila-Salmi	University of Helsinki, Finland	sanna.salmi@helsinki.fi	Clinical Groups	ENT (ear, nose and throat) Group
Tytti Willberg	Hospital District of Southwest Finland, Turku, Finland	tytti.willberg@tyks.fi	Clinical Groups	ENT (ear, nose and throat) Group
Elmo Saarentaus	Institute for Molecular Medicine Finland (FIMM), HiLIFE, University of Helsinki, Helsinki, Finland	elmo.saarentaus@helsinki.fi	Clinical Groups	ENT (ear, nose and throat) Group
Antti Aarnisalo	Hospital District of Helsinki and Uusimaa, Helsinki, Finland	antti.aarnisalo@hus.fi	Clinical Groups	ENT (ear, nose and throat) Group

For peer review only - http://bmjopen.bmj.com/site/about/guidelines.xhtml

Eveliina Salminen	Hospital District of Helsinki and Uusimaa, Helsinki, Finland	eveliina.e.salminen@hus.fi	Clinical Groups	ENT (ear, nose and throat) Group
Elisa Rahikkala	Wellbeing Services County of North Ostrobothnia, Oulu, Finland	elisa.rahikkala@ppshp.fi	Clinical Groups	ENT (ear, nose and throat) Group
Johannes Kettunen	University of Oulu, Wellbeing Services County of North Ostrobothnia, Oulu, Finland	johannes.kettunen@oulu.fi	Clinical Groups	ENT (ear, nose and throat) Group
Kristiina Aittomäki	Department of Medical Genetics, Helsinki University Central Hospital, Helsinki, Finland	kristiina.aittomaki@helsinki.fi	Clinical Groups	POI (premature ovarian failure) Group
Fredrik Åberg	Transplantation and Liver Surgery Clinic, Helsinki University Hospital, Helsinki University, Helsinki, Finland	fredrik.aberg@helsinki.fi	Clinical Groups	LiverScore Group
Mitja Kurki	Institute for Molecular Medicine Finland (FIMM), HiLIFE, University of Helsinki, Helsinki, Finland; Broad Institute, Cambridge, MA, United States	mkurki@broadinstitute.org	FinnGen Analysis working group	FinnGen Analysis working group

For peer review only

Samuli Ripatti	Institute for Molecular Medicine Finland (FIMM), HiLIFE, University of Helsinki, Helsinki, Finland	samuli.ripatti@helsinki.fi	FinnGen Analysis working group	FinnGen Analysis working group
Mark Daly	Institute for Molecular Medicine, Finland (FIMM), HiLIFE, University of Helsinki, Helsinki, Finland; Broad Institute of MIT and Harvard; Massachusetts General Hospital	mark.daly@helsinki.fi	FinnGen Analysis working group	FinnGen Analysis working group
Juha Karjalainen	Institute for Molecular Medicine Finland (FIMM), HiLIFE, University of Helsinki, Helsinki, Finland	juha.karjalainen@helsinki.fi	FinnGen Analysis working group	FinnGen Analysis working group
Aki Havulinna	Institute for Molecular Medicine Finland (FIMM), HiLIFE, University of Helsinki, Helsinki, Finland; Finnish Institute for Health and Welfare (THL), Helsinki, Finland	aki.havulinna@helsinki.fi	FinnGen Analysis working group	FinnGen Analysis working group
Juha Mehtonen	Institute for Molecular Medicine Finland (FIMM), HiLIFE,	juha.mehtonen@helsinki.fi	FinnGen Analysis working group	FinnGen Analysis working group

For peer review only - http://bmjopen.bmj.com/site/about/guidelines.xhtml

	University of Helsinki, Helsinki, Finland			
Priit Palta	Institute for Molecular Medicine Finland (FIMM), HiLIFE, University of Helsinki, Helsinki, Finland	priit.palta@helsinki.fi	FinnGen Analysis working group	FinnGen Analysis working group
Shabbeer Hassan	Institute for Molecular Medicine Finland (FIMM), HiLIFE, University of Helsinki, Helsinki, Finland	shabbeer.hassan@helsinki.fi	FinnGen Analysis working group	FinnGen Analysis working group
Pietro Della Briotta Parolo	Institute for Molecular Medicine Finland (FIMM), HiLIFE, University of Helsinki, Helsinki, Finland	pietro.dellabriottaparolo@helsinki.fi	FinnGen Analysis working group	FinnGen Analysis working group
Wei Zhou	Broad Institute, Cambridge, MA, United States	wzhou@broadinstitute.org	FinnGen Analysis working group	FinnGen Analysis working group
Mutaamba Maasha	Broad Institute, Cambridge, MA, United States	mmaasha@broadinstitute.org	FinnGen Analysis working group	FinnGen Analysis working group
Shabbeer Hassan	Institute for Molecular Medicine Finland (FIMM), HiLIFE,	shabbeer.hassan@helsinki.fi	FinnGen Analysis working group	FinnGen Analysis working group

61

For peer review only

	University of Helsinki, Helsinki, Finland			
Susanna Lemmelä	Institute for Molecular Medicine Finland (FIMM), HiLIFE, University of Helsinki, Helsinki, Finland	susanna.lemmela@helsinki.fi	FinnGen Analysis working group	FinnGen Analysis working group
Manuel Rivas	University of Stanford, Stanford, CA, United States	mrivas@stanford.edu	FinnGen Analysis working group	FinnGen Analysis working group
Mari E. Niemi	Institute for Molecular Medicine Finland (FIMM), HiLIFE, University of Helsinki, Helsinki, Finland	mari.e.niemi@helsinki.fi	FinnGen Analysis working group	FinnGen Analysis working group
Aarno Palotie	Institute for Molecular Medicine Finland (FIMM), HiLIFE, University of Helsinki, Helsinki, Finland	aarno.palotie@helsinki.fi	FinnGen Analysis working group	FinnGen Analysis working group
Aoxing Liu	Institute for Molecular Medicine Finland (FIMM), HiLIFE, University of Helsinki, Helsinki, Finland	aoxing.liu@helsinki.fi	FinnGen Analysis working group	FinnGen Analysis working group

For peer review only - http://bmjopen.bmj.com/site/about/guidelines.xhtml

Arto Lehisto	Institute for Molecular Medicine Finland (FIMM), HiLIFE, University of Helsinki, Helsinki, Finland	arto.lehisto@helsinki.fi	FinnGen Analysis working group	FinnGen Analysis working group
Andrea Ganna	Institute for Molecular Medicine Finland (FIMM), HiLIFE, University of Helsinki, Helsinki, Finland	aganna@broadinstitute.org	FinnGen Analysis working group	FinnGen Analysis working group
Vincent Llorens	Institute for Molecular Medicine Finland (FIMM), HiLIFE, University of Helsinki, Helsinki, Finland	vincent.llorens@helsinki.fi	FinnGen Analysis working group	FinnGen Analysis working group
Hannele Laivuori	Institute for Molecular Medicine Finland (FIMM), HiLIFE, University of Helsinki, Helsinki, Finland	hannele.laivuori@helsinki.fi	FinnGen Analysis working group	FinnGen Analysis working group
Taru Tukiainen	Institute for Molecular Medicine Finland (FIMM), HiLIFE, University of Helsinki, Helsinki, Finland	taru.tukiainen@helsinki.fi	FinnGen Analysis working group	FinnGen Analysis working group
Mary Pat Reeve	Institute for Molecular Medicine Finland (FIMM), HiLIFE,	mary.reeve@helsinki.fi	FinnGen Analysis working group	FinnGen Analysis working group

For peer review only

	University of Helsinki, Helsinki, Finland			
Henrike Heyne	Institute for Molecular Medicine Finland (FIMM), HiLIFE, University of Helsinki, Helsinki, Finland	hheyne@broadinstitute.org	FinnGen Analysis working group	FinnGen Analysis working group
Nina Mars	Institute for Molecular Medicine Finland (FIMM), HiLIFE, University of Helsinki, Helsinki, Finland	nina.mars@helsinki.fi	FinnGen Analysis working group	FinnGen Analysis working group
Joel Rämö	Institute for Molecular Medicine Finland (FIMM), HiLIFE, University of Helsinki, Helsinki, Finland	joel.ramo@helsinki.fi	FinnGen Analysis working group	FinnGen Analysis working group
Elmo Saarentaus	Institute for Molecular Medicine Finland (FIMM), HiLIFE, University of Helsinki, Helsinki, Finland	elmo.saarentaus@helsinki.fi	FinnGen Analysis working group	FinnGen Analysis working group
Hanna Ollila	Institute for Molecular Medicine Finland (FIMM), HiLIFE, University of Helsinki, Helsinki, Finland	hanna.m.ollila@helsinki.fi	FinnGen Analysis working group	FinnGen Analysis working group

For peer review only - http://bmjopen.bmj.com/site/about/guidelines.xhtml

Rodos Rodosthenous	Institute for Molecular Medicine Finland (FIMM), HiLIFE, University of Helsinki, Helsinki, Finland	rodos.rodosthenous@helsinki.fi	FinnGen Analysis working group	FinnGen Analysis working group
Satu Strausz	Institute for Molecular Medicine Finland (FIMM), HiLIFE, University of Helsinki, Helsinki, Finland	satu.strausz@helsinki.fi	FinnGen Analysis working group	FinnGen Analysis working group
Tuula Palotie	University of Helsinki and Hospital District of Helsinki and Uusimaa, Helsinki, Finland	tuula.palotie@helsinki.fi	FinnGen Analysis working group	FinnGen Analysis working group
Kimmo Palin	University of Helsinki, Helsinki, Finland	kimmo.palin@helsinki.fi	FinnGen Analysis working group	FinnGen Analysis working group
Javier Garcia-Tabuenca	University of Tampere, Tampere, Finland	javier.graciatabuenca@tuni.fi	FinnGen Analysis working group	FinnGen Analysis working group
Harri Siirtola	University of Tampere, Tampere, Finland	harri.siirtola@tuni.fi	FinnGen Analysis working group	FinnGen Analysis working group
Tuomo Kiiskinen	Institute for Molecular Medicine Finland (FIMM), HiLIFE,	tuomo.kiiskinen@helsinki.fi	FinnGen Analysis working group	FinnGen Analysis working group

For peer review only

	University of Helsinki, Helsinki, Finland			
Jiwoo Lee	Institute for Molecular Medicine Finland (FIMM), HiLIFE, University of Helsinki, Helsinki, Finland; Broad Institute, Cambridge, MA, United States	jiwoo.lee@helsinki.fi	FinnGen Analysis working group	FinnGen Analysis working group
Kristin Tsuo	Institute for Molecular Medicine Finland (FIMM), HiLIFE, University of Helsinki, Helsinki, Finland; Broad Institute, Cambridge, MA, United States	kristintsuo@fas.harvard.edu	FinnGen Analysis working group	FinnGen Analysis working group
Amanda Elliott	Institute for Molecular Medicine Finland (FIMM), HiLIFE, University of Helsinki, Helsinki, Finland; Broad Institute, Cambridge, MA, USA and Massachusetts General Hospital, Boston, MA, USA	aelliott@broadinstitute.org	FinnGen Analysis working group	FinnGen Analysis working group
Kati Kristiansson	THL Biobank / Finnish Institute for Health and Welfare (THL), Helsinki, Finland	kati.kristiansson@thl.fi	FinnGen Analysis working group	FinnGen Analysis working group

For peer review only - http://bmjopen.bmj.com/site/about/guidelines.xhtml

Mikko Arvas	Finnish Red Cross Blood Service / Finnish Hematology Registry and Clinical Biobank, Helsinki, Finland	mikko.arvas@veripalvelu.fi	FinnGen Analysis working group	FinnGen Analysis working group
Kati Hyvärinen	Finnish Red Cross Blood Service, Helsinki, Finland	kati.hyvarinen@veripalvelu.fi	FinnGen Analysis working group	FinnGen Analysis working group
Jarmo Ritari	Finnish Red Cross Blood Service, Helsinki, Finland	jarmo.ritari@veripalvelu.fi	FinnGen Analysis working group	FinnGen Analysis working group
Olli Carpén	Helsinki Biobank / Helsinki University and Hospital District of Helsinki and Uusimaa, Helsinki	olli.carpen@helsinki.fi	FinnGen Analysis working group	FinnGen Analysis working group
Johannes Kettunen	Northern Finland Biobank Borealis / University of Oulu / Wellbeing Services County of North Ostrobothnia, Oulu, Finland	johannes.kettunen@oulu.fi	FinnGen Analysis working group	FinnGen Analysis working group
Katri Pylkäs	University of Oulu, Oulu, Finland	katri.pylkas@oulu.fi	FinnGen Analysis working group	FinnGen Analysis working group
Eeva Sliz	University of Oulu, Oulu, Finland	eeva.sliz@oulu.fi	FinnGen Analysis working group	FinnGen Analysis working group

67

For peer review only

Minna Karjalainen	University of Oulu, Oulu, Finland	minna.k.karjalainen@oulu.fi	FinnGen Analysis working group	FinnGen Analysis working group
Tuomo Mantere	Northern Finland Biobank Borealis / University of Oulu / Wellbeing Services County of North Ostrobothnia, Oulu, Finland	tuomo.mantere@oulu.fi	FinnGen Analysis working group	FinnGen Analysis working group
Eeva Kangasniemi	Finnish Clinical Biobank Tampere / University of Tampere / The Wellbeing Services County of Pirkanmaa, Tampere, Finland	eeva.kangasniemi@pirha.fi	FinnGen Analysis working group	FinnGen Analysis working group
Sami Heikkinen	University of Eastern Finland, Kuopio, Finland	sami.heikkinen@uef.fi	FinnGen Analysis working group	FinnGen Analysis working group
Arto Mannermaa	Biobank of Eastern Finland / University of Eastern Finland / Northern Savo Hospital District, Kuopio, Finland	arto.mannermaa@uef.fi	FinnGen Analysis working group	FinnGen Analysis working group
Eija Laakkonen	University of Jyväskylä, Jyväskylä, Finland	eija.k.laakkonen@jyu.fi	FinnGen Analysis working group	FinnGen Analysis working group

For peer review only - http://bmjopen.bmj.com/site/about/guidelines.xhtml

Nina Pitkänen	Auria Biobank / University of Turku / Hospital District of Southwest Finland, Turku, Finland	Niina.Pitkanen@tyks.fi	FinnGen Analysis working group	FinnGen Analysis working group
Samuel Lessard	Translational Sciences, Sanofi R&D, Framingham, MA, USA	samuel.lessard@sanofi.com	FinnGen Analysis working group	FinnGen Analysis working group
Clément Chatelain	Translational Sciences, Sanofi R&D, Framingham, MA, USA	clement.chatelain@sanofi.com	FinnGen Analysis working group	FinnGen Analysis working group
Perttu Terho	Auria Biobank / University of Turku / Hospital District of Southwest Finland, Turku, Finland	perttu.terho@tyks.fi	Biobank directors	Biobank directors
Sirpa Soini	THL Biobank / Finnish Institute for Health and Welfare (THL), Helsinki, Finland	sirpa.soini@thl.fi	Biobank directors	Biobank directors
Jukka Partanen	Finnish Red Cross Blood Service / Finnish Hematology Registry and Clinical Biobank, Helsinki, Finland	jukka.partanen@veripalvelu.fi	Biobank directors	Biobank directors
Eero Punkka	Helsinki Biobank / Helsinki University and Hospital District of Helsinki and Uusimaa, Helsinki	eero.punkka@hus.fi	Biobank directors	Biobank directors
Raisa Serpi	Northern Finland Biobank Borealis / University of Oulu / Wellbeing	raisa.serpi@pohde.fi	Biobank directors	Biobank directors

For peer review only

	Services County of North Ostrobothnia, Oulu, Finland			
Sanna Siltanen	Finnish Clinical Biobank Tampere / University of Tampere / The Wellbeing Services County of Pirkanmaa, Tampere, Finland	sanna.siltanen@pirha.fi	Biobank directors	Biobank directors
Veli-Matti Kosma	Biobank of Eastern Finland / University of Eastern Finland / Northern Savo Hospital District, Kuopio, Finland	veli-matti.kosma@uef.fi	Biobank directors	Biobank directors
Teijo Kuopio	Central Finland Biobank / University of Jyväskylä / Central Finland Health Care District, Jyväskylä, Finland	teijo.kuopio@ksshp.fi	Biobank directors	Biobank directors
Anu Jalanko	Institute for Molecular Medicine Finland (FIMM), HiLIFE, University of Helsinki, Helsinki, Finland	anu.jalanko@helsinki.fi	FinnGen Teams	Administration
Huei-Yi Shen	Institute for Molecular Medicine Finland (FIMM), HiLIFE, University of Helsinki, Helsinki, Finland	huei-yi.shen@helsinki.fi	FinnGen Teams	Administration

For peer review only
For peer review only - http://bmjopen.bmj.com/site/about/guidelines.xhtml

Risto Kajanne	Institute for Molecular Medicine Finland (FIMM), HiLIFE, University of Helsinki, Helsinki, Finland	risto.kajanne@helsinki.fi	FinnGen Teams	Administration
Mervi Aavikko	Institute for Molecular Medicine Finland (FIMM), HiLIFE, University of Helsinki, Helsinki, Finland	mervi.aavikko@helsinki.fi	FinnGen Teams	Administration
Henna Palin	Finnish Clinical Biobank Tampere / University of Tampere / The Wellbeing Services County of Pirkanmaa, Tampere, Finland	henna.palin@pirha.fi	FinnGen Teams	Administration
Malla-Maria Linna	Helsinki Biobank / Helsinki University and Hospital District of Helsinki and Uusimaa, Helsinki	malla-maria.linna@hus.fi	FinnGen Teams	Administration
Mitja Kurki	Institute for Molecular Medicine Finland (FIMM), HiLIFE, University of Helsinki, Helsinki, Finland; Broad Institute, Cambridge, MA, United States	mkurki@broadinstitute.org	FinnGen Teams	Analysis
Juha Karjalainen	Institute for Molecular Medicine Finland (FIMM), HiLIFE,	juha.karjalainen@helsinki.fi	FinnGen Teams	Analysis

For peer review only - http://bmjopen.bmj.com/site/about/guidelines.xhtml

	University of Helsinki, Helsinki, Finland			
Pietro Della Briotta Parolo	Institute for Molecular Medicine Finland (FIMM), HiLIFE, University of Helsinki, Helsinki, Finland	pietro.dellabriottaparolo@helsinki.fi	FinnGen Teams	Analysis
Arto Lehisto	Institute for Molecular Medicine Finland (FIMM), HiLIFE, University of Helsinki, Helsinki, Finland	arto.lehisto@helsinki.fi	FinnGen Teams	Analysis
Juha Mehtonen	Institute for Molecular Medicine Finland (FIMM), HiLIFE, University of Helsinki, Helsinki, Finland	juha.mehtonen@helsinki.fi	FinnGen Teams	Analysis
Wei Zhou	Broad Institute, Cambridge, MA, United States	wzhou@broadinstitute.org	FinnGen Teams	Analysis
Masahiro Kanai	Broad Institute, Cambridge, MA, United States	mkanai@broadinstitute.org	FinnGen Teams	Analysis
Mutaamba Maasha	Broad Institute, Cambridge, MA, United States	mmaasha@broadinstitute.org	FinnGen Teams	Analysis
Hannele Laivuori	Institute for Molecular Medicine Finland (FIMM), HiLIFE,	hannele.laivuori@helsinki.fi	FinnGen Teams	Clinical Endpoint Development

For peer review only

	University of Helsinki, Helsinki, Finland			
Aki Havulinna	Institute for Molecular Medicine Finland (FIMM), HiLIFE, University of Helsinki, Helsinki, Finland; Finnish Institute for Health and Welfare (THL), Helsinki, Finland	aki.havulinna@helsinki.fi	FinnGen Teams	Clinical Endpoint Development
Susanna Lemmelä	Institute for Molecular Medicine Finland (FIMM), HiLIFE, University of Helsinki, Helsinki, Finland	susanna.lemmela@helsinki.fi	FinnGen Teams	Clinical Endpoint Development
Tuomo Kiiskinen	Institute for Molecular Medicine Finland (FIMM), HiLIFE, University of Helsinki, Helsinki, Finland	tuomo.kiiskinen@helsinki.fi	FinnGen Teams	Clinical Endpoint Development
L. Elisa Lahtela	Institute for Molecular Medicine Finland (FIMM), HiLIFE, University of Helsinki, Helsinki, Finland	laura.lahtela@helsinki.fi	FinnGen Teams	Clinical Endpoint Development
Mari Kaunisto	Institute for Molecular Medicine Finland (FIMM), HiLIFE,	mari.kaunisto@helsinki.fi	FinnGen Teams	Communication

For peer review only - http://bmjopen.bmj.com/site/about/guidelines.xhtml

	University of Helsinki, Helsinki, Finland			
Elina Kilpeläinen	Institute for Molecular Medicine Finland (FIMM), HiLIFE, University of Helsinki, Helsinki, Finland	elina.kilpelainen@helsinki.fi	FinnGen Teams	E-Science
Timo P. Sipilä	Institute for Molecular Medicine Finland (FIMM), HiLIFE, University of Helsinki, Helsinki, Finland	timo.p.sipila@helsinki.fi	FinnGen Teams	E-Science
Oluwaseun Alexander Dada	Institute for Molecular Medicine Finland (FIMM), HiLIFE, University of Helsinki, Helsinki, Finland	alexander.dada@helsinki.fi	FinnGen Teams	E-Science
Awaisa Ghazal	Institute for Molecular Medicine Finland (FIMM), HiLIFE, University of Helsinki, Helsinki, Finland	awaisa.ghazal@helsinki.fi	FinnGen Teams	E-Science
Anastasia Kytölä	Institute for Molecular Medicine Finland (FIMM), HiLIFE, University of Helsinki, Helsinki, Finland	anastasia.shcherban@helsinki.fi	FinnGen Teams	E-Science

For peer review only

Rigbe Weldatsadik	Institute for Molecular Medicine Finland (FIMM), HiLIFE, University of Helsinki, Helsinki, Finland	rigbe.weldatsadik@helsinki.fi	FinnGen Teams	E-Science
Sanni Ruotsalainen	Institute for Molecular Medicine Finland (FIMM), HiLIFE, University of Helsinki, Helsinki, Finland	sanni.ruotsalainen@helsinki.fi	FinnGen Teams	E-Science
Kati Donner	Institute for Molecular Medicine Finland (FIMM), HiLIFE, University of Helsinki, Helsinki, Finland	kati.donner@helsinki.fi	FinnGen Teams	Genotyping
Timo P. Sipilä	Institute for Molecular Medicine Finland (FIMM), HiLIFE, University of Helsinki, Helsinki, Finland	timo.p.sipila@helsinki.fi	FinnGen Teams	Genotyping
Anu Loukola	Helsinki Biobank / Helsinki University and Hospital District of Helsinki and Uusimaa, Helsinki	anu.loukola@hus.fi	FinnGen Teams	Sample Collection Coordination
Päivi Laiho	THL Biobank / Finnish Institute for Health and Welfare (THL), Helsinki, Finland	paivi.laiho@thl.fi	FinnGen Teams	Sample Logistics

For peer review only

For peer review only - http://bmjopen.bmj.com/site/about/guidelines.xhtml

Tuuli Sistonen	THL Biobank / Finnish Institute for Health and Welfare (THL), Helsinki, Finland	tuuli.sistonen@thl.fi	FinnGen Teams	Sample Logistics
Essi Kaiharju	THL Biobank / Finnish Institute for Health and Welfare (THL), Helsinki, Finland	essi.kaiharju@thl.fi	FinnGen Teams	Sample Logistics
Markku Laukkanen	THL Biobank / Finnish Institute for Health and Welfare (THL), Helsinki, Finland	markku.laukkanen @thl.fi	FinnGen Teams	Sample Logistics
Elina Järvensivu	THL Biobank / Finnish Institute for Health and Welfare (THL), Helsinki, Finland	elina.jarvensivu@thl .fi	FinnGen Teams	Sample Logistics
Sini Lähteenmäki	THL Biobank / Finnish Institute for Health and Welfare (THL), Helsinki, Finland	sini.lahteenmaki@th l.fi	FinnGen Teams	Sample Logistics
Lotta Männikkö	THL Biobank / Finnish Institute for Health and Welfare (THL), Helsinki, Finland	lotta.mannikko@thl. fi	FinnGen Teams	Sample Logistics
Regis Wong	THL Biobank / Finnish Institute for Health and Welfare (THL), Helsinki, Finland	regis.wong@thl.fi	FinnGen Teams	Sample Logistics

For peer review only

For peer review only - http://bmjopen.bmj.com/site/about/guidelines.xhtml

Auli Toivola	THL Biobank / Finnish Institute for Health and Welfare (THL), Helsinki, Finland	auli.toivola@thl.fi	FinnGen Teams	Sample Logistics
Minna Brunfeldt	THL Biobank / Finnish Institute for Health and Welfare (THL), Helsinki, Finland	minna.brunfeldt@thl.fi	FinnGen Teams	Registry Data Operations
Hannele Mattsson	THL Biobank / Finnish Institute for Health and Welfare (THL), Helsinki, Finland	hannele.mattsson@thl.fi	FinnGen Teams	Registry Data Operations
Kati Kristiansson	THL Biobank / Finnish Institute for Health and Welfare (THL), Helsinki, Finland	kati.kristiansson@thl.fi	FinnGen Teams	Registry Data Operations
Susanna Lemmelä	Institute for Molecular Medicine Finland (FIMM), HiLIFE, University of Helsinki, Helsinki, Finland	susanna.lemmela@helsinki.fi	FinnGen Teams	Registry Data Operations
Sami Koskelainen	THL Biobank / Finnish Institute for Health and Welfare (THL), Helsinki, Finland	sami.koskelainen@thl.fi	FinnGen Teams	Registry Data Operations
Tero Hiekkalinna	THL Biobank / Finnish Institute for Health and Welfare (THL), Helsinki, Finland	tero.hiekkalinna@helsinki.fi	FinnGen Teams	Registry Data Operations

For peer review only - http://bmjopen.bmj.com/site/about/guidelines.xhtml

Teemu Paajanen	THL Biobank / Finnish Institute for Health and Welfare (THL), Helsinki, Finland	teemu.paajanen@thl.fi	FinnGen Teams	Registry Data Operations
Priit Palta	Institute for Molecular Medicine Finland (FIMM), HiLIFE, University of Helsinki, Helsinki, Finland	priit.palta@helsinki.fi	FinnGen Teams	Sequencing Informatics
Kalle Pärn	Institute for Molecular Medicine Finland (FIMM), HiLIFE, University of Helsinki, Helsinki, Finland	kalle.parn@helsinki.fi	FinnGen Teams	Sequencing Informatics
Mart Kals	Institute for Molecular Medicine Finland (FIMM), HiLIFE, University of Helsinki, Helsinki, Finland	mart.kals@helsinki.fi	FinnGen Teams	Sequencing Informatics
Shuang Luo	Institute for Molecular Medicine Finland (FIMM), HiLIFE, University of Helsinki, Helsinki, Finland	shuang.luo@helsinki.fi	FinnGen Teams	Sequencing Informatics
Tarja Laitinen	The Wellbeing Services County of Pirkanmaa, Tampere, Finland	tarja.laitinen@pirha.fi	FinnGen Teams	Trajectory
Mary Pat Reeve	Institute for Molecular Medicine Finland (FIMM), HiLIFE,	mary.reeve@helsinki.fi	FinnGen Teams	Trajectory

For peer review only

	University of Helsinki, Helsinki, Finland			
Shanmukha Sampath Padmanabhuni	Institute for Molecular Medicine Finland (FIMM), HiLIFE, University of Helsinki, Helsinki, Finland	sam.padmanabhuni@helsinki.fi	FinnGen Teams	Trajectory
Marianna Niemi	University of Tampere, Tampere, Finland	marianna.niemi@tuni.fi	FinnGen Teams	Trajectory
Harri Siirtola	University of Tampere, Tampere, Finland	harri.siirtola@tuni.fi	FinnGen Teams	Trajectory
Javier Gracia-Tabuenca	University of Tampere, Tampere, Finland	javier.graciatabuenca@tuni.fi	FinnGen Teams	Trajectory
Mika Helminen	University of Tampere, Tampere, Finland	mika.helminen@tuni.fi	FinnGen Teams	Trajectory
Tiina Luukkaala	University of Tampere, Tampere, Finland	tiina.luukkaala@tuni.fi	FinnGen Teams	Trajectory
Iida Vähätalo	University of Tampere, Tampere, Finland	iida.vahatalo@epshp.fi	FinnGen Teams	Trajectory
Jyrki Pitkänen	Institute for Molecular Medicine Finland (FIMM), HiLIFE, University of Helsinki, Helsinki, Finland	jyrki.pitkanen@helsinki.fi	FinnGen Teams	Data protection officer

For peer review only

For peer review only - http://bmjopen.bmj.com/site/about/guidelines.xhtml

Marco Hautalahti	Finnish Biobank Cooperative - FINBB	marco.hautalahti@finbb.fi	FinnGen Teams	FINBB - Finnish biobank cooperative
Johanna Mäkelä	Finnish Biobank Cooperative - FINBB	johanna.makela@finbb.fi	FinnGen Teams	FINBB - Finnish biobank cooperative
Sarah Smith	Finnish Biobank Cooperative - FINBB	sarah.smith@finbb.fi	FinnGen Teams	FINBB - Finnish biobank cooperative
Tom Southerington	Finnish Biobank Cooperative - FINBB	tom.southerington@finbb.fi	FinnGen Teams	FINBB - Finnish biobank cooperative

For peer review only

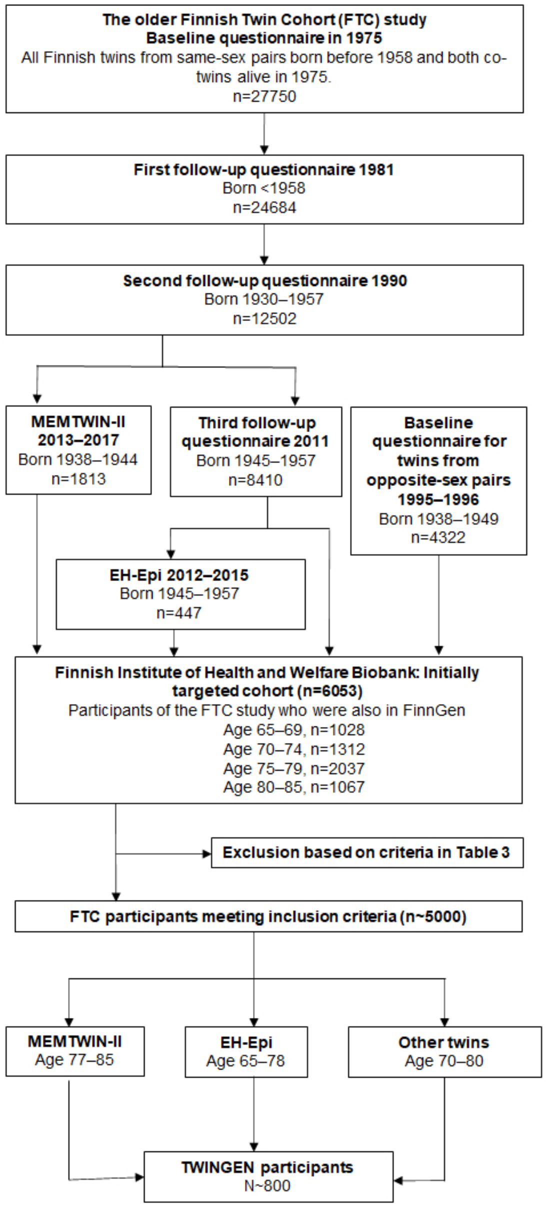

Flow-chart of study participant selection

127x277mm (96 x 96 DPI)

Supplementary Figure 1. Blood samples collected in TWINGEN

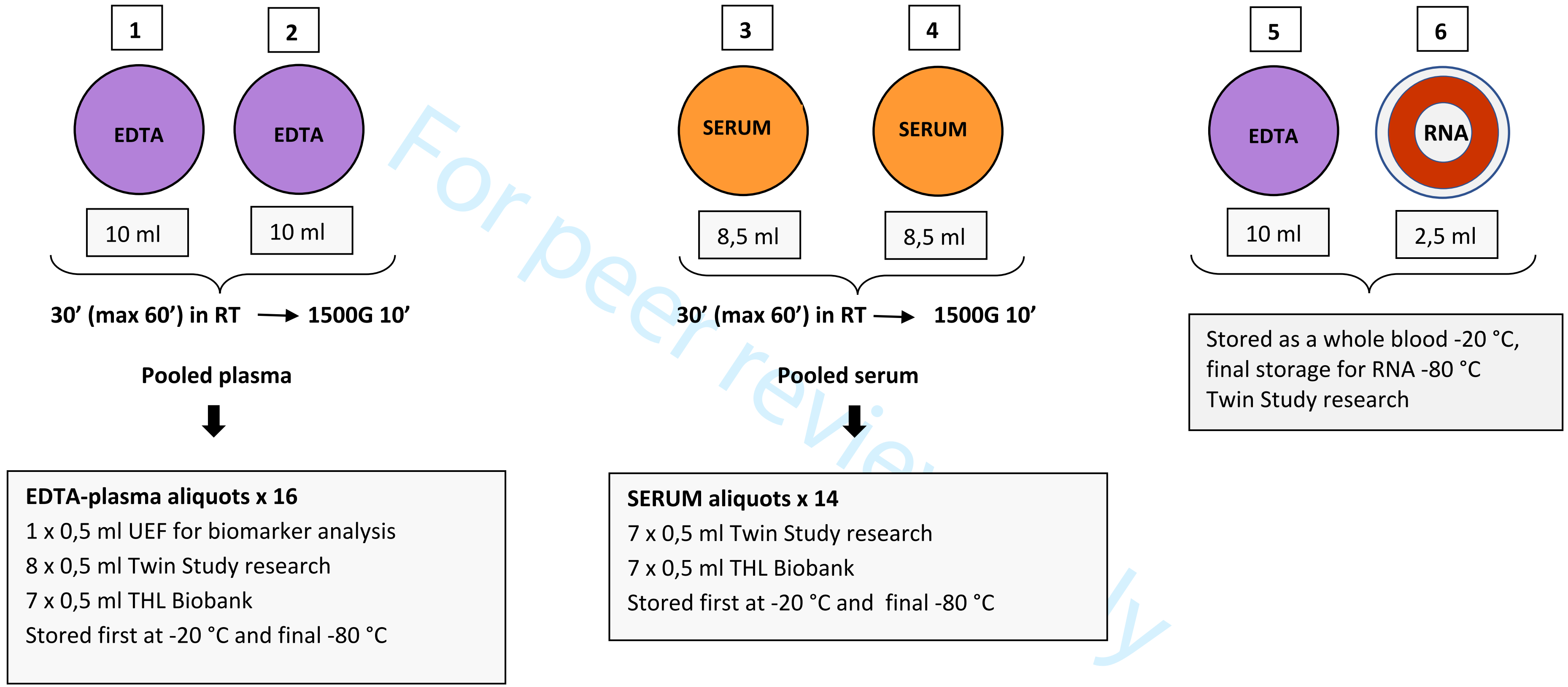

Supplementary Table 1. Previous data from the EH-Epi subcohort

Omics	n pairs (singletons)	Platform	Tissue	Study reference
Genotype	199 (4)	Illumina HumanCoreExome BeadChip	WBC	Drouard et al., 2022
DNA methylation	199 (4)	Illumina HumanMethylation450 BeadChip	WBC	Huang et al., 2020
Transcriptomics	195 (4)	Illumina HumanHT-12 v4 Expression BeadChip	WBC	Huang et al., 2018
miRNA	189 (3)	Illumina HiSeq	WBC	
Metabolomics	212 (10)	Nuclear magnetic resonance spectroscopy	Plasma	Drouard et al., 2022
Proteomics	210 (6)	Olink Explore	Plasma	

WBC = white blood cells

For peer review only

Supplementary Table 2. Additional references for studies on the older Finnish Twin Cohort study data

	References (PMIDs)
Education	37295953, 28975875, 23344794, 15368602, 11523706
Chronic or serious illness	3499347, 7629467, 9039240, 10208188, 15935024, 19210186, 19811382, 19952836, 20516052, 20680407, 21522028, 21612334, 30626223
Smoking	7198244, 7198250, 6984028, 2661052, 10857247, 12446254, 15053857, 15203778, 17174039, 17181913, 17549066, 19246432, 19423697, 19811391, 20418888, 20418890, 23752247, 24570102, 26359785, 26407342, 27134767, 29054888, 28399944, 29730550, 30001359, 31466478, 32157176, 32361366, 32938311, 35143046, 37322846
Alcohol use	27351919, 22215005, 20858964, 16222166, 12446254, 10857250, 1590545, 3335880, 3307505, 6984028, 7198250
Sleep	37322846, 37193395, 32907578, 29047168, 25451441, 23729932, 23509990, 21731146, 20883457, 19015202, 18275555, 17969458, 16230426, 14573377, 11525419, 11403523, 8642234
Medications	12446254, 27136415, 27723547
Anthropometrics (weight, height)	10702760, 10754982, 11523706, 11665321, 12953173, 14624724, 15368602, 15971946, 16484449, 18239571, 23298777, 27964777, 31364586, 31761633, 35534559
Blood pressure	6982602, 6540964, 2858745, 3101779, 3630648, 2661052, 1418922, 10781651, 12006398, 14624727, 16268117,17129598, 19952836, 20229313, 20680407, 29311254, 32520614, 35259029
Diabetes	1418922, 1473616, 10768089, 10753052, 14514580, 15105411, 15935024, 19952836, 20401462, 20680407, 20706830, 23124702, 26678054, 26847990, 36638183
Life satisfaction	22980535, 22215005, 19015202, 15656900, 15583908, 14982128, 12059849, 11576076, 11229985, 11092440, 9806309, 6540964, 6540959
Loneliness	See life-satisfaction papers, loneliness is one item in the LS scale
Cognition (TELE/TICS-m)	11893836, 14512720, 14707319, 16222166, 18025784, 19587088, 20858964, 21619517, 21700581, 23532744, 24082313, 26370660, 27752535, 27589524, 30090846, 30315123, 31477183, 32843700, 32954285, 33154181, 34607247, 35134847
Depressive symptoms	33629475, 32361366, 31469194, 28202098, 27723547, 27161145, 27055784, 25451441, 21330272, 17181913, 15607014, 15583908, 12558538, 10893486,
Personality (EPI)	8189349, 9806309, 11576076, 12115554, 19811701, 20639285, 24570102, 24828478, 26362575, 27507146, 6540964, 3335880, 23117535, 25993607, 27089181, 33629475
Accelerometer (physical activity)	35770444, 32259351, 30626223, 30445494, 30090846, 29925959
Multi-omics data	Also multiple metabolomics, transcriptomics and methylation (EWAS and epigenetic ageing) analyses
DNA	Multiple GWAS consortia papers, including PMIDs: 17549066, 22241830, 23752247, 26131930, 26407342, 27089181, 27134767, 27225129, 27911795, 28892072, 30038396, 30482948, 30643251, 31294817, 32157176, 36653562

For peer review only

EPI = Eysenck Personality Inventory; EWAS = epigenome-wide association study; GWAS = genome-wide association study; LS = Life Satisfaction; TELE = telephone assessment for dementia; TICS-m = modified Telephone Interview for Cognitive Status

For peer review only

BMJ Open

TWINGEN – protocol for an observational clinical biobank recall and biomarker cohort study to identify Finnish individuals with high risk of Alzheimer's disease

Journal:	*BMJ Open*
Manuscript ID	bmjopen-2023-081947.R1
Article Type:	Protocol
Date Submitted by the Author:	05-Mar-2024
Complete List of Authors:	Vuoksimaa, Eero; University of Helsinki Institute for Molecular Medicine Finland<br>Saari, Toni; University of Helsinki Institute for Molecular Medicine Finland<br>Aaltonen, Aino; University of Helsinki Institute for Molecular Medicine Finland<br>Aaltonen, Sari; University of Helsinki Institute for Molecular Medicine Finland<br>Herukka, Sanna-Kaisa; University of Eastern Finland Institute of Clinical Medicine, Neurology; Kuopio University Hospital, Department of Neurology, NeuroCenter<br>Iso-Markku, Paula; University of Helsinki Institute for Molecular Medicine Finland<br>Kokkola, Tarja; University of Eastern Finland Institute of Clinical Medicine, Neurology<br>Kyttälä, Aija; Finnish Institute for Health and Welfare, THL Biobank<br>Kärkkäinen, Sari; University of Eastern Finland Institute of Clinical Medicine, Neurology<br>Liedes, Hilkka; University of Helsinki Institute for Molecular Medicine Finland; VTT Technical Research Centre of Finland Ltd<br>Ollikainen, Miina; University of Helsinki Institute for Molecular Medicine Finland; Minerva Foundation Institute for Medical Research<br>Palviainen, Teemu; University of Helsinki Institute for Molecular Medicine Finland<br>Ruotsalainen, Ilona; University of Helsinki Institute for Molecular Medicine Finland; VTT Technical Research Centre of Finland Ltd<br>Toivola, Auli; Finnish Institute for Health and Welfare, THL Biobank<br>Urjansson, Mia; University of Helsinki Institute for Molecular Medicine Finland<br>Vasankari, Tommi ; UKK Institute; Tampere University Faculty of Medicine and Health Technology<br>Vähä-Ypyä, Henri; UKK Institute<br>Forsberg, Markus M.; University of Eastern Finland Faculty of Health Sciences, School of Pharmacy; VTT Technical Research Centre of Finland Ltd<br>Hiltunen, Mikko; University of Eastern Finland Institute of Clinical Medicine, Neurology<br>Jalanko, Anu; University of Helsinki Institute for Molecular Medicine Finland<br>Kälviäinen, Reetta; University of Eastern Finland Institute of Clinical

Medicine, Neurology; Kuopio University Hospital, Department of Neurology, NeuroCenter
Kuopio, Teijo; Wellbeing Services County of Central Finland and University of Jyväskylä, Central Finland Biobank
Lähteenmäki, Jaakko; VTT Technical Research Centre of Finland Ltd
Nyberg, Pia; Wellbeing Services County of North Ostrobothnia, Biobank Borealis of Northern Finland, Oulu University Hospital; University of Oulu, Translational Medicine Research Unit
Männikkö, Minna; University of Oulu, Arctic Biobank, Infrastructure for Population Studies, Faculty of Medicine
Serpi, Raisa; Wellbeing Services County of North Ostrobothnia, Biobank Borealis of Northern Finland, Oulu University Hospital
Siltanen, Sanna; Wellbeing Services County of Pirkanmaa, Finnish Clinical Biobank Tampere, Tampere University Hospital
FinnGen, -; -
Palotie, Aarno; University of Helsinki Institute for Molecular Medicine Finland; Broad Institute Stanley Center for Psychiatric Research
Kaprio, Jaakko; University of Helsinki Institute for Molecular Medicine Finland
Runz, Heiko; University of Helsinki Institute for Molecular Medicine Finland; Biogen Inc, Translational Sciences
Julkunen, Valtteri; University of Eastern Finland Institute of Clinical Medicine, Department of Neurology; Kuopio University Hospital, Department of Neurology, NeuroCenter

**Primary Subject Heading**:	Neurology
Secondary Subject Heading:	Epidemiology, Genetics and genomics
Keywords:	Dementia, GENETICS, Observational Study

SCHOLARONE™ Manuscripts

TWINGEN – protocol for an observational clinical biobank recall and biomarker cohort study to identify Finnish individuals with high risk of Alzheimer's disease

Eero Vuoksimaa[1]*, Toni T. Saari[1]*, Aino Aaltonen[1], Sari Aaltonen[1], Sanna-Kaisa Herukka[2,3], Paula Iso-Markku[1], Tarja Kokkola[2], Aija Kyttälä[4], Sari Kärkkäinen[2], Hilkka Liedes[1,5], Miina Ollikainen[1,6], Teemu Palviainen[1], Ilona Ruotsalainen[1,7], Auli Toivola[4], Mia Urjansson[1], Tommi Vasankari[8,9], Henri Vähä-Ypyä[8], Markus M. Forsberg[10,11], Mikko Hiltunen[2], Anu Jalanko[1], Reetta Kälviäinen[2,3], Teijo Kuopio[12], Jaakko Lähteenmäki[7], Pia Nyberg[13,14], Minna Männikkö[15], Raisa Serpi[13], Sanna Siltanen[16], FinnGen[†], Aarno Palotie[1,17,18], Jaakko Kaprio[1], Heiko Runz[1,19] & Valtteri Julkunen[2,3]

*Contributed equally, corresponding authors (eero.vuoksimaa@helsinki.fi; toni.saari@helsinki.fi)

[†]A list of authors and their affiliations appears at the end of the paper

[1]*Institute for Molecular Medicine Finland (FIMM), HiLIFE, University of Helsinki, Helsinki, Finland*

[2]*Department of Neurology, Institute of Clinical Medicine, University of Eastern Finland, Kuopio, Finland*

[3]*Department of Neurology, Neurocenter, Kuopio University Hospital, Kuopio, Finland*

[4]*THL Biobank, Finnish Institute for Health and Welfare, Helsinki, Finland*

[5]*VTT Technical Research Centre of Finland Ltd., Oulu, Finland*

[6]*Minerva Foundation Institute for Medical Research, Helsinki, Finland*

[7]*VTT Technical Research Centre of Finland Ltd., Espoo, Finland*

[8]*UKK Institute for Health Promotion Research, Tampere, Finland*

[9]*Faculty of Medicine and Health Technology, Tampere University, Tampere, Finland*

[10]*School of Pharmacy, Faculty of Health Sciences, University of Eastern Finland, Kuopio, Finland*

[11]*VTT Technical Research Centre of Finland Ltd., Kuopio, Finland*

[12]*Central Finland Biobank, Wellbeing Services County of Central Finland and University of Jyväskylä, Jyväskylä, Finland*

[13]*Biobank Borealis of Northern Finland, Oulu University Hospital, Wellbeing Services county of North Ostrobothnia, Oulu, Finland*

[14]*Translational Medicine Research Unit, University of Oulu, Oulu, Finland*

[15]*Arctic Biobank, Infrastructure for Population Studies, Faculty of Medicine, University of Oulu, Oulu, Finland*

For peer review only

For peer review only - http://bmjopen.bmj.com/site/about/guidelines.xhtml

[16]Finnish Clinical Biobank Tampere, Tampere University Hospital, Wellbeing Services County of Pirkanmaa, Tampere, Finland

[17]Analytic and Translational Genetics Unit, Department of Medicine, Department of Neurology and Department of Psychiatry, Massachusetts General Hospital, Boston, MA, USA.

[18]The Stanley Center for Psychiatric Research and Program in Medical and Population Genetics, The Broad Institute of MIT and Harvard, Cambridge, MA, USA

[19]Translational Sciences, Biogen, Cambridge, MA, USA

For peer review only

Abstract

Introduction: A better understanding of the earliest stages of Alzheimer's disease (AD) could expedite the development or administration of treatments. Large population biobanks hold the promise to identify individuals at an elevated risk of AD and related dementias based on health registry information. Here, we establish the protocol for an observational clinical recall and biomarker study called TWINGEN with the aim to identify individuals at high risk of AD by assessing cognition, health and AD-related biomarkers. Suitable candidates were identified and invited to participate in the new study among THL Biobank donors according to TWINGEN study criteria.

Methods and analysis: A multi-center study (n=800) to obtain blood-based biomarkers, telephone-administered and web-based memory and cognitive parameters, questionnaire information on lifestyle, health and psychological factors, and accelerometer data for measures of physical activity, sedentary behavior and sleep. A sub-cohort are being asked to participate in an in-person neuropsychological assessment (n=200) and wear an Oura ring (n=50). All participants in the TWINGEN study have genome-wide genotyping data and up to 48 years of follow-up data from the population-based older Finnish Twin Cohort (FTC) study of the University of Helsinki. The data collected in TWINGEN will be returned to THL Biobank from where it can later be requested for other biobank studies such as FinnGen that supported TWINGEN.

Ethics and dissemination: This recall study consists of FTC/THL Biobank/FinnGen participants whose data were acquired in accordance with the Finnish Biobank Act. The recruitment protocols followed the biobank protocols approved by Finnish Medicines Agency. The TWINGEN study plan was approved by the Ethics Committee of Hospital District of Helsinki and Uusimaa (number 16831/2022). THL Biobank approved the research plan with the permission no: THLBB2022_83.

Keywords: Alzheimer's disease, biobank, recall study, cognition, biomarkers

For peer review only - http://bmjopen.bmj.com/site/about/guidelines.xhtml

Article summary

Strengths and limitations of this study

- A large sample of individuals is recruited from a representative biobank database
- Using health registry information, we exclude those with documented AD or other neurological or psychiatric diseases that can affect cognition. Pre-screening limits the sending of unnecessary invitations and saves costs
- Participants have up to 48 years of follow-up questionnaire and clinical data from the Finnish Twin Cohort study and these data can be combined with multifaceted Finnish health registry information. Previous genotype data is available in the biobank from all TWINGEN study participants.
- We assess the feasibility of remote cognitive testing and blood samples in large-scale screening of AD risk, translating to the requirements of intervention trials and clinical practice
- Limitations of the study are a lack of gold standard biomarkers (cerebrospinal fluid, positron emission tomography imaging) and neurological examinations

Word count: 4385

For peer review only

Introduction

Alzheimer's disease (AD) – the most common cause of dementia – is characterized by pathological accumulation of beta-amyloid (Aβ) and tau in the brain (1). As populations age, the prevalence of dementia is projected to nearly double every two decades (1,2) and AD and related dementias are becoming one of the most common causes of death in many countries (20% of deaths in Finland ranking it as the third most common cause of death) (3). The AD disease process starts up to 20-30 years before the diagnosis, so intervention trials targeted at preclinical or prodromal stages of AD are of high priority, but time-consuming and costly with screen-failure rates of 78-88% (4).

In clinical practice, AD is diagnosed mainly based on the clinical phenotype, episodic memory impairment being the cognitive hallmark, while in research there has been a shift from clinical diagnosis to biological classification independent of the cognitive status (5). Evidence of diagnostic properties of different blood-based biomarkers is rapidly accumulating (6), but population-based studies are still scarce. In one population-based study, 11% of older adults (median age 74 years) without dementia were found to have Aβ pathology using blood-based biomarkers (7).

Our earlier pilot study explored the feasibility of FinnGen, a nationwide Finnish biobank study (8) in recruiting individuals with AD to an observational study via the biobank (9). Our protocol included blood-based biomarkers and remote cognitive assessment, approaches that are suggested to improve the recruitment of participants for AD trials (10–12). Another FinnGen study showed that biobank participants could be recontacted for additional data collection in a larger scale as well (13). The full potential of population biobank datasets lies in the large cohorts of undiagnosed individuals who are at the risk of developing AD in near future and might be suitable for targeted screening for early diagnostics and interventional trials encompassing both pharmacological treatments and lifestyle interventions.

After showing in our pilot study that recall of biobank participants with AD to a clinical study including multimodal (remote and in-person) cognitive assessment and blood-based biomarker

For peer review only - http://bmjopen.bmj.com/site/about/guidelines.xhtml

analyses is feasible (9), we modified the protocol of the pilot study to target cognitively unimpaired older adults (based on health registry data) in the current study. Additionally, we augmented the assessment battery with passive technology for measuring physical activity, sedentary behavior, and sleep.

This protocol paper describes TWINGEN, a population-based follow-up study investigating the utility of easily implementable methods for assessing the risk of AD. We aim to conduct a proof-of-principle study for using biobank registries as a platform for recruiting participants suitable for clinical trials, particularly in diseases where recruitment and screening has generally been challenging. We also focus on the remote cognitive assessment methods and blood-based biomarkers of AD. The study also aims to enrich existing biobank data derived from a long-standing prospective twin study with cognitive and lifestyle measures. The research setting is unique as it utilizes the pre-screening and recall option based on the data of the biobank in combination with long preceding population-based follow-up data from the twin study.

Methods and analysis

Study participant selection

The target group of the TWINGEN study are individuals who have participated in the older Finnish Twin Cohort (FTC) study of the University of Helsinki (UH), and whose samples and data have been transferred to THL Biobank in 2018. The FTC cohort was chosen as the primary target of this study because it is a population-based follow-up study with up to 48 years of previous comprehensive health data available. Combining the historic data with newly collected samples would allow building of longitudinal trajectories of various lifestyle and health factors to late-life cognitive decline. The main selection criteria in the biobank were previous participation in FTC, age (65–85), place of current residence in Finland, Finnish as the first language, and no known diagnosis affecting cognition in biobank records. The selection of eligible FTC study participants was done through THL Biobank and data collection was carried out by the University of Helsinki for

For peer review only - http://bmjopen.bmj.com/site/about/guidelines.xhtml

those living in greater Helsinki area or surrounding regions and by regional biobanks and Turku University of Applied Science based on the residency of the participant. Selection also included participation in the FinnGen nationwide biobank research study for two reasons (8). First, FinnGen supported the collection of the TWINGEN cohort with the aim to enrich phenotype information. Second, the collected TWINGEN data are to be returned to THL Biobank, from where they can later be requested for the FinnGen study and combined with its extensive gene and health register data. Thus far, FinnGen has produced genotype data from ca. 500 000 biobank donors of all Finnish biobanks to perform large scale genome and health research. Below, we describe each of the data sources and the study protocol of TWINGEN.

The older FTC study

The older FTC study from the University of Helsinki is a population-based study that includes all Finnish same-sex twins born before 1958 and living in Finland at the start of the study in 1974 (Figure 1) (14). The baseline survey was conducted in 1975 via postal questionnaire and 27750 individuals participated with an 89% participation rate. Follow-up questionnaires were sent in 1981 (n=24684 with a 84% response rate), 1990 for those born in 1930 or later (n=12502; 77%), and in 2011–2012 for those born in 1945–1957 (n=8410; 72%) (15). Those born in 1938–1944 have also participated in MEMTWIN II study (n=1772) that utilized telephone interview to assess cognition (16). Some of the twins born in 1945–1957 have participated in Essential Hypertension EPIgenetics study (EH-EPI, n=445) (17). The MEMTWIN II and EH-EPI study participants are the primary groups of interest in TWINGEN because they have either earlier cognitive (MEMTWIN II; Table 1) or multi-omics data (EH-EPI; Supplementary Table 1) available.

For peer review only - http://bmjopen.bmj.com/site/about/guidelines.xhtml

Table 1. Cognitive measures used in the TWINGEN study

In-person neuropsychological assessment	Telephone (TELE/TICS-m3)	Web-based computerized assessment (cCOG)
**Memory and learning**		
CERAD Word List Learning	Word List Learning	Episodic Memory Learning
CERAD Word List Recall	Word List Recall	Episodic Memory Recall
CERAD Word List Recognition		Episodic Memory Recognition
CERAD Constructional Praxis Recall		
WMS-III Logical Memory Story A		
WMS-III Logical Memory Story A Recall		
**Executive function**		
Trail Making Test-B		Modified Trail Making Test-B
Stroop Interference		
Stroop Set-Shifting		
**Visuospatial skills & visuoconstruction**		
CERAD Constructional Praxis		Fragmented Letters
CERAD Clock Drawing Test		
**Language skills & fluency**		
CERAD Naming Test	Similarities	
CERAD Semantic Fluency	Semantic Fluency	
**Processing speed**		
Trail Making Test-A		Modified Trail Making Test-A
Stroop Word Reading		
Stroop Color Naming		
**Global cognition**		
CERAD MMSE	TELE Global Score	cCOG Global Score
	TICS Global Score	
	TICS-m3 Global Score	

MEMTWIN-II participants have prior TELE/TICS-m data. CERAD-nb = Consortium to Establish a Registry for Alzheimer's Disease – neuropsychological battery; MMSE = Mini-Mental State Examination; TELE = telephone assessment for dementia; TICS = Telephone Interview for Cognitive Status; TICS-m = modified Telephone Interview for Cognitive Status; TICS-m3 = TICS-m including three learning trials in the Word List Learning; WMS-III = Wechsler Memory Scale 3rd edition

For peer review only

To achieve the target number of 800 participants, the biobank selection was expanded to the twins born in 1945–1952 who had not participated in MEMTWIN II or EH-EPI. Most of the invited twins were from same-sex pairs, but we also invited twins from opposite-sex pairs included in the older FTC study in year 1995–96, when they replied to a brief health questionnaire (Figure 1). We prioritized invitations to participants who lived closest to one of the six study sites. All TWINGEN participants from same-sex twin pairs have longitudinal questionnaire data on health and health-related behaviors from years 1975, 1981 and 1990, and those born in 1945–1957 have data also from year 2011–2012. An overview of longitudinal data available for the MEMTWIN II and EH-EPI and other TWINGEN participants are presented in Table 2. Necessary inclusion criteria for TWINGEN were available DNA sample in THL Biobank and not having any of the exclusion criteria (Table 3). Additional references for studies using the older FTC data are found in Supplementary Table 2.

For peer review only

For peer review only - http://bmjopen.bmj.com/site/about/guidelines.xhtml

Table 2. Previous data from the participants recruited in TWINGEN

	Baseline assessment (1975)	First follow-up (1981)	Second follow-up (1990)	Third follow-up (2011-2012)	MEMTWIN-II (2013-2017)*	EH-Epi (2012-2015)	Primary study reference
Education	x	x					Vuoksimaa et al., 2016b
Chronic or serious illness	x	x	x	x	x	x	Kaprio et al., 2019
Physical activity	x	x	x	x		x	Piirtola et al., 2017
Smoking	x	x	x	x		x	Kaprio & Koskenvuo 1988
Alcohol use	x	x	x	x		x	Sipilä et al., 2016
Sleep	x	x	x	x			Kaprio et al., 2019
Medications	x	x	x	x	x	x	Huang et al., 2018
Anthropometrics (weight, height)	x	x	x	x		x	Piirtola et al., 2017
Dietary habits		x				x	Kaprio et al., 2019
Blood pressure	x	x	x	x		x	Iso-Markku et al., 2021
Cholesterol		x	x	x			Iso-Markku et al., 2021
Diabetes	x	x	x	x		x	Iso-Markku et al., 2021
Subjective memory complaints					x		
Life satisfaction	x	x	x	x			Koivumaa-Honkanen et al., 2000
Loneliness	x	x	x	x			Koivumaa-Honkanen et al., 2000
Social support			x	x			Romanov et al., 2003
Cognition (TELE/TICS-m)					x		Lindgren et al., 2019
Depressive symptoms			BDI	CES-D	CES-D		Saari et al., 2023
Personality (EPI)	x	x		x			Rose et al., 1988
Accelerometer (physical activity)					x		Waller et al., 2019
Multi-omics data						x	Drouard et al., 2022
DNA					x	x	Kaprio et al., 2019

For peer review only

For peer review only - http://bmjopen.bmj.com/site/about/guidelines.xhtml

BDI = Beck Depression Inventory; CES-D = Center for Epidemiological Studies-Depression scale; EPI = Eysenck Personality Inventory; LS = Life Satisfaction; TELE = telephone assessment for dementia; TICS-m = modified Telephone Interview for Cognitive Status. See Supplementary Table 2 for full reference information and additional references on previous studies using older Finnish Twin Cohort study data. *Third follow-up assessments for those born in 1938–1944.

For peer review only

For peer review only - http://bmjopen.bmj.com/site/about/guidelines.xhtml

Table 3. Exclusion criteria for the TWINGEN study

ICD-10 code	Explanation
G30 and F00	Any variant of Alzheimer's disease or any dementia relating to Alzheimer's disease, dementia with Lewy bodes, frontotemporal dementia and mixed dementia
F01-F03	Dementia, any etiology
G20	Parkinson's disease
G35	Multiple sclerosis
I60, I61 and I63	Intracerebral hemorrhage, subarachnoid hemorrhage or ischemic stroke and their subcategories
S06.1-S06.7	Traumatic brain injuries other than concussion (S06.0)
F20	Schizophrenia
F31	Bipolar disorder
F33, F32.1-F32.3, F34	Recurrent depression, moderate or severe depression and long-lasting mood disorders
F60	Personality disorders
F10	All diagnoses relating to excessive usage of alcohol
F11-F19	Intoxication because of opioids, cannabinoids, sedative medication, cocaine or hallucinogens
F70-73	Different stages of intellectual disabilities

For peer review only

Recall procedure

The results of a THL Biobank feasibility assessment indicated that there were 6053 individuals aged 65–85 who have participated in the FTC of UH and have data in THL Biobank. Approximately 1000 individuals were excluded due to our exclusion criteria of Alzheimer's disease and other neurological or psychiatric diseases that can affect cognition (Figure 1; Table 3). Furthermore, the genotype data of approximately 100 individuals have been verified by FinnGen, but these data have not been returned to their respective biobanks at the time of the feasibility assessment; thus, these individuals are excluded from the pool of potentially eligible participants. The target sample size of 800 participants was chosen as it fits the timeframe and resources of the study and is sufficiently large for stratifying participants at varying risks for AD (7).

As a biobank recall study, TWINGEN participants are contacted by THL Biobank by an invitation letter. The invitation letter includes information about the individuals' prior participation to the older FTC study and the transfer to and storage of samples and data at THL Biobank, information about the participation in the FinnGen study via the biobank, and information about the new TWINGEN study. The invitation letter also contains two separate consent forms: one for the participation in the TWINGEN study and the biobank consent for THL Biobank. The consent for THL Biobank is needed to store the new samples and data obtained in TWINGEN to THL Biobank and to confirm the biobank participation with a written biobank consent, which is the primary basis for storing samples and data into a biobank. After receiving consent forms, UH research staff contacts the potential participants to verify that the individuals have understood the purpose and procedures of the study. Additionally, as the health registry data available in the biobank is not up to date but reflects status at the end of year 2022, research staff verifies (via telephone) that the exclusion criteria are not met. Eligibility of each individual is assessed independently of their co-twin's vital status or eligibility.

For peer review only - http://bmjopen.bmj.com/site/about/guidelines.xhtml

Data collection

Data collection started on March 29th 2023 and is planned to be completed by the end of 2023. Data collection is conducted in six locations across Finland: at the Institute for Molecular Medicine Finland (FIMM), University of Helsinki in Helsinki, biobanks in four locations across Finland: in Jyväskylä (Central Finland biobank), Kuopio (Biobank of Eastern Finland), Oulu (Arctic Biobank and Biobank Borealis of Northern Finland) and Tampere (Finnish Clinical Biobank Tampere) and at the clinical laboratory of Turku University of Applied Sciences. Protocol for all participants includes telephone- and computer-administered cognitive testing, blood-draw and self-report questionnaire. Additionally, a waist-worn accelerometer will be given to participants who are willing to complete one week at-home measurement. Furthermore, individuals living in greater Helsinki area are invited to participate in in-person neuropsychological testing (target sample size n=200) and measurement of weight, height, waist circumference and blood pressure. In Helsinki, we also provide Oura rings for 50 participants who are willing and able to wear the ring and use the associated mobile app. After participating in the study, participants receive a report based on their performance in telephone interview-based and computerized tests of cognition and about their physical activity. Data cleaning, feedback to participants and transfer of data to THL biobank are estimated to be completed by the end of June 2024. A detailed description of the data collections is provided below.

Telephone interview for assessing cognitive status and function

Two validated telephone-administered cognitive screening instruments are used: a telephone assessment for dementia (TELE) (18) and the modified Telephone Interview for Cognitive Status (TICS-m) (19). TELE and TICS have been translated to Finnish and adapted to Finnish culture (20) and both have been used in the older FTC study. TICS-m, a modified version of TICS includes an additional delayed free recall of the 10-word list and has been used in the MEMTWIN II sub-study

For peer review only

(21). We further modified the TICS-m by including three learning trials of the 10-word list and this instrument is later referred to as TICS-m3 (9). We also included semantic fluency. Three trial word list task yields immediate and delayed free recall measures of episodic memory. Semantic fluency score is the number of animals named in 1 minute (Table 1). In the telephone interview, the participants are also asked about their functional abilities regarding household maintenance, ambulation, shopping, dressing and undressing, use of mobility aids, memory problems and possible visits to the doctor regarding memory problems. Telephone interviews were conducted by trained study nurses or psychologists.

Computerized web-based cognitive testing

Web-based cCOG tool created by Combinostics (Tampere, Finland) is used for computerized cognitive testing (22). It includes six subtests: Episodic Memory (learning & recall), Reaction Time, Modified Trail Making A and B and Distorted Letters. The tasks measure visual processing, memory, processing speed, attention, and executive function (Table 1). In addition to cognitive tests, cCOG includes background questions (education) and a seven-item questionnaire designed to assess probable dementia with Lewy bodies (23). The test battery takes about 25 minutes to complete and is performed via a keyboard and a mouse or a touchscreen device.

In-person neuropsychological tests

The participants in Helsinki study site undergo an in-person neuropsychological assessment, with a target sample size of 200 individuals. The minimum sample size to detect medium correlations ($r = .3$) between cognitive measures with a significance level = .05 and power = .8 is $n = 85$, thus, the target sample size of 200 individuals with in-person cognitive assessment is adequate for examining correlations between in-person and remote cognitive measures. The larger sample size is expected to be adequate for factor analyses of the neuropsychological battery.

For peer review only

For peer review only - http://bmjopen.bmj.com/site/about/guidelines.xhtml

The in-person neuropsychological assessment consists of the Consortium to Establish a Registry for Alzheimer's Disease neuropsychological battery (CERAD-nb) (24) and tests measuring executive functions, processing speed and episodic memory. The CERAD-nb includes Mini-Mental State Examination and Semantic Fluency, abbreviated Boston Naming Test, Word List Learning, Recall and Recognition, and Constructional Praxis (Copy and Recall). The Finnish version of the CERAD-nb also includes Clock Drawing test (25). Finnish education adjusted cut-offs are available for total score and for each sub-test (26). In addition to the CERAD-nb, we include the following tests: Logical Memory story A from the Wechsler Memory Scale 3rd edition (27), Trail Making Test A and B (28), and Stroop test (29). The version of Stroop used in this study is the 40-item version used in the FINGER study (30) with an additional fourth “set-shifting” condition whereby the task is to name the color of ink (as in the classical Stroop condition) or to read out the color-word when the word is inside a rectangle. Neuropsychological tests are administered by trained psychologists. A summary of key cognitive measures of all three modalities are presented in Table 1.

Blood sample

A venous blood sample is drawn from the inside of the elbow or alternatively from the back of the hand. A total of six tubes are collected: three BD Vacutainer K2EDTA (10/10ml) tubes, two BD Vacutainer SSTII Advance-serum gel tubes (10/8.5ml), and one BD PAXgene Blood RNA (7/2.5ml) tube (Supplementary Figure 1). Processing will be done immediately after the samples have been taken. Serum tubes are allowed to clot at least 30 minutes (max. 60’) before separating. EDTA-plasma tubes do not clot but two EDTA tubes are also let sit for the same time as serum because of the easier workflow. Serum and two EDTA-plasma tubes are centrifuged (1500G) for 10’ and the supernatants are pooled within serum and plasma. Samples are apportioned into 0.5 ml aliquots and stored at -20°C. One EDTA tube and PAXgene RNA-tube will be stored as a whole blood at -20°C. Serum, plasma and RNA samples final storage temperature is at -80°C. One of the 0.5 ml

For peer review only

EDTA-plasma aliquot will be sent to University of Eastern Finland for biomarker analysis. RNA, EDTA whole blood samples, and half of the serum and plasma aliquots are dedicated to the Twin Study, while the other half will be available for research via THL Biobank.

Blood-based AD biomarkers

The primary blood-based biomarkers include phosphorylated-tau181 (p-tau181), phosphorylated-tau217 (p-tau217), Aβ1-42/40, glial fibrillary acidic protein (GFAP) and neurofilament light chain (NfL); all measured using Simoa HD-X analyzer (Quanterix, Billerica, Massachusetts, USA). Plasma p-tau181 levels are quantified using Simoa p-tau181 Advantage V2.1 Kit(Ref# 104111, Quanterix) (31), Aβ1-40, Aβ1-42, GFAP and NfL levels using Simoa Neurology 4-Plex E Advantage Kit (Ref# 103670, Quanterix) (32), and p-tau217 levels using ALZpath Simoa pTau-217 v2 Assay Kit (Ref# 104371, Quanterix) (33). Prior to analyses, EDTA plasma samples are thawed, mixed and centrifuged (10,000xg, 5 min, +20 °C). These biomarkers are determined at the Biomarker Laboratory of University of Eastern Finland.

Apolipoprotein E (APOE) genotype and polygenic risk scores

APOE status (ε4-carrier vs. non-carrier and number of ε4-alleles) is defined by two single-nucleotide polymorphisms, rs429358 and rs7412, in chromosome 19(34) and polygenic risk score (PRS; with and without APOE) of AD is based on the Bellenguez et al. (35) or newer meta-analysis if available. Genetic data will be used to calculate also PRS's for diseases and traits (such as cardiovascular disease and educational attainment) that are related to risk and protective factors of dementia (36).

Accelerometer-measured physical activity, sedentary behavior, and sleep

A tri-axial accelerometer (UKK RM42, UKK Terveyspalvelut Oy, Tampere, Finland) is used to monitor participants' daily physical activity, sedentary behavior, and sleep for seven consecutive

For peer review only - http://bmjopen.bmj.com/site/about/guidelines.xhtml

days (Table 4) (37). In addition to total time spent in physical activity of different intensities and sedentary behavior, we will also measure number of bouts and length of the bouts of physical activity and sedentary behavior. The participants receive the devices during their in-person visit at FIMM or by mail if participating in other location. Participants are asked to wear the accelerometer on the hip during waking hours and on the wrist during sleep. At least a 4-day monitoring period with a minimum of 10 hours wear-time a day will be required for the adequate accelerometer data collection (38).

The UKK RM42 device and its closely related counterpart Hookie AM 20 accelerometer have been used in samples with over 18,000 Finnish 18–85-year-old adults (37,39). Thus, the UKK RM42 accelerometer is usable in the TWINGEN sample of 65–85-year-olds, and we will get an opportunity to compare the measurements against normative data of Finnish adults. The analyses of raw acceleration data of the UKK RM42 are based on validated algorithms; the technical details related to the recording and analyzing of raw acceleration data are given elsewhere (40–42).

For peer review only - http://bmjopen.bmj.com/site/about/guidelines.xhtml

Table 4. Core UKK RM42 accelerometer and Oura ring parameters

The UKK RM42 accelerometer	Oura ring
Target n = 800	Target n = 50
Participants from all sites	Participants from Helsinki site
Wearing time = 1 week	Wearing time = 2 weeks
**Physical activity parameters**	
Light physical activity	Low intensity activity
Moderate physical activity	Medium intensity activity
Vigorous physical activity	High intensity activity
Total physical activity	Total physical activity
Number of steps	Number of steps
Standing time	
**Sedentary behavior parameters**	
Lying time	Inactive time
Reclining time	Resting time
Sitting time	
Number of breaks during sedentary time	
**Sleep parameters**	
Total sleep time	Total sleep time
Restless sleep time	Restless sleep percentage
Restful sleep time	Total amount of deep sleep
	Total amount or REM sleep
	Total amount of light sleep

Oura-measured physical activity, sedentary behavior and sleep

Oura ring (Gen3 Heritage, Ōura Health Ltd., Oulu, Finland) will be given to 50 participants in Helsinki study collection site. The number of participants asked to wear an Oura ring was an experimental pilot within our larger study. The target sample of 50 was purposed to evaluate the feasibility of a measurement requiring a smart phone app in older adults. First, participants use a ring-size kit to determine optimal ring size and then they receive the ring either at their in-person visit or by mail. Participants are asked to wear the ring (width: 7.9mm, thickness: 2.55 mm, weight:

For peer review only - http://bmjopen.bmj.com/site/about/guidelines.xhtml

4–6g) in any finger for two weeks during day and night, except when charging the ring every 4–6 days (20–80 minutes to fully charge). To monitor participant's sleep, sedentary behavior and physical activity (Table 4), the Oura ring uses infrared photoplethysmography sensors, negative temperature coefficient sensor and 3D accelerometer. Participants receive written instructions on using the Oura ring and downloading the Oura mobile application, through which they can access their own data. If necessary, the research staff provides phone guidance for both using the ring and installing the application. The data from Oura ring is transferred to participant's Oura application when opening the application and to a cloud server. Data collection will be monitored from Oura cloud server and participants' will be sent a reminder if there are no data from previous 2 days.

The Oura ring's sleep stage detection algorithm (wake, light non-rapid eye movement [NREM] sleep, deep NREM sleep, rapid eye movement [REM] sleep) has been validated against polysomnography and it showed 80–96% accuracy, 74–82% sensitivity and 79–98% specificity (43). Furthermore, moderate-to-vigorous intensity physical activity and step count of Oura ring has shown strong correlations with accelerometer-measured corresponding values (44).

Questionnaire

Participants are given a 16-page self-report questionnaire that includes many of the same measures as in previous questionnaires in years, 1975, 1981, 1990 and 2011 (Table 2). Questions cover anthropometrics, demographics, social relationships, chronotype, health (general, cardiovascular, dementia, memory, medications, vision, hearing, balance and mobility) and health-related behavior including sleep, physical activity, smoking and alcohol use. Psychological wellbeing scales included in the questionnaire are: 8-item Center for Epidemiologic Studies Depression (45,46); 7-item Purpose in Life subscale from Ryff's Scales of Psychological Well-Being (47,48); Extraversion (9 items) and Neuroticism (10 items) from the short version of Eysenck

For peer review only - http://bmjopen.bmj.com/site/about/guidelines.xhtml

Personality Inventory (49,50); and four-item life satisfaction scale derived from questionnaires by Allardt (51,52).

Patient and public involvement statement

None.

Aims, data analysis and future directions

In addition to the overarching aim of assessing the feasibility of biobank recall in the context of preclinical AD, we also have more focused research questions (see Supplementary Figure 2 for an overview of aims and associated data). In the initial stage of TWINGEN, we cannot make clinical or research diagnoses of AD, however, we will be able to follow these individuals through national health registry information that are compiled in FinnGen. Registry-based data will allow to predict progression to AD. Possible follow-up visits can also include gold standard measures for diagnosing AD, such as cerebrospinal fluid or positron emission tomography imaging.

By combining all data, we aim to stratify our participants in sub-groups of low, intermediate and high AD risk based on genetic, biomarker, cognitive, lifestyle and symptom data. The stratification will be based on a combination of percentiles or cut-offs (e.g., Ashton et al.[53]) for blood-based biomarkers, cut-offs for cognitive impairment in CERAD (26), cCOG (22) and TELE/TICS (16), lifestyle risk scores and subjective memory complaints. We also aim to use neuropsychological criteria for mild cognitive impairment classification where -1 SD performance in at least two tests are required independent of subjective memory complaints (54,55). Biomarkers and cognitive data allow to derive sub-groups based on biomarker profile and cognitive stating included in the AT(N) framework (5). Additionally, we will use APOE status and PRS for genetic risk profiling although genetics are not included in the AT(N) framework.

For peer review only - http://bmjopen.bmj.com/site/about/guidelines.xhtml

The stratification approaches are potentially useful for improving participant selection for AD drug and intervention trials. These methods would also be valuable in clinical settings where non-invasive and widely available tools for evaluating the presence of AD pathology underlying cognitive symptoms is important, especially once disease-modifying treatments become available. TWINGEN will also establish a baseline cohort that can be used in follow-up studies with neuroimaging and cerebrospinal fluid biomarkers.

We aim to assess the comparability of in-person, telephone-based and computerized cognitive assessments using correlation analysis of total scores and tests of different modalities assessing the same cognitive domains (e.g., memory). We will also explore the distributions and correlations among blood-based biomarkers of AD-related pathologies and investigate the associations between cognition and biomarkers. Cognition will be treated both categorically (cognitive status) and as continuous outcomes for domain-specific measures (e.g., episodic memory, executive function). For the domain-specific cognitive composites, factor scores will be calculated similar to previous studies on preclinical AD and mild cognitive impairment (56,57). The factor scores will be based on exploratory factor analyses of the in-person neuropsychological battery and used instead of individual tests when the interest is on a cognitive domain, not a single test.

Additionally, the effect of genetic and lifestyle (with up to 48 years of follow-up) factors can be used in predicting cognitive and biomarker status. The measures of physical activity, sedentary behavior and sleep (Table 4) allow us to explore the relationships between physical activity and sleep with cognition and biomarkers. The list of physical activity and sleep parameters in Table 4 is not exhaustive and more parameters are available for detailed analyses.

Although inclusion in the study was not dependent on the co-twin's participation, it is expected that full twin pairs will also participate. This will allow studying if the between-family associations are

For peer review only - http://bmjopen.bmj.com/site/about/guidelines.xhtml

also evident in within-family comparisons and we can identify twin pairs who are discordant for cognition or biomarkers (58).

Ethics and dissemination

According to the Finnish Biobank Act, research collections, which have been collected, or whose collection started before the Biobank Act became into force on 1 September 2013, can be transferred to a biobank by a specific procedure, which includes an ethical evaluation and informing the sample donors either personally or by public announcement. Accordingly, biological samples of the FTC, including DNA and associated data were transferred to THL biobank in December 2018 to facilitate biobank research. The action of transferring FTC data to THL Biobank was publicly announced in major newspapers.

Recruitment protocols followed the biobank protocols approved by Fimea. The FinnGen study was approved by the Coordinating Ethics Committee of Hospital District of Helsinki and Uusimaa (HUS; statement number HUS/990/2017). The permit numbers of the decisions made by Finnish Institute for Health and Welfare (THL) and the Biobank Access Decisions for FinnGen samples and data utilized in FinnGen Data Freeze 9 are presented in Acknowledgements.

Before entering the TWINGEN study all potential study participants received detailed information regarding this study by a formal information letter. All study participants were asked for and provided written informed consent. The recall study was reviewed and approved by the ethics committee of HUS (approval number 16831/2022). THL Biobank approved the research plan with the permission no: THLBB2022_83.

The data acquired in this study are managed and initially stored by UH. Keeping with the Biobank Act and the consent given by the participants, the data are also transferred to THL Biobank for later research use. FinnGen request to use the TWINGEN data from THL Biobank, after which the

For peer review only

data acquired in this study are linked with existing genetic and register data in a controlled FinnGen sandbox environment.

The data acquired in this study is subject to conditions of the IRB protocols and the policies of the Finnish biobank legislation and therefore unavailable for unsupervised usage. The data is stored in FIMM and THL Biobank, where approved researchers can access the data. Eventually the data will also be transferred to the secure FinnGen sandbox environment and linked to the registers available in FinnGen. The results of the study will be published in peer-reviewed journals and presented at scientific conferences.

Discussion

The TWINGEN study uses biobank registries for a recall study with the aim of identifying individuals at risk of AD. The collected data comprise many known risk factors (36) and scalable screening methods of AD. While these data are expected to yield novel insights even in isolation, the unique potential for discovery comes from combining the existing follow-up and newly collected data of TWINGEN participants with the existing data. Although this study has many strengths, a limitation is the lack of gold standard biomarkers (cerebrospinal fluid, positron emission tomography imaging) and neurological examinations in our baseline assessment.

Since its inception, the FinnGen project has created new expansion areas with the aim of enriching phenotype information. The TWINGEN study addresses this goal by collecting cognitive, physical activity, lifestyle and biological data that are returned to FinnGen via THL biobank, whereas FinnGen would allow for a registry-based follow-up of the TWINGEN study participants.

For peer review only - http://bmjopen.bmj.com/site/about/guidelines.xhtml

Author contributions

TTS: writing the original draft, revision and data collection. EV: writing the original draft, revision, resources, concept, design and supervision. AA: writing, revision and data collection. IR, HL, S-KH, SK, TK, SA: writing. MO, P-IM: revision. AK: design, administration and revision. AT: study coordination and revision. TV, HV-Y, MM, RS, PN, SS: resources and revision. V-MK, TK, EP, MR: resources. JK: concept, design, revision and resources. MU: study coordination, data collection. VJ: concept, design, revision, supervision. MMF, MH, JL, RK: consultation in the design of the study. HR: concept, design, revision. AP: concept, resources, revision. TP: data management. AJ: administration. FinnGen: administration, resources, funding. TS and EV contributed equally to this paper.

Acknowledgements

We thank THL Biobank and biobank directors Eero Punkka (Helsinki Biobank) and Veli-Matti Kosma (Biobank of Eastern Finland) for providing resources to carry out this study. Biobank personnel who have collected the data and processed the samples: Sabrina Belgasem (Helsinki Biobank), Henna Palin, Minttu Virolainen and Anna-Kaisa Pohjonen (Finnish Clinical Biobank Tampere), Anu Outinen-Tuuponen, Marja-Leena Kytökangas, Riikka-Mari Siiro-Virtanen (Arctic Biobank and Biobank Borealis of Northern Finland), Senni Lipponen (Central Finland Biobank), Nina Hurula (Biobank of Eastern Finland) and Heidi Kalve, Anniina Friman and biomedical laboratory scientist students from the Turku University of Applied Sciences' clinical laboratory. The participants of the TWINGEN study were recruited through THL Biobank (study number THLBB2022_83) and we thank all study participants for their generous participation in biobank research. We thank Jyrki Tammerluoto and Steffi Besselink for legal services and Huei-Yi Shen for administrative work. We thank the participants of the Finnish Twin Cohort study for their participation to TWINGEN and all previous data collection waves.

For peer review only - http://bmjopen.bmj.com/site/about/guidelines.xhtml

Funding statement

TWINGEN study was funded by the FinnGen project with the aim to enrich the phenotype information in FinnGen to achieve the goals of the project. Finnish Twin Cohort study has been supported by the Academy of Finland (Grants 265240, 263278, 308248), the Sigrid Jusélius Foundation, NIH/NHLBI grant HL104125 and NIH Grant R01 AG060470. EV was supported by the Academy of Finland (grants 314639 and 345988). JK was supported by Centre of Excellence in Complex Disease Genetics (grants #312073 and #336823 from the Academy of Finland).

The FinnGen project is funded by Business Finland and AbbVie, AstraZeneca UK, Biogen, Bristol Myers Squibb (and Celgene Corporation & Celgene International II), Genentech, Merck Sharp & Dohme LLC, a subsidiary of Merck & Co., Inc., Rahway, NJ, USA, Pfizer, GlaxoSmithKline Intellectual Property Development, Sanofi US Services, Maze Therapeutics, Janssen Biotech, Novartis, and Boehringer Ingelheim. All Finnish biobanks are members of the BBMRI.fi infrastructure (https://www.bbmri.fi). The FINBB (https://finbb.fi/) is the coordinator of BBMRI-ERIC operations in Finland. The Finnish biobank data can be accessed through the Fingenious services (https://site.fingenious.fi/en/) managed by FINBB.

Competing interests statement

AP is the Chief Scientific Officer of the FinnGen project that is funded by thirteen pharmaceutical companies. HR was a full-time employee of Biogen during study planning and manuscript drafting and has stocks at Merck & Co. and Biogen Inc. MMF has received development funding from the Regional Council of Northern Savo and Business Finland for a data-driven tool related to memory disorders and healthcare decision tools, Charles River DRS Finland Ltd. and Orion Pharma have donated equipment for nonclinical cognition testing at the University of Eastern Finland. PI-M has received funding from Orion Research Foundation and Helsinki Biomedicum Foundation outside the present work. RK declares funding paid to the institution from Academy of Finland,

For peer review only

Government research funding, Saastamoinen Foundation, Vaajasalo foundation and Jane and Aatos Erkko foundation outside the present work; consulting fees from Orion Pharma; payment or honoraria from Angelini Pharma, Jazz Pharma, Lundbeck, Eisai, Orion Pharma, OmaMedical, Takeda, UCB; participation in monitoring or advisory board from Marinus Pharma and UCB; and leadership or fiduciary role in European Academy of Neurology Epilepsy scientific panel management group, European Epilepsy Reference network Epicare Steering Group and International League Against Epilepsy Career Development Commission. RS declares stocks or stock options at Orion Pharma. S-KH declares payment or honoraria and support for attending meetings or travel from Roche, consulting fees and participation in monitoring or advisory board from Novartis. TK declares payment or honoraria from Novartis Finland and Bayer Nordic SE. The authors declare no other competing financial or non-financial Interests.

Figure 1 legend: Flowchart of study participant selection

References

1. Nichols E, Szoeke CEI, Vollset SE, Abbasi N, Abd-Allah F, Abdela J, et al. Global, regional, and national burden of Alzheimer's disease and other dementias, 1990–2016: a systematic analysis for the Global Burden of Disease Study 2016. Lancet Neurol. 2019 Jan;18(1):88–106.

2. Prince M, Bryce R, Albanese E, Wimo A, Ribeiro W, Ferri CP. The global prevalence of dementia: A systematic review and metaanalysis. Alzheimers Dement. 2013 Jan;9(1):63-75.e2.

3. Official Statistics of Finland (OSF). Causes of death [online publication]. ISSN=1799-5078. [Internet]. Helsinki: Statistics Finland; 2023 [cited 2023 Sep 29]. Available from: https://www.stat.fi/en/statistics/ksyyt

4. Malzbender K, Lavin-Mena L, Hughes L, Bose N, Goldman D, Patel D. Key Barriers to Clinical Trials for Alzheimer's Disease [Internet]. USC Schaeffer Center for Health Policy and Economics.; 2020. Available from: https://healthpolicy.usc.edu/research/key-barriers-for-clinical-trialsfor- alzheimers-disease/

5. Jack CR, Bennett DA, Blennow K, Carrillo MC, Dunn B, Haeberlein SB, et al. NIA-AA Research Framework: Toward a biological definition of Alzheimer's disease. Alzheimers Dement. 2018 Apr 1;14(4):535–62.

For peer review only

6. Hansson O, Edelmayer RM, Boxer AL, Carrillo MC, Mielke MM, Rabinovici GD, et al. The Alzheimer's Association appropriate use recommendations for blood biomarkers in Alzheimer's disease. Alzheimers Dement. 2022 Dec;18(12):2669–86.

7. Ferreira PCL, Zhang Y, Snitz B, Chang CH, Bellaver B, Jacobsen E, et al. Plasma biomarkers identify older adults at risk of Alzheimer's disease and related dementias in a real-world population-based cohort. Alzheimers Dement. 2023 Mar 6;alz.12986.

8. Kurki MI, Karjalainen J, Palta P, Sipilä TP, Kristiansson K, Donner KM, et al. FinnGen provides genetic insights from a well-phenotyped isolated population. Nature. 2023 Jan 19;613(7944):508–18.

9. Julkunen V, Schwarz C, Kalapudas J, Hallikainen M, Piironen AK, Mannermaa A, et al. A FinnGen pilot clinical recall study for Alzheimer's disease. Sci Rep. 2023 Aug 3;13(1):12641.

10. Langbaum JB, Zissimopoulos J, Au R, Bose N, Edgar CJ, Ehrenberg E, et al. Recommendations to address key recruitment challenges of Alzheimer's disease clinical trials. Alzheimers Dement. 2023 Feb;19(2):696–707.

11. Zetterberg H, Blennow K. Blood Biomarkers: Democratizing Alzheimer's Diagnostics. Neuron. 2020 Jun;106(6):881–3.

12. Husain M. Screening for Alzheimer's disease: plasma biomarkers and what else? Brain. 2023 Feb 13;146(2):407–8.

13. Rodosthenous RS, Niemi MEK, Kallio L, Perala M, Terho P, Knopp T, et al. Recontacting biobank participants to collect lifestyle, behavioural and cognitive information via online questionnaires: lessons from a pilot study within FinnGen. BMJ Open. 2022 Oct;12(10):e064695.

14. Kaprio J, Bollepalli S, Buchwald J, Iso-Markku P, Korhonen T, Kovanen V, et al. The Older Finnish Twin Cohort — 45 Years of Follow-up. Twin Res Hum Genet. 2019 Aug;22(4):240–54.

15. Tuomela J, Kaprio J, Sipilä PN, Silventoinen K, Wang X, Ollikainen M, et al. Accuracy of self-reported anthropometric measures — Findings from the Finnish Twin Study. Obes Res Clin Pract. 2019 Nov;13(6):522–8.

16. Lindgren N, Rinne JO, Palviainen T, Kaprio J, Vuoksimaa E. Prevalence and correlates of dementia and mild cognitive impairment classified with different versions of the modified Telephone Interview for Cognitive Status (TICS-m). Int J Geriatr Psychiatry. 2019 Dec;34(12):1883–91.

17. Huang Y, Ollikainen M, Muniandy M, Zhang T, van Dongen J, Hao G, et al. Supplementary Materials: Identification, Heritability, and Relation With Gene Expression of Novel DNA Methylation Loci for Blood Pressure. Hypertension. 2020 Jul;76(1):195–205.

18. Gatz M, Reynolds C, Nikolic J, Lowe B, Karel M, Pedersen N. An Empirical Test of Telephone Screening to Identify Potential Dementia Cases. Int Psychogeriatr. 1995 Sep;7(3):429–38.

For peer review only - http://bmjopen.bmj.com/site/about/guidelines.xhtml

19. Brandt J, Spencer M, Folstein M. The Telephone Interview for Cognitive Status. Neuropsychiatry, Neuropsychology, and Behavioural Neurology; 1988.

20. Järvenpää T, Rinne JO, Räihä I, Koskenvuo M, Löppönen M, Hinkka S, et al. Characteristics of Two Telephone Screens for Cognitive Impairment. Dement Geriatr Cogn Disord. 2002;13(3):149–55.

21. Lindgren N, Kaprio J, Rinne JO, Vuoksimaa E. Immediate verbal recall and familial dementia risk: population-based study of over 4000 twins. J Neurol Neurosurg Psychiatry. 2019 Jan;90(1):90–7.

22. Rhodius-Meester HFM, Paajanen T, Koikkalainen J, Mahdiani S, Bruun M, Baroni M, et al. cCOG: A web-based cognitive test tool for detecting neurodegenerative disorders. Alzheimers Dement Diagn Assess Dis Monit [Internet]. 2020 Jan [cited 2023 Jan 5];12(1). Available from: https://onlinelibrary.wiley.com/doi/10.1002/dad2.12083

23. Van Gils AM, Van De Beek M, Van Unnik AAJM, Tolonen A, Handgraaf D, Van Leeuwenstijn M, et al. Optimizing cCOG, a Web-based tool, to detect dementia with Lewy Bodies. Alzheimers Dement Diagn Assess Dis Monit. 2022 Jan;14(1):e12379.

24. Morris JC, Heyman A, Mohs RC, Hughes JP, van Belle G, Fillenbaum G, et al. The Consortium to Establish a Registry for Alzheimer's Disease (CERAD): Part I. Clinical and neuropsychological assessment of Alzheimer's disease. Neurology [Internet]. 1989 [cited 2023 Feb 2]; Available from: https://www.neurology.org/lookup/doi/10.1212/WNL.44.7.1253

25. Hazan E, Frankenburg F, Brenkel M, Shulman K. The test of time: a history of clock drawing. Int J Geriatr Psychiatry [Internet]. 2018 Jan [cited 2023 Oct 17];33(1). Available from: https://onlinelibrary.wiley.com/doi/10.1002/gps.4731

26. Hallikainen I, Alenius M, Hokkanen L, Karrasch M, Krüger J, Ngandu T, et al. CERAD-tehtäväsarjaan koulutustason huomioivat katkaisurajat ja kokonaispistemäärä käyttöön. Suom Lääkärilehti. 2023;78(e35809).

27. Weschler D. WMS-III - Wechsler Memory Scale - 3rd ed. The Psychological Corporation, New York : Wechsler, D. Psykologien Kustannus Oy, Helsinki; 2008.

28. Armitage SG. An Analysis of Certain Psychological Tests Used for the Evaluation of Brain Injury. Psychological monographs; 1946.

29. Stroop JR. Studies of inference in serial verbal reactions. J Exp Psychol. 1935;18:643–62.

30. Ngandu T, Lehtisalo J, Solomon A, Levälahti E, Ahtiluoto S, Antikainen R, et al. A 2 year multidomain intervention of diet, exercise, cognitive training, and vascular risk monitoring versus control to prevent cognitive decline in at-risk elderly people (FINGER): a randomised controlled trial. The Lancet. 2015 Jun;385(9984):2255–63.

31. Karikari TK, Pascoal TA, Ashton NJ, Janelidze S, Benedet AL, Rodriguez JL, et al. Blood phosphorylated tau 181 as a biomarker for Alzheimer's disease: a diagnostic performance and prediction modelling study using data from four prospective cohorts. Lancet Neurol. 2020 May;19(5):422–33.

For peer review only

32. Thijssen EH, Verberk IMW, Kindermans J, Abramian A, Vanbrabant J, Ball AJ, et al. Differential diagnostic performance of a panel of plasma biomarkers for different types of dementia. Alzheimers Dement Diagn Assess Dis Monit. 2022 Jan;14(1):e12285.

33. Ashton NJ, Brum WS, Di Molfetta G, Benedet AL, Arslan B, Jonatis E, et al. Diagnostic accuracy of the plasma ALZpath pTau217 immunoassay to identify Alzheimer's disease pathology [Internet]. Neurology; 2023 Jul [cited 2023 Aug 30]. Available from: http://medrxiv.org/lookup/doi/10.1101/2023.07.11.23292493

34. Vuoksimaa E, Palviainen T, Lindgren N, Rinne JO, Kaprio J. Accuracy of Imputation for Apolipoprotein E ε Alleles in Genome-Wide Genotyping Data. JAMA Netw Open. 2020 Jan 24;3(1):e1919960.

35. Bellenguez C. New insights into the genetic etiology of Alzheimer's disease and related dementias. Nat Genet. 2022;54.

36. Livingston G, Huntley J, Sommerlad A, Ames D, Ballard C, Banerjee S, et al. Dementia prevention, intervention, and care: 2020 report of the Lancet Commission. The Lancet. 2020 Aug;396(10248):413–46.

37. Husu P, Tokola K, Vähä-Ypyä H, Sievänen H, Suni J, Heinonen OJ, et al. Physical Activity, Sedentary Behavior, and Time in Bed Among Finnish Adults Measured 24/7 by Triaxial Accelerometry. J Meas Phys Behav. 2021 Jun 1;4(2):163–73.

38. Matthews CE, Hagströmer M, Pober DM, Bowles HR. Best Practices for Using Physical Activity Monitors in Population-Based Research. Med Sci Sports Exerc. 2012 Jan;44(1S):S68–76.

39. Husu P, Suni J, Vähä-Ypyä H, Sievänen H, Tokola K, Valkeinen H, et al. Objectively measured sedentary behavior and physical activity in a sample of Finnish adults: a cross-sectional study. BMC Public Health. 2016 Dec;16(1):920.

40. Vähä-Ypyä H, Husu P, Suni J, Vasankari T, Sievänen H. Reliable recognition of lying, sitting, and standing with a hip-worn accelerometer. Scand J Med Sci Sports. 2018 Mar;28(3):1092–102.

41. Vähä-Ypyä H, Vasankari T, Husu P, Suni J, Sievänen H. A universal, accurate intensity-based classification of different physical activities using raw data of accelerometer. Clin Physiol Funct Imaging. 2015 Jan;35(1):64–70.

42. Vähä-Ypyä H, Vasankari T, Husu P, Mänttäri A, Vuorimaa T, Suni J, et al. Validation of Cut-Points for Evaluating the Intensity of Physical Activity with Accelerometry-Based Mean Amplitude Deviation (MAD). Miller PJO, editor. PLOS ONE. 2015 Aug 20;10(8):e0134813.

43. Altini M, Kinnunen H. The Promise of Sleep: A Multi-Sensor Approach for Accurate Sleep Stage Detection Using the Oura Ring. Sensors. 2021 Jun 23;21(13):4302.

44. Henriksen A, Svartdal F, Grimsgaard S, Hartvigsen G, Hopstock LA. Polar Vantage and Oura Physical Activity and Sleep Trackers: Validation and Comparison Study. JMIR Form Res. 2022 May 27;6(5):e27248.

For peer review only

45. Radloff LS. The CES-D Scale: A Self-Report Depression Scale for Research in the General Population. Appl Psychol Meas. 1977 Jun;1(3):385–401.

46. Saari TT, Piirtola M, Aaltonen A, Palviainen T, Varjonen A, Julkunen V, et al. Self-reported depression symptoms in old age across cohorts and cognitive spectrum and associations with genetic risk [Internet]. PsyArXiv; 2023 May [cited 2023 Sep 29]. Available from: https://osf.io/jvmtz

47. Ryff CD. Psychological Well-Being Revisited: Advances in the Science and Practice of Eudaimonia. Psychother Psychosom. 2014;83(1):10–28.

48. Ryff CD. Happiness Is Everything, or Is It? Explorations on the Meaning of Psychological Well-Being. J Pers Soc Psychol. 1989;57(6):1069–801.

49. Floderus B. Psycho-social factors in relation to coronary heart disease and associated risk factors. Nord Hyg Tidskr. 1974;(Suppl. 6).

50. Rose RJ, Koskenvuo M, Kaprio J, Sarna S, Langinvainio H. Shared Genes, Shared Experiences,and Similarity of Personality: Data From 14,288 Adult Finnish Co-Twins. J Pers Soc Psychol. 1988;54(1):161–71.

51. Allardt E. About dimensions of welfare: Explanatory analysis of the comparative Scandinavian survey. Research Group of Comparative Sociology. Helsinki: University of Helsinki; 1973. (Research Reports).

52. Koivumaa-Honkanen H, Kaprio J, Honkanen R, Viinam�ki H, Koskenvuo M. Life satisfaction and depression in a 15-year follow-up of healthy adults. Soc Psychiatry Psychiatr Epidemiol. 2004 Dec;39(12):994–9.

53. Ashton NJ, Brum WS, Di Molfetta G, Benedet AL, Arslan B, Jonaitis E, et al. Diagnostic Accuracy of a Plasma Phosphorylated Tau 217 Immunoassay for Alzheimer Disease Pathology. JAMA Neurol [Internet]. 2024 Jan 22 [cited 2024 Feb 16]; Available from: https://jamanetwork.com/journals/jamaneurology/fullarticle/2813751

54. Bondi MW, Edmonds EC, Jak AJ, Clark LR, Delano-Wood L, McDonald CR, et al. Neuropsychological Criteria for Mild Cognitive Impairment Improves Diagnostic Precision, Biomarker Associations, and Progression Rates. J Alzheimers Dis. 2014 Aug 11;42(1):275–89.

55. Vuoksimaa E, McEvoy LK, Holland D, Franz CE, Kremen WS, for the Alzheimer's Disease Neuroimaging Initiative. Modifying the minimum criteria for diagnosing amnestic MCI to improve prediction of brain atrophy and progression to Alzheimer's disease. Brain Imaging Behav. 2020 Jun;14(3):787–96.

56. Park LQ, Gross AL, McLaren DG, Pa J, Johnson JK, Mitchell M, et al. Confirmatory factor analysis of the ADNI neuropsychological battery. Brain Imaging Behav. 2012 Dec;6(4):528–39.

57. Dowling NM, Hermann B, La Rue A, Sager MA. Latent structure and factorial invariance of a neuropsychological test battery for the study of preclinical Alzheimer's disease. Neuropsychology. 2010 Nov;24(6):742–56.

For peer review only - http://bmjopen.bmj.com/site/about/guidelines.xhtml

58. Varjonen A, Schwarz C, Vuoksimaa E. Co-twin design in brain imaging—review on biomarkers of Alzheimer's disease. Cereb Cortex. 2023 Jul 5;33(14):9054–66.

For peer review only

FinnGen Collaborators

Full Name	Affiliation	E-mail	Role 1	Role 2
Aarno Palotie	Institute for Molecular Medicine Finland (FIMM), HiLIFE, University of Helsinki, Helsinki, Finland; Broad Institute of MIT and Harvard; Massachusetts General Hospital	aarno.palotie@helsinki.fi	Steering Committee	Steering Committee
Mark Daly	Institute for Molecular Medicine Finland (FIMM), HiLIFE, University of Helsinki, Helsinki, Finland; Broad Institute of MIT and Harvard; Massachusetts General Hospital	mark.daly@helsinki.fi	Steering Committee	Steering Committee
Bridget Riley-Gills	Abbvie, Chicago, IL, United States	bridget.rileygillis@abbvie.com	Steering Committee	Pharmaceutical companies
Howard Jacob	Abbvie, Chicago, IL, United States	howard.jacob@abbvie.com	Steering Committee	Pharmaceutical companies
Dirk Paul	Astra Zeneca, Cambridge, United Kingdom	dirk.paul@astrazeneca.com	Steering Committee	Pharmaceutical companies
Athena Matakidou	Astra Zeneca, Cambridge, United Kingdom	athena.x.matakidou@gsk.com	Steering Committee	Pharmaceutical companies

For peer review only

For peer review only - http://bmjopen.bmj.com/site/about/guidelines.xhtml

Adam Platt	Astra Zeneca, Cambridge, United Kingdom	adam.platt@astrazeneca.com	Steering Committee	Pharmaceutical companies
Heiko Runz	Biogen, Cambridge, MA, United States	heiko.runz@biogen.com	Steering Committee	Pharmaceutical companies
Sally John	Biogen, Cambridge, MA, United States	sally.john@biogen.com	Steering Committee	Pharmaceutical companies
George Okafo	Boehringer Ingelheim, Ingelheim am Rhein, Germany	george.okafo@boehringer-ingelheim.com	Steering Committee	Pharmaceutical companies
Nathan Lawless	Boehringer Ingelheim, Ingelheim am Rhein, Germany	nathan.lawless@boehringer-ingelheim.com	Steering Committee	Pharmaceutical companies
Heli Salminen-Mankonen	Boehringer Ingelheim, Ingelheim am Rhein, Germany	heli.salminen-mankonen@boehringer-ingelheim.com	Steering Committee	Pharmaceutical companies
Robert Plenge	Bristol Myers Squibb, New York, NY, United States	robert.plenge@bms.com	Steering Committee	Pharmaceutical companies
Joseph Maranville	Bristol Myers Squibb, New York, NY, United States	joseph.maranville@bms.com	Steering Committee	Pharmaceutical companies
Mark McCarthy	Genentech, San Francisco, CA, United States	mccarthy.mark@gene.com	Steering Committee	Pharmaceutical companies

For peer review only

Julie Hunkapiller	Genentech, San Francisco, CA, United States	hunkapiller.julie@gene.com	Steering Committee	Pharmaceutical companies
Margaret G. Ehm	GlaxoSmithKline, Collegeville, PA, United States	meg.g.ehm@gsk.com	Steering Committee	Pharmaceutical companies
Kirsi Auro	GlaxoSmithKline, Espoo, Finland	kirsi.m.auro@gsk.com	Steering Committee	Pharmaceutical companies
Simonne Longerich	Merck, Kenilworth, NJ, United States	simonne.longerich@merck.com	Steering Committee	Pharmaceutical companies
Caroline Fox	Merck, Kenilworth, NJ, United States	caroline.fox@merck.com	Steering Committee	Pharmaceutical companies
Anders Mälarstig	Pfizer, New York, NY, United States	anders.malarstig@pfizer.com	Steering Committee	Pharmaceutical companies
Katherine Klinger	Translational Sciences, Sanofi R&D, Framingham, MA, USA	katherine.klinger@sanofi.com	Steering Committee	Pharmaceutical companies
Deepak Raipal	Translational Sciences, Sanofi R&D, Framingham, MA, USA	deepak.rajpal@sanofi.com	Steering Committee	Pharmaceutical companies
Eric Green	Maze Therapeutics, San Francisco, CA, United States	egreen@mazetx.com	Steering Committee	Pharmaceutical companies
Robert Graham	Maze Therapeutics, San Francisco, CA, United States	rgraham@mazetx.com	Steering Committee	Pharmaceutical companies
Robert Yang	Janssen Biotech, Beerse, Belgium	ryang31@its.jnj.com	Steering Committee	Pharmaceutical companies

For peer review only - http://bmjopen.bmj.com/site/about/guidelines.xhtml

Chris O´Donnell	Novartis Institutes for BioMedical Research, Cambridge, MA, United States	chris.odonnell@novartis.com	Steering Committee	Pharmaceutical companies
Tomi P. Mäkelä	HiLIFE, University of Helsinki, Finland, Finland	tomi.makela@helsinki.fi	Steering Committee	University of Helsinki & Biobanks
Jaakko Kaprio	Institute for Molecular Medicine Finland (FIMM), HiLIFE, University of Helsinki, Helsinki, Finland	jaakko.kaprio@helsinki.fi	Steering Committee	University of Helsinki & Biobanks
Petri Virolainen	Auria Biobank / University of Turku / Hospital District of Southwest Finland, Turku, Finland	petri.virolainen@tyks.fi	Steering Committee	University of Helsinki & Biobanks
Antti Hakanen	Auria Biobank / University of Turku / Hospital District of Southwest Finland, Turku, Finland	antti.hakanen@tyks.fi	Steering Committee	University of Helsinki & Biobanks
Terhi Kilpi	THL Biobank / Finnish Institute for Health and Welfare (THL), Helsinki, Finland	terhi.kilpi@thl.fi	Steering Committee	University of Helsinki & Biobanks
Markus Perola	THL Biobank / Finnish Institute for Health and Welfare (THL), Helsinki, Finland	markus.perola@thl.fi	Steering Committee	University of Helsinki & Biobanks

For peer review only - http://bmjopen.bmj.com/site/about/guidelines.xhtml

Jukka Partanen	Finnish Red Cross Blood Service / Finnish Hematology Registry and Clinical Biobank, Helsinki, Finland	jukka.partanen@veripalvelu.fi	Steering Committee	University of Helsinki & Biobanks
Anne Pitkäranta	Helsinki Biobank / Helsinki University and Hospital District of Helsinki and Uusimaa, Helsinki	anne.pitkaranta@hus.fi	Steering Committee	University of Helsinki & Biobanks
Taneli Raivio	Helsinki Biobank / Helsinki University and Hospital District of Helsinki and Uusimaa, Helsinki	taneli.raivio@hus.fi	Steering Committee	University of Helsinki & Biobanks
Jani Tikkanen	Northern Finland Biobank Borealis / University of Oulu / Wellbeing Services County of North Ostrobothnia, Oulu, Finland	jani.tikkanen@pohde.fi	Steering Committee	University of Helsinki & Biobanks
Raisa Serpi	Northern Finland Biobank Borealis / University of Oulu / Wellbeing Services County of North Ostrobothnia, Oulu, Finland	raisa.serpi@ppshp.fi	Steering Committee	University of Helsinki & Biobanks
Tarja Laitinen	Finnish Clinical Biobank Tampere / University of Tampere / The Wellbeing Services County of Pirkanmaa, Tampere, Finland	tarja.laitinen@pirha.fi	Steering Committee	University of Helsinki & Biobanks

For peer review only - http://bmjopen.bmj.com/site/about/guidelines.xhtml

Veli-Matti Kosma	Biobank of Eastern Finland / University of Eastern Finland / Northern Savo Hospital District, Kuopio, Finland	veli-matti.kosma@uef.fi	Steering Committee	University of Helsinki & Biobanks
Jari Laukkanen	Central Finland Biobank / University of Jyväskylä / Central Finland Health Care District, Jyväskylä, Finland	jari.laukkanen@ksshp.fi	Steering Committee	University of Helsinki & Biobanks
Marco Hautalahti	FINBB - Finnish biobank cooperative	marco.hautalahti@finbb.fi	Steering Committee	University of Helsinki & Biobanks
Outi Tuovila	Business Finland, Helsinki, Finland	outi.tuovila@businessfinland.fi	Steering Committee	Other Experts/ Non-Voting Members
Raimo Pakkanen	Business Finland, Helsinki, Finland	raimo.pakkanen@businessfinland.fi	Steering Committee	Other Experts/ Non-Voting Members
Jeffrey Waring	Abbvie, Chicago, IL, United States	jeff.waring@abbvie.com	Scientific Committee	Pharmaceutical companies
Bridget Riley-Gillis	Abbvie, Chicago, IL, United States	bridget.rileygillis@abbvie.com	Scientific Committee	Pharmaceutical companies
Fedik Rahimov	Abbvie, Chicago, IL, United States	fedik.rahimov@abbvie.com	Scientific Committee	Pharmaceutical companies

For peer review only - http://bmjopen.bmj.com/site/about/guidelines.xhtml

Ioanna Tachmazidou	Astra Zeneca, Cambridge, United Kingdom	ioanna.tachmazidou@astrazeneca.com	Scientific Committee	Pharmaceutical companies
Chia-Yen Chen	Biogen, Cambridge, MA, United States	chiayen.chen@biogen.com	Scientific Committee	Pharmaceutical companies
Heiko Runz	Biogen, Cambridge, MA, United States	heiko.runz@biogen.com	Scientific Committee	Pharmaceutical companies
Zhihao Ding	Boehringer Ingelheim, Ingelheim am Rhein, Germany	zhihao.ding@boehringer-ingelheim.com	Scientific Committee	Pharmaceutical companies
Marc Jung	Boehringer Ingelheim, Ingelheim am Rhein, Germany	marc_oliver.jung@boehringer-ingelheim.com	Scientific Committee	Pharmaceutical companies
Shameek Biswas	Bristol Myers Squibb, New York, NY, United States	Shameek.Biswas@bms.com	Scientific Committee	Pharmaceutical companies
Rion Pendergrass	Genentech, San Francisco, CA, United States	penders2@gene.com	Scientific Committee	Pharmaceutical companies
Julie Hunkapiller	Genentech, San Francisco, CA, United States	hunkapiller.julie@gene.com	Scientific Committee	Pharmaceutical companies
Margaret G. Ehm	GlaxoSmithKline, Collegeville, PA, United States	meg.g.ehm@gsk.com	Scientific Committee	Pharmaceutical companies
David Pulford	GlaxoSmithKline, Stevenage, United Kingdom	david.x.pulford@gsk.com	Scientific Committee	Pharmaceutical companies

For peer review only
For peer review only - http://bmjopen.bmj.com/site/about/guidelines.xhtml

Neha Raghavan	Merck, Kenilworth, NJ, United States	neha.raghavan@merck.com	Scientific Committee	Pharmaceutical companies
Adriana Huertas-Vazquez	Merck, Kenilworth, NJ, United States	adriana.huertas.vazquez@merck.com	Scientific Committee	Pharmaceutical companies
Jae-Hoon Sul	Merck, Kenilworth, NJ, United States	jae.hoon.sul@merck.com	Scientific Committee	Pharmaceutical companies
Anders Mälarstig	Pfizer, New York, NY, United States	anders.malarstig@pfizer.com	Scientific Committee	Pharmaceutical companies
Xinli Hu	Pfizer, New York, NY, United States	xinli.hu@pfizer.com	Scientific Committee	Pharmaceutical companies
Katherine Klinger	Translational Sciences, Sanofi R&D, Framingham, MA, USA	katherine.klinger@sanofi.com	Scientific Committee	Pharmaceutical companies
Robert Graham	Maze Therapeutics, San Francisco, CA, United States	rgraham@mazetx.com	Scientific Committee	Pharmaceutical companies
Eric Green	Maze Therapeutics, San Francisco, CA, United States	egreen@mazetx.com	Scientific Committee	Pharmaceutical companies
Sahar Mozaffari	Maze Therapeutics, San Francisco, CA, United States	smozaffari@mazetx.com	Scientific Committee	Pharmaceutical companies
Dawn Waterworth	Janssen Research & Development, LLC, Spring House, PA, United States	dwaterwo@its.jnj.com	Scientific Committee	Pharmaceutical companies

For peer review only

For peer review only - http://bmjopen.bmj.com/site/about/guidelines.xhtml

Nicole Renaud	Novartis Institutes for BioMedical Research, Cambridge, MA, United States	nicole.renaud@novartis.com	Scientific Committee	Pharmaceutical companies
Ma´en Obeidat	Novartis Institutes for BioMedical Research, Cambridge, MA, United States	maen.obeidat@novartis.com	Scientific Committee	Pharmaceutical companies
Samuli Ripatti	Institute for Molecular Medicine Finland (FIMM), HiLIFE, University of Helsinki, Helsinki, Finland	samuli.ripatti@helsinki.fi	Scientific Committee	University of Helsinki & Biobanks
Johanna Schleutker	Auria Biobank / Univ. of Turku / Hospital District of Southwest Finland, Turku, Finland	johanna.schleutker@utu.fi	Scientific Committee	University of Helsinki & Biobanks
Markus Perola	THL Biobank / Finnish Institute for Health and Welfare (THL), Helsinki, Finland	markus.perola@thl.fi	Scientific Committee	University of Helsinki & Biobanks
Mikko Arvas	Finnish Red Cross Blood Service / Finnish Hematology Registry and Clinical Biobank, Helsinki, Finland	mikko.arvas@veripalvelu.fi	Scientific Committee	University of Helsinki & Biobanks
Olli Carpén	Helsinki Biobank / Helsinki University and Hospital District of Helsinki and Uusimaa, Helsinki	olli.carpen@helsinki.fi	Scientific Committee	University of Helsinki & Biobanks

For peer review only

Reetta Hinttala	Northern Finland Biobank Borealis / University of Oulu / Northern Ostrobothnia Hospital District, Oulu, Finland	reetta.hinttala@oulu.fi	Scientific Committee	University of Helsinki & Biobanks
Johannes Kettunen	Northern Finland Biobank Borealis / University of Oulu / Northern Ostrobothnia Hospital District, Oulu, Finland	johannes.kettunen@oulu.fi	Scientific Committee	University of Helsinki & Biobanks
Arto Mannermaa	Biobank of Eastern Finland / University of Eastern Finland / Northern Savo Hospital District, Kuopio, Finland	arto.mannermaa@uef.fi	Scientific Committee	University of Helsinki & Biobanks
Katriina Aalto-Setälä	Faculty of Medicine and Health Technology, Tampere University, Tampere, Finland	katriina.aalto-setala@tuni.fi	Scientific Committee	University of Helsinki & Biobanks
Mika Kähönen	Finnish Clinical Biobank Tampere / University of Tampere / Pirkanmaa Hospital District, Tampere, Finland	mika.kahonen@uta.fi	Scientific Committee	University of Helsinki & Biobanks
Jari Laukkanen	Central Finland Biobank / University of Jyväskylä / Central Finland Health Care District, Jyväskylä, Finland	jari.laukkanen@ksshp.fi	Scientific Committee	University of Helsinki & Biobanks

For peer review only

Johanna Mäkelä	FINBB - Finnish biobank cooperative	johanna.makela@finbb.fi	Scientific Committee	University of Helsinki & Biobanks
Reetta Kälviäinen	Northern Savo Hospital District, Kuopio, Finland	reetta.kalviainen@kuh.fi	Clinical Groups	Neurology Group
Valtteri Julkunen	Northern Savo Hospital District, Kuopio, Finland	valtteri.julkunen@kuh.fi	Clinical Groups	Neurology Group
Hilkka Soininen	Northern Savo Hospital District, Kuopio, Finland	hilkka.soininen@uef.fi	Clinical Groups	Neurology Group
Anne Remes	Clinical Neurosciences, University of Helsinki, Helsinki, Finland	anne.remes@oulu.fi	Clinical Groups	Neurology Group
Mikko Hiltunen	University of Eastern Finland, Kuopio, Finland	mikko.hiltunen@uef.fi	Clinical Groups	Neurology Group
Jukka Peltola	Pirkanmaa Hospital District, Tampere, Finland	jukka.peltola@pshp.fi	Clinical Groups	Neurology Group
Minna Raivio	Hospital District of Helsinki and Uusimaa, Helsinki, Finland	minna.raivio@geri.fi	Clinical Groups	Neurology Group
Pentti Tienari	Hospital District of Helsinki and Uusimaa, Helsinki, Finland	pentti.tienari@hus.fi	Clinical Groups	Neurology Group
Juha Rinne	Hospital District of Southwest Finland, Turku, Finland	juha.rinne@tyks.fi	Clinical Groups	Neurology Group

For peer review only - http://bmjopen.bmj.com/site/about/guidelines.xhtml

Roosa Kallionpää	Hospital District of Southwest Finland, Turku, Finland	roosa.kallionpaa@tyks.fi	Clinical Groups	Neurology Group
Juulia Partanen	Institute for Molecular Medicine Finland, HiLIFE, University of Helsinki, Finland	juulia.partanen@helsinki.fi	Clinical Groups	Neurology Group
Ali Abbasi	Abbvie, Chicago, IL, United States	ali.abbasi@abbvie.com	Clinical Groups	Neurology Group
Adam Ziemann	Abbvie, Chicago, IL, United States	adam.ziemann@abbvie.com	Clinical Groups	Neurology Group
Nizar Smaoui	Abbvie, Chicago, IL, United States	nizar.smaoui@abbvie.com	Clinical Groups	Neurology Group
Anne Lehtonen	Abbvie, Chicago, IL, United States	anne.lehtonen@abbvie.com	Clinical Groups	Neurology Group
Susan Eaton	Biogen, Cambridge, MA, United States	susan.eaton@biogen.com	Clinical Groups	Neurology Group
Heiko Runz	Biogen, Cambridge, MA, United States	heiko.runz@biogen.com	Clinical Groups	Neurology Group
Sanni Lahdenperä	Biogen, Cambridge, MA, United States	sanni.lahdenpera@biogen.com	Clinical Groups	Neurology Group
Shameek Biswas	Bristol Myers Squibb, New York, NY, United States	shameek.biswas@bms.com	Clinical Groups	Neurology Group

For peer review only - http://bmjopen.bmj.com/site/about/guidelines.xhtml

Julie Hunkapiller	Genentech, San Francisco, CA, United States	hunkapiller.julie@gene.com	Clinical Groups	Neurology Group
Natalie Bowers	Genentech, San Francisco, CA, United States	bowersn1@gene.com	Clinical Groups	Neurology Group
Edmond Teng	Genentech, San Francisco, CA, United States	teng.edmond@gene.com	Clinical Groups	Neurology Group
Rion Pendergrass	Genentech, San Francisco, CA, United States	penders2@gene.com	Clinical Groups	Neurology Group
Fanli Xu	GlaxoSmithKline, Brentford, United Kingdom	chun-fang.2.xu@gsk.com	Clinical Groups	Neurology Group
David Pulford	GlaxoSmithKline, Stevenage, United Kingdom	david.x.pulford@gsk.com	Clinical Groups	Neurology Group
Kirsi Auro	GlaxoSmithKline, Espoo, Finland	kirsi.m.auro@gsk.com	Clinical Groups	Neurology Group
Laura Addis	GlaxoSmithKline, Brentford, United Kingdom	laura.x.addis@gsk.com	Clinical Groups	Neurology Group
John Eicher	GlaxoSmithKline, Brentford, United Kingdom	john.d.eicher@gsk.com	Clinical Groups	Neurology Group
Qingqin S Li	Janssen Research & Development, LLC, Titusville, NJ 08560, United States	QLi2@its.jnj.com	Clinical Groups	Neurology Group

For peer review only - http://bmjopen.bmj.com/site/about/guidelines.xhtml

Karen He	Janssen Research & Development, LLC, Spring House, PA, United States	khe2@its.jnj.com	Clinical Groups	Neurology Group
Ekaterina Khramtsova	Janssen Research & Development, LLC, Spring House, PA, United States	ekhramts@its.jnj.com	Clinical Groups	Neurology Group
Neha Raghavan	Merck, Kenilworth, NJ, United States	neha.raghavan@merck.com	Clinical Groups	Neurology Group
Martti Färkkilä	Hospital District of Helsinki and Uusimaa, Helsinki, Finland	martti.farkkila@hus.fi	Clinical Groups	Gastroenterology Group
Jukka Koskela	Hospital District of Helsinki and Uusimaa, Helsinki, Finland	jukka.koskela@helsinki.fi	Clinical Groups	Gastroenterology Group
Sampsa Pikkarainen	Hospital District of Helsinki and Uusimaa, Helsinki, Finland	sampsa.pikkarainen@hus.fi	Clinical Groups	Gastroenterology Group
Airi Jussila	Pirkanmaa Hospital District, Tampere, Finland	airi.jussila@pshp.fi	Clinical Groups	Gastroenterology Group
Katri Kaukinen	Pirkanmaa Hospital District, Tampere, Finland	katri.kaukinen@tuni.fi	Clinical Groups	Gastroenterology Group
Timo Blomster	Wellbeing Services county of North Ostrobothnia, Oulu, Finland	timo.blomster@ppshp.fi	Clinical Groups	Gastroenterology Group
Mikko Kiviniemi	Northern Savo Hospital District, Kuopio, Finland	mikko.kiviniemi@kuh.fi	Clinical Groups	Gastroenterology Group

For peer review only

Markku Voutilainen	Hospital District of Southwest Finland, Turku, Finland	markku.voutilainen@tyks.fi	Clinical Groups	Gastroenterology Group
Mark Daly	Institute for Molecular Medicine, Finland (FIMM), HiLIFE, University of Helsinki, Helsinki, Finland; Broad Institute of MIT and Harvard; Massachusetts General Hospital	mark.daly@helsinki.fi	Clinical Groups	Gastroenterology Group
Ali Abbasi	Abbvie, Chicago, IL, United States	ali.abbasi@abbvie.com	Clinical Groups	Gastroenterology Group
Jeffrey Waring	Abbvie, Chicago, IL, United States	jeff.waring@abbvie.com	Clinical Groups	Gastroenterology Group
Nizar Smaoui	Abbvie, Chicago, IL, United States	nizar.smaoui@abbvie.com	Clinical Groups	Gastroenterology Group
Fedik Rahimov	Abbvie, Chicago, IL, United States	fedik.rahimov@abbvie.com	Clinical Groups	Gastroenterology Group
Anne Lehtonen	Abbvie, Chicago, IL, United States	anne.lehtonen@abbvie.com	Clinical Groups	Gastroenterology Group
Tim Lu	Genentech, San Francisco, CA, United States	lut8@gene.com	Clinical Groups	Gastroenterology Group
Natalie Bowers	Genentech, San Francisco, CA, United States	bowersn1@gene.com	Clinical Groups	Gastroenterology Group

For peer review only

Rion Pendergrass	Genentech, San Francisco, CA, United States	penders2@gene.com	Clinical Groups	Gastroenterology Group
Linda McCarthy	GlaxoSmithKline, Brentford, United Kingdom	linda.c.mccarthy@gsk.com	Clinical Groups	Gastroenterology Group
Amy Hart	Janssen Research & Development, LLC, Spring House, PA, United States	ahart13@its.jnj.com	Clinical Groups	Gastroenterology Group
Meijian Guan	Janssen Research & Development, LLC, Spring House, PA, United States	mguan4@its.jnj.com	Clinical Groups	Gastroenterology Group
Jason Miller	Merck, Kenilworth, NJ, United States	jason.miller4@merck.com	Clinical Groups	Gastroenterology Group
Kirsi Kalpala	Pfizer, New York, NY, United States	kirsi.kalpala@pfizer.com	Clinical Groups	Gastroenterology Group
Melissa Miller	Pfizer, New York, NY, United States	melissa.r.miller@pfizer.com	Clinical Groups	Gastroenterology Group
Xinli Hu	Pfizer, New York, NY, United States	xinli.hu@pfizer.com	Clinical Groups	Gastroenterology Group
Kari Eklund	Hospital District of Helsinki and Uusimaa, Helsinki, Finland	kari.eklund@hus.fi	Clinical Groups	Rheumatology Group
Antti Palomäki	Hospital District of Southwest Finland, Turku, Finland	ajpalo@utu.fi	Clinical Groups	Rheumatology Group

For peer review only - http://bmjopen.bmj.com/site/about/guidelines.xhtml

Pia Isomäki	Pirkanmaa Hospital District, Tampere, Finland	pia.isomaki@pshp.fi	Clinical Groups	Rheumatology Group
Laura Pirilä	Hospital District of Southwest Finland, Turku, Finland	laura.pirila@fimnet.fi,laura.pirila@tyks.fi	Clinical Groups	Rheumatology Group
Oili Kaipiainen-Seppänen	Northern Savo Hospital District, Kuopio, Finland	oili.kaipiainen-seppanen@kuh.fi	Clinical Groups	Rheumatology Group
Johanna Huhtakangas	Department of Internal Medicine, Kuopio University Hospital, Kuopio, Finland	johanna.huhtakangas@kuh.fi	Clinical Groups	Rheumatology Group
Nina Mars	Institute for Molecular Medicine Finland (FIMM), HiLIFE, University of Helsinki, Helsinki, Finland	nina.mars@helsinki.fi	Clinical Groups	Rheumatology Group
Ali Abbasi	Abbvie, Chicago, IL, United States	ali.abbasi@abbvie.com	Clinical Groups	Rheumatology Group
Jeffrey Waring	Abbvie, Chicago, IL, United States	jeff.waring@abbvie.com	Clinical Groups	Rheumatology Group
Fedik Rahimov	Abbvie, Chicago, IL, United States	fedik.rahimov@abbvie.com	Clinical Groups	Rheumatology Group
Apinya Lertratanakul	Abbvie, Chicago, IL, United States	apinya.lertratanakul@abbvie.com	Clinical Groups	Rheumatology Group

For peer review only

For peer review only - http://bmjopen.bmj.com/site/about/guidelines.xhtml

Nizar Smaoui	Abbvie, Chicago, IL, United States	nizar.smaoui@abbvie.com	Clinical Groups	Rheumatology Group
Anne Lehtonen	Abbvie, Chicago, IL, United States	anne.lehtonen@abbvie.com	Clinical Groups	Rheumatology Group
Marla Hochfeld	Bristol Myers Squibb, New York, NY, United States	mhochfeld@celgene.com	Clinical Groups	Rheumatology Group
Natalie Bowers	Genentech, San Francisco, CA, United States	bowersn1@gene.com	Clinical Groups	Rheumatology Group
Rion Pendergrass	Genentech, San Francisco, CA, United States	penders2@gene.com	Clinical Groups	Rheumatology Group
Jorge Esparza Gordillo	GlaxoSmithKline, Brentford, United Kingdom	jorge.x.esparza-gordillo@gsk.com	Clinical Groups	Rheumatology Group
Kirsi Auro	GlaxoSmithKline, Espoo, Finland	kirsi.m.auro@gsk.com	Clinical Groups	Rheumatology Group
Dawn Waterworth	Janssen Research & Development, LLC, Spring House, PA, United States	dwaterwo@its.jnj.com	Clinical Groups	Rheumatology Group
Fabiana Farias	Merck, Kenilworth, NJ, United States	fabiana.farias@merck.com	Clinical Groups	Rheumatology Group
Kirsi Kalpala	Pfizer, New York, NY, United States	kirsi.kalpala@pfizer.com	Clinical Groups	Rheumatology Group

50

For peer review only

Nan Bing	Pfizer, New York, NY, United States	nan.bing@pfizer.com	Clinical Groups	Rheumatology Group
Xinli Hu	Pfizer, New York, NY, United States	xinli.hu@pfizer.com	Clinical Groups	Rheumatology Group
Tarja Laitinen	The Wellbeing Services County of Pirkanmaa, Tampere, Finland	tarja.laitinen@pirha.fi	Clinical Groups	Pulmonology Group
Margit Pelkonen	Northern Savo Hospital District, Kuopio, Finland	margit.pelkonen@kuh.fi	Clinical Groups	Pulmonology Group
Paula Kauppi	Hospital District of Helsinki and Uusimaa, Helsinki, Finland	paula.kauppi@hus.fi	Clinical Groups	Pulmonology Group
Hannu Kankaanranta	University of Gothenburg, Gothenburg, Sweden/ Seinäjoki Central Hospital, Seinäjoki, Finland/ Tampere University, Tampere, Finland	hannu.kankaanranta@tuni.fi	Clinical Groups	Pulmonology Group
Terttu Harju	Northern Ostrobothnia Hospital District, Oulu, Finland	terttu.harju@oulu.fi	Clinical Groups	Pulmonology Group
Riitta Lahesmaa	Hospital District of Southwest Finland, Turku, Finland	rilahes@utu.fi	Clinical Groups	Pulmonology Group
Nizar Smaoui	Abbvie, Chicago, IL, United States	nizar.smaoui@abbvie.com	Clinical Groups	Pulmonology Group

For peer review only - http://bmjopen.bmj.com/site/about/guidelines.xhtml

Glenda Lassi	Astra Zeneca, Cambridge, United Kingdom	glenda.lassi@astrazeneca.com	Clinical Groups	Pulmonology Group
Susan Eaton	Biogen, Cambridge, MA, United States	susan.eaton@biogen.com	Clinical Groups	Pulmonology Group
Hubert Chen	Genentech, San Francisco, CA, United States	chenh37@gene.com	Clinical Groups	Pulmonology Group
Rion Pendergrass	Genentech, San Francisco, CA, United States	penders2@gene.com	Clinical Groups	Pulmonology Group
Natalie Bowers	Genentech, San Francisco, CA, United States	bowersn1@gene.com	Clinical Groups	Pulmonology Group
Joanna Betts	GlaxoSmithKline, Brentford, United Kingdom	joanna.c.betts@gsk.com	Clinical Groups	Pulmonology Group
Kirsi Auro	GlaxoSmithKline, Espoo, Finland	kirsi.m.auro@gsk.com	Clinical Groups	Pulmonology Group
Rajashree Mishra	GlaxoSmithKline, Brentford, United Kingdom	rajashree.x.mishra@gsk.com	Clinical Groups	Pulmonology Group
Majd Mouded	Novartis, Basel, Switzerland	majd.mouded@novartis.com	Clinical Groups	Pulmonology Group
Debby Ngo	Novartis, Basel, Switzerland	debby.ngo@novartis.com	Clinical Groups	Pulmonology Group

For peer review only

For peer review only - http://bmjopen.bmj.com/site/about/guidelines.xhtml

Teemu Niiranen	Finnish Institute for Health and Welfare (THL), Helsinki, Finland	teemu.niiranen@thl.fi	Clinical Groups	Cardiometabolic Diseases Group
Felix Vaura	Finnish Institute for Health and Welfare (THL), Helsinki, Finland	fechva@utu.fi	Clinical Groups	Cardiometabolic Diseases Group
Veikko Salomaa	Finnish Institute for Health and Welfare (THL), Helsinki, Finland	veikko.salomaa@thl.fi	Clinical Groups	Cardiometabolic Diseases Group
Kaj Metsärinne	Hospital District of Southwest Finland, Turku, Finland	kaj.metsarinne@tyks.fi	Clinical Groups	Cardiometabolic Diseases Group
Jenni Aittokallio	Hospital District of Southwest Finland, Turku, Finland	jemato@utu.fi	Clinical Groups	Cardiometabolic Diseases Group
Mika Kähönen	Pirkanmaa Hospital District, Tampere, Finland	mika.kahonen@uta.fi	Clinical Groups	Cardiometabolic Diseases Group
Jussi Hernesniemi	Pirkanmaa Hospital District, Tampere, Finland	jussi.hernesniemi@tuni.fi	Clinical Groups	Cardiometabolic Diseases Group

For peer review only - http://bmjopen.bmj.com/site/about/guidelines.xhtml

Daniel Gordin	Hospital District of Helsinki and Uusimaa, Helsinki, Finland	daniel.gordin@hus.fi	Clinical Groups	Cardiometabolic Diseases Group
Juha Sinisalo	Hospital District of Helsinki and Uusimaa, Helsinki, Finland	juha.sinisalo@hus.fi	Clinical Groups	Cardiometabolic Diseases Group
Marja-Riitta Taskinen	Hospital District of Helsinki and Uusimaa, Helsinki, Finland	marja-riitta.taskinen@helsinki.fi	Clinical Groups	Cardiometabolic Diseases Group
Tiinamaija Tuomi	Hospital District of Helsinki and Uusimaa, Helsinki, Finland	tiinamaija.tuomi@hus.fi	Clinical Groups	Cardiometabolic Diseases Group
Timo Hiltunen	Hospital District of Helsinki and Uusimaa, Helsinki, Finland	timo.hiltunen@hus.fi	Clinical Groups	Cardiometabolic Diseases Group
Jari Laukkanen	Central Finland Health Care District, Jyväskylä, Finland	jari.laukkanen@ksshp.fi	Clinical Groups	Cardiometabolic Diseases Group
Amanda Elliott	Institute for Molecular Medicine Finland (FIMM), HiLIFE, University of Helsinki, Helsinki, Finland; Broad Institute, Cambridge, MA, USA and	aelliott@broadinstitute.org	Clinical Groups	Cardiometabolic Diseases Group

For peer review only - http://bmjopen.bmj.com/site/about/guidelines.xhtml

	Massachusetts General Hospital, Boston, MA, USA			
Mary Pat Reeve	Institute for Molecular Medicine Finland (FIMM), HiLIFE, University of Helsinki, Helsinki, Finland	mary.reeve@helsinki.fi	Clinical Groups	Cardiometabolic Diseases Group
Sanni Ruotsalainen	Institute for Molecular Medicine Finland (FIMM), HiLIFE, University of Helsinki, Helsinki, Finland	sanni.ruotsalainen@helsinki.fi	Clinical Groups	Cardiometabolic Diseases Group
Benjamin Challis	Astra Zeneca, Cambridge, United Kingdom	benjamin.challis@astrazeneca.com	Clinical Groups	Cardiometabolic Diseases Group
Dirk Paul	Astra Zeneca, Cambridge, United Kingdom	dirk.paul@astrazeneca.com	Clinical Groups	Cardiometabolic Diseases Group
Julie Hunkapiller	Genentech, San Francisco, CA, United States	hunkapiller.julie@gene.com	Clinical Groups	Cardiometabolic Diseases Group
Natalie Bowers	Genentech, San Francisco, CA, United States	bowersn1@gene.com	Clinical Groups	Cardiometabolic Diseases Group

55

For peer review only

Rion Pendergrass	Genentech, San Francisco, CA, United States	penders2@gene.com	Clinical Groups	Cardiometabolic Diseases Group
Audrey Chu	GlaxoSmithKline, Brentford, United Kingdom	audrey.y.chu@gsk.com	Clinical Groups	Cardiometabolic Diseases Group
Kirsi Auro	GlaxoSmithKline, Espoo, Finland	kirsi.m.auro@gsk.com	Clinical Groups	Cardiometabolic Diseases Group
Dermot Reilly	Janssen Research & Development, LLC, Boston, MA, United States	dreill11@its.jnj.com	Clinical Groups	Cardiometabolic Diseases Group
Mike Mendelson	Novartis, Boston, MA, United States	mike.mendelson@novartis.com	Clinical Groups	Cardiometabolic Diseases Group
Jaakko Parkkinen	Pfizer, New York, NY, United States	jaakko.parkkinen@pfizer.com	Clinical Groups	Cardiometabolic Diseases Group
Melissa Miller	Pfizer, New York, NY, United States	melissa.r.miller@pfizer.com	Clinical Groups	Cardiometabolic Diseases Group
Tuomo Meretoja	Hospital District of Helsinki and Uusimaa, Helsinki, Finland	tuomo.meretoja@hus.fi	Clinical Groups	Oncology Group

For peer review only

Heikki Joensuu	Hospital District of Helsinki and Uusimaa, Helsinki, Finland	heikki.joensuu@hus.fi	Clinical Groups	Oncology Group
Olli Carpén	Hospital District of Helsinki and Uusimaa, Helsinki, Finland	olli.carpen@helsinki.fi	Clinical Groups	Oncology Group
Johanna Mattson	Hospital District of Helsinki and Uusimaa, Helsinki, Finland	johanna.mattson@hus.fi	Clinical Groups	Oncology Group
Eveliina Salminen	Hospital District of Helsinki and Uusimaa, Helsinki, Finland	eveliina.e.salminen@hus.fi	Clinical Groups	Oncology Group
Annika Auranen	Pirkanmaa Hospital District , Tampere, Finland	annika.auranen@pirha.fi	Clinical Groups	Oncology Group
Peeter Karihtala	Helsinki University Hospital Comprehensive Cancer Centre, Helsinki, Finland	peeter.karihtala@oulu.fi	Clinical Groups	Oncology Group
Päivi Auvinen	Northern Savo Hospital District, Kuopio, Finland	paivi.auvinen@kuh.fi	Clinical Groups	Oncology Group
Klaus Elenius	Hospital District of Southwest Finland, Turku, Finland	klaus.elenius@utu.fi	Clinical Groups	Oncology Group
Johanna Schleutker	Hospital District of Southwest Finland, Turku, Finland	johanna.schleutker@utu.fi	Clinical Groups	Oncology Group
Esa Pitkänen	Institute for Molecular Medicine Finland (FIMM), HiLIFE,	esa.pitkanen@helsinki.fi	Clinical Groups	Oncology Group

For peer review only - http://bmjopen.bmj.com/site/about/guidelines.xhtml

	University of Helsinki, Helsinki, Finland			
Nina Mars	Institute for Molecular Medicine Finland (FIMM), HiLIFE, University of Helsinki, Helsinki, Finland	nina.mars@helsinki.fi	Clinical Groups	Oncology Group
Mark Daly	Institute for Molecular Medicine Finland (FIMM), HiLIFE, University of Helsinki, Helsinki, Finland; Broad Institute of MIT and Harvard; Massachusetts General Hospital	mark.daly@helsinki.fi	Clinical Groups	Oncology Group
Relja Popovic	Abbvie, Chicago, IL, United States	relja.popovic@abbvie.com	Clinical Groups	Oncology Group
Jeffrey Waring	Abbvie, Chicago, IL, United States	jeff.waring@abbvie.com	Clinical Groups	Oncology Group
Bridget Riley-Gillis	Abbvie, Chicago, IL, United States	bridget.rileygillis@abbvie.com	Clinical Groups	Oncology Group
Anne Lehtonen	Abbvie, Chicago, IL, United States	anne.lehtonen@abbvie.com	Clinical Groups	Oncology Group
Jennifer Schutzman	Genentech, San Francisco, CA, United States	schutzman.jennifer@gene.com	Clinical Groups	Oncology Group

For peer review only - http://bmjopen.bmj.com/site/about/guidelines.xhtml

Julie Hunkapiller	Genentech, San Francisco, CA, United States	hunkapiller.julie@gene.com	Clinical Groups	Oncology Group
Natalie Bowers	Genentech, San Francisco, CA, United States	bowersn1@gene.com	Clinical Groups	Oncology Group
Rion Pendergrass	Genentech, San Francisco, CA, United States	penders2@gene.com	Clinical Groups	Oncology Group
Diptee Kulkarni	GlaxoSmithKline, Brentford, United Kingdom	diptee.a.kulkarni@gsk.com	Clinical Groups	Oncology Group
Kirsi Auro	GlaxoSmithKline, Espoo, Finland	kirsi.m.auro@gsk.com	Clinical Groups	Oncology Group
Alessandro Porello	Janssen Research & Development, LLC, Spring House, PA, United States	APorrell@ITS.JNJ.com	Clinical Groups	Oncology Group
Andrey Loboda	Merck, Kenilworth, NJ, United States	andrey_loboda@merck.com	Clinical Groups	Oncology Group
Heli Lehtonen	Pfizer, New York, NY, United States	heli.lehtonen@pfizer.com	Clinical Groups	Oncology Group
Stefan McDonough	Pfizer, New York, NY, United States	stefan.McDonough@pfizer.com	Clinical Groups	Oncology Group
Sauli Vuoti	Janssen-Cilag Oy, Espoo, Finland	svuoti@its.jnj.com	Clinical Groups	Oncology Group

For peer review only - http://bmjopen.bmj.com/site/about/guidelines.xhtml

Kai Kaarniranta	Northern Savo Hospital District, Kuopio, Finland	kai.kaarniranta@uef.fi	Clinical Groups	Opthalmology Group
Joni A Turunen	Helsinki University Hospital and University of Helsinki, Helsinki, Finland; Eye Genetics Group, Folkhälsan Research Center, Helsinki, Finland	joni.turunen@helsinki.fi	Clinical Groups	Opthalmology Group
Terhi Ollila	Hospital District of Helsinki and Uusimaa, Helsinki, Finland	terhi.ollila@hus.fi	Clinical Groups	Opthalmology Group
Hannu Uusitalo	Pirkanmaa Hospital District, Tampere, Finland	hannu.uusitalo@tuni.fi	Clinical Groups	Opthalmology Group
Juha Karjalainen	Institute for Molecular Medicine Finland (FIMM), HiLIFE, University of Helsinki, Helsinki, Finland	juha.karjalainen@helsinki.fi	Clinical Groups	Opthalmology Group
Esa Pitkänen	Institute for Molecular Medicine Finland (FIMM), HiLIFE, University of Helsinki, Helsinki, Finland	esa.pitkanen@helsinki.fi	Clinical Groups	Opthalmology Group
Mengzhen Liu	Abbvie, Chicago, IL, United States	mengzhen.liu@abbvie.com	Clinical Groups	Opthalmology Group
Heiko Runz	Biogen, Cambridge, MA, United States	heiko.runz@biogen.com	Clinical Groups	Opthalmology Group

For peer review only

Stephanie Loomis	Biogen, Cambridge, MA, United States	stephanie.loomis@biogen.com	Clinical Groups	Opthalmology Group
Erich Strauss	Genentech, San Francisco, CA, United States	strauss.erich@gene.com	Clinical Groups	Opthalmology Group
Natalie Bowers	Genentech, San Francisco, CA, United States	bowersn1@gene.com	Clinical Groups	Opthalmology Group
Hao Chen	Genentech, San Francisco, CA, United States	haoc@gene.com	Clinical Groups	Opthalmology Group
Rion Pendergrass	Genentech, San Francisco, CA, United States	penders2@gene.com	Clinical Groups	Opthalmology Group
Kaisa Tasanen	Wellbeing Services County of North Ostrobothnia, University of Oulu, Oulu, Finland	kaisa.tasanen-maatta@oulu.fi	Clinical Groups	Dermatology Group
Laura Huilaja	Wellbeing Services County of North Ostrobothnia, University of Oulu, Oulu, Finland	laura.huilaja@oulu.fi	Clinical Groups	Dermatology Group
Katariina Hannula-Jouppi	Hospital District of Helsinki and Uusimaa, Helsinki, Finland	katariina.hannula-jouppi@hus.fi	Clinical Groups	Dermatology Group
Teea Salmi	Pirkanmaa Hospital District, Tampere, Finland	teea.salmi@pshp.fi	Clinical Groups	Dermatology Group

For peer review only

For peer review only - http://bmjopen.bmj.com/site/about/guidelines.xhtml

Sirkku Peltonen	Hospital District of Southwest Finland, Turku, Finland	sipelto@utu.fi	Clinical Groups	Dermatology Group
Leena Koulu	Hospital District of Southwest Finland, Turku, Finland	leena.koulu@tyks.fi	Clinical Groups	Dermatology Group
Nizar Smaoui	Abbvie, Chicago, IL, United States	nizar.smaoui@abbvie.com	Clinical Groups	Dermatology Group
Fedik Rahimov	Abbvie, Chicago, IL, United States	fedik.rahimov@abbvie.com	Clinical Groups	Dermatology Group
Anne Lehtonen	Abbvie, Chicago, IL, United States	anne.lehtonen@abbvie.com	Clinical Groups	Dermatology Group
David Choy	Genentech, San Francisco, CA, United States	choy.david@gene.com	Clinical Groups	Dermatology Group
Rion Pendergrass	Genentech, San Francisco, CA, United States	penders2@gene.com	Clinical Groups	Dermatology Group
Dawn Waterworth	Janssen Research & Development, LLC, Spring House, PA, United States	dwaterwo@its.jnj.com	Clinical Groups	Dermatology Group
Kirsi Kalpala	Pfizer, New York, NY, United States	kirsi.kalpala@pfizer.com	Clinical Groups	Dermatology Group
Ying Wu	Pfizer, New York, NY, United States	ying.wu3@pfizer.com	Clinical Groups	Dermatology Group

For peer review only - http://bmjopen.bmj.com/site/about/guidelines.xhtml

Pirkko Pussinen	Hospital District of Helsinki and Uusimaa, Helsinki, Finland	pirkko.pussinen@helsinki.fi	Clinical Groups	Odontology Group
Aino Salminen	Hospital District of Helsinki and Uusimaa, Helsinki, Finland	aino.m.salminen@helsinki.fi	Clinical Groups	Odontology Group
Tuula Salo	Hospital District of Helsinki and Uusimaa, Helsinki, Finland	tuula.salo@helsinki.fi	Clinical Groups	Odontology Group
David Rice	Hospital District of Helsinki and Uusimaa, Helsinki, Finland	david.rice@helsinki.fi	Clinical Groups	Odontology Group
Pekka Nieminen	Hospital District of Helsinki and Uusimaa, Helsinki, Finland	pekka.nieminen@helsinki.fi	Clinical Groups	Odontology Group
Ulla Palotie	Hospital District of Helsinki and Uusimaa, Helsinki, Finland	ulla.palotie@helsinki.fi	Clinical Groups	Odontology Group
Maria Siponen	Northern Savo Hospital District, Kuopio, Finland	maria.siponen@uef.fi	Clinical Groups	Odontology Group
Liisa Suominen	Northern Savo Hospital District, Kuopio, Finland	liisa.suominen@uef.fi	Clinical Groups	Odontology Group
Päivi Mäntylä	Northern Savo Hospital District, Kuopio, Finland	paivi.mantyla@uef.fi	Clinical Groups	Odontology Group
Ulvi Gursoy	Hospital District of Southwest Finland, Turku, Finland	ulvi.gursoy@utu.fi	Clinical Groups	Odontology Group

For peer review only - http://bmjopen.bmj.com/site/about/guidelines.xhtml

Vuokko Anttonen	Wellbeing Services County of North Ostrobothnia, University of Oulu, Oulu, Finland	vuokko.anttonen@oulu.fi	Clinical Groups	Odontology Group
Kirsi Sipilä	Research Unit of Oral Health Sciences Faculty of Medicine, University of Oulu, Oulu, Finland; Medical Research Center, Oulu, Oulu University Hospital and University of Oulu, Oulu, Finland	kirsi.sipila@oulu.fi	Clinical Groups	Odontology Group
Rion Pendergrass	Genentech, San Francisco, CA, United States	pendergrass.sarah@gene.com	Clinical Groups	Odontology Group
Hannele Laivuori	Institute for Molecular Medicine Finland (FIMM), HiLIFE, University of Helsinki, Helsinki, Finland	hannele.laivuori@helsinki.fi	Clinical Groups	Women's Health and Reproduction Group
Venla Kurra	Pirkanmaa Hospital District, Tampere, Finland	venla.kurra@tuni.fi	Clinical Groups	Women's Health and Reproduction Group
Laura Kotaniemi-Talonen	Pirkanmaa Hospital District, Tampere, Finland	laura.kotaniemi-talonen@tuni.fi	Clinical Groups	Women's Health and Reproduction Group

For peer review only

Oskari Heikinheimo	Hospital District of Helsinki and Uusimaa, Helsinki, Finland	oskari.heikinheimo@helsinki.fi	Clinical Groups	Women's Health and Reproduction Group
Ilkka Kalliala	Hospital District of Helsinki and Uusimaa, Helsinki, Finland	ilkka.kalliala@hus.fi	Clinical Groups	Women's Health and Reproduction Group
Lauri Aaltonen	Hospital District of Helsinki and Uusimaa, Helsinki, Finland	lauri.aaltonen@helsinki.fi	Clinical Groups	Women's Health and Reproduction Group
Varpu Jokimaa	Hospital District of Southwest Finland, Turku, Finland	varpu.jokimaa@utu.fi	Clinical Groups	Women's Health and Reproduction Group
Johannes Kettunen	Wellbeing Services County of North Ostrobothnia, Oulu, Finland	Johannes.Kettunen@oulu.fi	Clinical Groups	Women's Health and Reproduction Group
Marja Vääräsmäki	Wellbeing Services County of North Ostrobothnia, Oulu, Finland	marja.vaarasmaki@oulu.fi	Clinical Groups	Women's Health and

For peer review only - http://bmjopen.bmj.com/site/about/guidelines.xhtml

				Reproduction Group
Outi Uimari	University of Oulu, Wellbeing Services County of North Ostrobothnia, Oulu, Finland	outi.uimari@oulu.fi	Clinical Groups	Women's Health and Reproduction Group
Laure Morin-Papunen	Wellbeing Services County of North Ostrobothnia, Oulu, Finland	lmp@cc.oulu.fi	Clinical Groups	Women's Health and Reproduction Group
Maarit Niinimäki	Wellbeing Services County of North Ostrobothnia, Oulu, Finland	maarit.niinimaki@oulu.fi	Clinical Groups	Women's Health and Reproduction Group
Terhi Piltonen	University of Oulu, Wellbeing Services County of North Ostrobothnia, Oulu, Finland	terhi.piltonen@oulu.fi	Clinical Groups	Women's Health and Reproduction Group
Katja Kivinen	Institute for Molecular Medicine Finland (FIMM), HiLIFE, University of Helsinki, Helsinki, Finland	katja.kivinen@helsinki.fi	Clinical Groups	Women's Health and Reproduction Group

For peer review only - http://bmjopen.bmj.com/site/about/guidelines.xhtml

Elisabeth Widen	Institute for Molecular Medicine Finland (FIMM), HiLIFE, University of Helsinki, Helsinki, Finland	elisabeth.widen@helsinki.fi	Clinical Groups	Women's Health and Reproduction Group
Taru Tukiainen	Institute for Molecular Medicine Finland (FIMM), HiLIFE, University of Helsinki, Helsinki, Finland	taru.tukiainen@helsinki.fi	Clinical Groups	Women's Health and Reproduction Group
Mary Pat Reeve	Institute for Molecular Medicine Finland (FIMM), HiLIFE, University of Helsinki, Helsinki, Finland	mary.reeve@helsinki.fi	Clinical Groups	Women's Health and Reproduction Group
Mark Daly	Institute for Molecular Medicine Finland (FIMM), HiLIFE, University of Helsinki, Helsinki, Finland; Broad Institute of MIT and Harvard; Massachusetts General Hospital	mark.daly@helsinki.fi	Clinical Groups	Women's Health and Reproduction Group
Niko Välimäki	University of Helsinki, Helsinki, Finland	niko.valimaki@helsinki.fi	Clinical Groups	Women's Health and Reproduction Group

For peer review only - http://bmjopen.bmj.com/site/about/guidelines.xhtml

Eija Laakkonen	University of Jyväskylä, Jyväskylä, Finland	eija.k.laakkonen@jyu.fi	Clinical Groups	Women's Health and Reproduction Group
Jaakko Tyrmi	University of Oulu, Oulu, Finland / University of Tampere, Tampere, Finland	jaakko.tyrmi@oulu.fi	Clinical Groups	Women's Health and Reproduction Group
Heidi Silven	University of Oulu, Oulu, Finland	heidi.silven@student.oulu.fi	Clinical Groups	Women's Health and Reproduction Group
Eeva Sliz	University of Oulu, Oulu, Finland	eeva.sliz@oulu.fi	Clinical Groups	Women's Health and Reproduction Group
Riikka Arffman	University of Oulu, Oulu, Finland	riikka.arffman@oulu.fi	Clinical Groups	Women's Health and Reproduction Group
Susanna Savukoski	University of Oulu, Oulu, Finland	susanna.savukoski@oulu.fi	Clinical Groups	Women's Health and

For peer review only

				Reproduction Group
Triin Laisk	Estonian biobank, Tartu, Estonia	triin.laisk@ut.ee	Clinical Groups	Women's Health and Reproduction Group
Natalia Pujol	Estonian biobank, Tartu, Estonia	natalia.pujolgualdo@oulu.fi	Clinical Groups	Women's Health and Reproduction Group
Mengzhen Liu	Abbvie, Chicago, IL, United States	mengzhen.liu@abbvie.com	Clinical Groups	Women's Health and Reproduction Group
Bridget Riley-Gillis	Abbvie, Chicago, IL, United States	bridget.rileygillis@abbvie.com	Clinical Groups	Women's Health and Reproduction Group
Rion Pendergrass	Genentech, San Francisco, CA, United States	penders2@gene.com	Clinical Groups	Women's Health and Reproduction Group

For peer review only

Janet Kumar	GlaxoSmithKline, Collegeville, PA, United States	janet.x.kumar@gsk.com	Clinical Groups	Women's Health and Reproduction Group
Kirsi Auro	GlaxoSmithKline, Espoo, Finland	kirsi.m.auro@gsk.com	Clinical Groups	Women's Health and Reproduction Group
Iiris Hovatta	University of Helsinki, Finland	iiris.hovatta@helsinki.fi	Clinical Groups	Depression group
Chia-Yen Chen	Biogen, Cambridge, MA, United States	chiayen.chen@biogen.com	Clinical Groups	Depression group
Erkki Isometsä	Hospital District of Helsinki and Uusimaa, Helsinki, Finland	erkki.isometsa@hus.fi	Clinical Groups	Depression group
Hanna Ollila	Institute for Molecular Medicine Finland (FIMM), HiLIFE, University of Helsinki, Helsinki, Finland	hanna.m.ollila@helsinki.fi	Clinical Groups	Depression group
Jaana Suvisaari	Finnish Institute for Health and Welfare (THL), Helsinki, Finland	jaana.suvisaari@thl.fi	Clinical Groups	Depression group
Thomas Damm Als	Aarhus University, Denmark	tda@biomed.au.dk	Clinical Groups	Depression group

For peer review only - http://bmjopen.bmj.com/site/about/guidelines.xhtml

Antti Mäkitie	Department of Otorhinolaryngology - Head and Neck Surgery, University of Helsinki and Helsinki University Hospital, Helsinki, Finland	antti.makitie@helsinki.fi	Clinical Groups	ENT (ear, nose and throat) Group
Argyro Bizaki-Vallaskangas	Pirkanmaa Hospital District, Tampere, Finland	argyro.bizaki-vallaskangas@tuni.fi	Clinical Groups	ENT (ear, nose and throat) Group
Sanna Toppila-Salmi	University of Helsinki, Finland	sanna.salmi@helsinki.fi	Clinical Groups	ENT (ear, nose and throat) Group
Tytti Willberg	Hospital District of Southwest Finland, Turku, Finland	tytti.willberg@tyks.fi	Clinical Groups	ENT (ear, nose and throat) Group
Elmo Saarentaus	Institute for Molecular Medicine Finland (FIMM), HiLIFE, University of Helsinki, Helsinki, Finland	elmo.saarentaus@helsinki.fi	Clinical Groups	ENT (ear, nose and throat) Group
Antti Aarnisalo	Hospital District of Helsinki and Uusimaa, Helsinki, Finland	antti.aarnisalo@hus.fi	Clinical Groups	ENT (ear, nose and throat) Group

For peer review only

For peer review only - http://bmjopen.bmj.com/site/about/guidelines.xhtml

Eveliina Salminen	Hospital District of Helsinki and Uusimaa, Helsinki, Finland	eveliina.e.salminen@hus.fi	Clinical Groups	ENT (ear, nose and throat) Group
Elisa Rahikkala	Wellbeing Services County of North Ostrobothnia, Oulu, Finland	elisa.rahikkala@ppshp.fi	Clinical Groups	ENT (ear, nose and throat) Group
Johannes Kettunen	University of Oulu, Wellbeing Services County of North Ostrobothnia, Oulu, Finland	johannes.kettunen@oulu.fi	Clinical Groups	ENT (ear, nose and throat) Group
Kristiina Aittomäki	Department of Medical Genetics, Helsinki University Central Hospital, Helsinki, Finland	kristiina.aittomaki@helsinki.fi	Clinical Groups	POI (premature ovarian failure) Group
Fredrik Åberg	Transplantation and Liver Surgery Clinic, Helsinki University Hospital, Helsinki University, Helsinki, Finland	fredrik.aberg@helsinki.fi	Clinical Groups	LiverScore Group
Mitja Kurki	Institute for Molecular Medicine Finland (FIMM), HiLIFE, University of Helsinki, Helsinki, Finland; Broad Institute, Cambridge, MA, United States	mkurki@broadinstitute.org	FinnGen Analysis working group	FinnGen Analysis working group

For peer review only

Samuli Ripatti	Institute for Molecular Medicine Finland (FIMM), HiLIFE, University of Helsinki, Helsinki, Finland	samuli.ripatti@helsinki.fi	FinnGen Analysis working group	FinnGen Analysis working group
Mark Daly	Institute for Molecular Medicine, Finland (FIMM), HiLIFE, University of Helsinki, Helsinki, Finland; Broad Institute of MIT and Harvard; Massachusetts General Hospital	mark.daly@helsinki.fi	FinnGen Analysis working group	FinnGen Analysis working group
Juha Karjalainen	Institute for Molecular Medicine Finland (FIMM), HiLIFE, University of Helsinki, Helsinki, Finland	juha.karjalainen@helsinki.fi	FinnGen Analysis working group	FinnGen Analysis working group
Aki Havulinna	Institute for Molecular Medicine Finland (FIMM), HiLIFE, University of Helsinki, Helsinki, Finland; Finnish Institute for Health and Welfare (THL), Helsinki, Finland	aki.havulinna@helsinki.fi	FinnGen Analysis working group	FinnGen Analysis working group
Juha Mehtonen	Institute for Molecular Medicine Finland (FIMM), HiLIFE,	juha.mehtonen@helsinki.fi	FinnGen Analysis working group	FinnGen Analysis working group

For peer review only - http://bmjopen.bmj.com/site/about/guidelines.xhtml

	University of Helsinki, Helsinki, Finland			
Priit Palta	Institute for Molecular Medicine Finland (FIMM), HiLIFE, University of Helsinki, Helsinki, Finland	priit.palta@helsinki.fi	FinnGen Analysis working group	FinnGen Analysis working group
Shabbeer Hassan	Institute for Molecular Medicine Finland (FIMM), HiLIFE, University of Helsinki, Helsinki, Finland	shabbeer.hassan@helsinki.fi	FinnGen Analysis working group	FinnGen Analysis working group
Pietro Della Briotta Parolo	Institute for Molecular Medicine Finland (FIMM), HiLIFE, University of Helsinki, Helsinki, Finland	pietro.dellabriottaparolo@helsinki.fi	FinnGen Analysis working group	FinnGen Analysis working group
Wei Zhou	Broad Institute, Cambridge, MA, United States	wzhou@broadinstitute.org	FinnGen Analysis working group	FinnGen Analysis working group
Mutaamba Maasha	Broad Institute, Cambridge, MA, United States	mmaasha@broadinstitute.org	FinnGen Analysis working group	FinnGen Analysis working group
Shabbeer Hassan	Institute for Molecular Medicine Finland (FIMM), HiLIFE,	shabbeer.hassan@helsinki.fi	FinnGen Analysis working group	FinnGen Analysis working group

For peer review only

	University of Helsinki, Helsinki, Finland			
Susanna Lemmelä	Institute for Molecular Medicine Finland (FIMM), HiLIFE, University of Helsinki, Helsinki, Finland	susanna.lemmela@helsinki.fi	FinnGen Analysis working group	FinnGen Analysis working group
Manuel Rivas	University of Stanford, Stanford, CA, United States	mrivas@stanford.edu	FinnGen Analysis working group	FinnGen Analysis working group
Mari E. Niemi	Institute for Molecular Medicine Finland (FIMM), HiLIFE, University of Helsinki, Helsinki, Finland	mari.e.niemi@helsinki.fi	FinnGen Analysis working group	FinnGen Analysis working group
Aarno Palotie	Institute for Molecular Medicine Finland (FIMM), HiLIFE, University of Helsinki, Helsinki, Finland	aarno.palotie@helsinki.fi	FinnGen Analysis working group	FinnGen Analysis working group
Aoxing Liu	Institute for Molecular Medicine Finland (FIMM), HiLIFE, University of Helsinki, Helsinki, Finland	aoxing.liu@helsinki.fi	FinnGen Analysis working group	FinnGen Analysis working group

For peer review only - http://bmjopen.bmj.com/site/about/guidelines.xhtml

Arto Lehisto	Institute for Molecular Medicine Finland (FIMM), HiLIFE, University of Helsinki, Helsinki, Finland	arto.lehisto@helsinki.fi	FinnGen Analysis working group	FinnGen Analysis working group
Andrea Ganna	Institute for Molecular Medicine Finland (FIMM), HiLIFE, University of Helsinki, Helsinki, Finland	aganna@broadinstitute.org	FinnGen Analysis working group	FinnGen Analysis working group
Vincent Llorens	Institute for Molecular Medicine Finland (FIMM), HiLIFE, University of Helsinki, Helsinki, Finland	vincent.llorens@helsinki.fi	FinnGen Analysis working group	FinnGen Analysis working group
Hannele Laivuori	Institute for Molecular Medicine Finland (FIMM), HiLIFE, University of Helsinki, Helsinki, Finland	hannele.laivuori@helsinki.fi	FinnGen Analysis working group	FinnGen Analysis working group
Taru Tukiainen	Institute for Molecular Medicine Finland (FIMM), HiLIFE, University of Helsinki, Helsinki, Finland	taru.tukiainen@helsinki.fi	FinnGen Analysis working group	FinnGen Analysis working group
Mary Pat Reeve	Institute for Molecular Medicine Finland (FIMM), HiLIFE,	mary.reeve@helsinki.fi	FinnGen Analysis working group	FinnGen Analysis working group

For peer review only - http://bmjopen.bmj.com/site/about/guidelines.xhtml

	University of Helsinki, Helsinki, Finland			
Henrike Heyne	Institute for Molecular Medicine Finland (FIMM), HiLIFE, University of Helsinki, Helsinki, Finland	hheyne@broadinstitute.org	FinnGen Analysis working group	FinnGen Analysis working group
Nina Mars	Institute for Molecular Medicine Finland (FIMM), HiLIFE, University of Helsinki, Helsinki, Finland	nina.mars@helsinki.fi	FinnGen Analysis working group	FinnGen Analysis working group
Joel Rämö	Institute for Molecular Medicine Finland (FIMM), HiLIFE, University of Helsinki, Helsinki, Finland	joel.ramo@helsinki.fi	FinnGen Analysis working group	FinnGen Analysis working group
Elmo Saarentaus	Institute for Molecular Medicine Finland (FIMM), HiLIFE, University of Helsinki, Helsinki, Finland	elmo.saarentaus@helsinki.fi	FinnGen Analysis working group	FinnGen Analysis working group
Hanna Ollila	Institute for Molecular Medicine Finland (FIMM), HiLIFE, University of Helsinki, Helsinki, Finland	hanna.m.ollila@helsinki.fi	FinnGen Analysis working group	FinnGen Analysis working group

For peer review only - http://bmjopen.bmj.com/site/about/guidelines.xhtml

Rodos Rodosthenous	Institute for Molecular Medicine Finland (FIMM), HiLIFE, University of Helsinki, Helsinki, Finland	rodos.rodosthenous@helsinki.fi	FinnGen Analysis working group	FinnGen Analysis working group
Satu Strausz	Institute for Molecular Medicine Finland (FIMM), HiLIFE, University of Helsinki, Helsinki, Finland	satu.strausz@helsinki.fi	FinnGen Analysis working group	FinnGen Analysis working group
Tuula Palotie	University of Helsinki and Hospital District of Helsinki and Uusimaa, Helsinki, Finland	tuula.palotie@helsinki.fi	FinnGen Analysis working group	FinnGen Analysis working group
Kimmo Palin	University of Helsinki, Helsinki, Finland	kimmo.palin@helsinki.fi	FinnGen Analysis working group	FinnGen Analysis working group
Javier Garcia-Tabuenca	University of Tampere, Tampere, Finland	javier.graciatabuenca@tuni.fi	FinnGen Analysis working group	FinnGen Analysis working group
Harri Siirtola	University of Tampere, Tampere, Finland	harri.siirtola@tuni.fi	FinnGen Analysis working group	FinnGen Analysis working group
Tuomo Kiiskinen	Institute for Molecular Medicine Finland (FIMM), HiLIFE,	tuomo.kiiskinen@helsinki.fi	FinnGen Analysis working group	FinnGen Analysis working group

For peer review only - http://bmjopen.bmj.com/site/about/guidelines.xhtml

	University of Helsinki, Helsinki, Finland			
Jiwoo Lee	Institute for Molecular Medicine Finland (FIMM), HiLIFE, University of Helsinki, Helsinki, Finland; Broad Institute, Cambridge, MA, United States	jiwoo.lee@helsinki.fi	FinnGen Analysis working group	FinnGen Analysis working group
Kristin Tsuo	Institute for Molecular Medicine Finland (FIMM), HiLIFE, University of Helsinki, Helsinki, Finland; Broad Institute, Cambridge, MA, United States	kristintsuo@fas.harvard.edu	FinnGen Analysis working group	FinnGen Analysis working group
Amanda Elliott	Institute for Molecular Medicine Finland (FIMM), HiLIFE, University of Helsinki, Helsinki, Finland; Broad Institute, Cambridge, MA, USA and Massachusetts General Hospital, Boston, MA, USA	aelliott@broadinstitute.org	FinnGen Analysis working group	FinnGen Analysis working group
Kati Kristiansson	THL Biobank / Finnish Institute for Health and Welfare (THL), Helsinki, Finland	kati.kristiansson@thl.fi	FinnGen Analysis working group	FinnGen Analysis working group

For peer review only - http://bmjopen.bmj.com/site/about/guidelines.xhtml

Mikko Arvas	Finnish Red Cross Blood Service / Finnish Hematology Registry and Clinical Biobank, Helsinki, Finland	mikko.arvas@veripalvelu.fi	FinnGen Analysis working group	FinnGen Analysis working group
Kati Hyvärinen	Finnish Red Cross Blood Service, Helsinki, Finland	kati.hyvarinen@veripalvelu.fi	FinnGen Analysis working group	FinnGen Analysis working group
Jarmo Ritari	Finnish Red Cross Blood Service, Helsinki, Finland	jarmo.ritari@veripalvelu.fi	FinnGen Analysis working group	FinnGen Analysis working group
Olli Carpén	Helsinki Biobank / Helsinki University and Hospital District of Helsinki and Uusimaa, Helsinki	olli.carpen@helsinki.fi	FinnGen Analysis working group	FinnGen Analysis working group
Johannes Kettunen	Northern Finland Biobank Borealis / University of Oulu / Wellbeing Services County of North Ostrobothnia, Oulu, Finland	johannes.kettunen@oulu.fi	FinnGen Analysis working group	FinnGen Analysis working group
Katri Pylkäs	University of Oulu, Oulu, Finland	katri.pylkas@oulu.fi	FinnGen Analysis working group	FinnGen Analysis working group
Eeva Sliz	University of Oulu, Oulu, Finland	eeva.sliz@oulu.fi	FinnGen Analysis working group	FinnGen Analysis working group

For peer review only - http://bmjopen.bmj.com/site/about/guidelines.xhtml

Minna Karjalainen	University of Oulu, Oulu, Finland	minna.k.karjalainen@oulu.fi	FinnGen Analysis working group	FinnGen Analysis working group
Tuomo Mantere	Northern Finland Biobank Borealis / University of Oulu / Wellbeing Services County of North Ostrobothnia, Oulu, Finland	tuomo.mantere@oulu.fi	FinnGen Analysis working group	FinnGen Analysis working group
Eeva Kangasniemi	Finnish Clinical Biobank Tampere / University of Tampere / The Wellbeing Services County of Pirkanmaa, Tampere, Finland	eeva.kangasniemi@pirha.fi	FinnGen Analysis working group	FinnGen Analysis working group
Sami Heikkinen	University of Eastern Finland, Kuopio, Finland	sami.heikkinen@uef.fi	FinnGen Analysis working group	FinnGen Analysis working group
Arto Mannermaa	Biobank of Eastern Finland / University of Eastern Finland / Northern Savo Hospital District, Kuopio, Finland	arto.mannermaa@uef.fi	FinnGen Analysis working group	FinnGen Analysis working group
Eija Laakkonen	University of Jyväskylä, Jyväskylä, Finland	eija.k.laakkonen@jyu.fi	FinnGen Analysis working group	FinnGen Analysis working group

For peer review only

Nina Pitkänen	Auria Biobank / University of Turku / Hospital District of Southwest Finland, Turku, Finland	Niina.Pitkanen@tyks.fi	FinnGen Analysis working group	FinnGen Analysis working group
Samuel Lessard	Translational Sciences, Sanofi R&D, Framingham, MA, USA	samuel.lessard@sanofi.com	FinnGen Analysis working group	FinnGen Analysis working group
Clément Chatelain	Translational Sciences, Sanofi R&D, Framingham, MA, USA	clement.chatelain@sanofi.com	FinnGen Analysis working group	FinnGen Analysis working group
Perttu Terho	Auria Biobank / University of Turku / Hospital District of Southwest Finland, Turku, Finland	perttu.terho@tyks.fi	Biobank directors	Biobank directors
Sirpa Soini	THL Biobank / Finnish Institute for Health and Welfare (THL), Helsinki, Finland	sirpa.soini@thl.fi	Biobank directors	Biobank directors
Jukka Partanen	Finnish Red Cross Blood Service / Finnish Hematology Registry and Clinical Biobank, Helsinki, Finland	jukka.partanen@veripalvelu.fi	Biobank directors	Biobank directors
Eero Punkka	Helsinki Biobank / Helsinki University and Hospital District of Helsinki and Uusimaa, Helsinki	eero.punkka@hus.fi	Biobank directors	Biobank directors
Raisa Serpi	Northern Finland Biobank Borealis / University of Oulu / Wellbeing	raisa.serpi@pohde.fi	Biobank directors	Biobank directors

For peer review only

For peer review only - http://bmjopen.bmj.com/site/about/guidelines.xhtml

	Services County of North Ostrobothnia, Oulu, Finland			
Sanna Siltanen	Finnish Clinical Biobank Tampere / University of Tampere / The Wellbeing Services County of Pirkanmaa, Tampere, Finland	sanna.siltanen@pirha.fi	Biobank directors	Biobank directors
Veli-Matti Kosma	Biobank of Eastern Finland / University of Eastern Finland / Northern Savo Hospital District, Kuopio, Finland	veli-matti.kosma@uef.fi	Biobank directors	Biobank directors
Teijo Kuopio	Central Finland Biobank / University of Jyväskylä / Central Finland Health Care District, Jyväskylä, Finland	teijo.kuopio@ksshp.fi	Biobank directors	Biobank directors
Anu Jalanko	Institute for Molecular Medicine Finland (FIMM), HiLIFE, University of Helsinki, Helsinki, Finland	anu.jalanko@helsinki.fi	FinnGen Teams	Administration
Huei-Yi Shen	Institute for Molecular Medicine Finland (FIMM), HiLIFE, University of Helsinki, Helsinki, Finland	huei-yi.shen@helsinki.fi	FinnGen Teams	Administration

For peer review only - http://bmjopen.bmj.com/site/about/guidelines.xhtml

Risto Kajanne	Institute for Molecular Medicine Finland (FIMM), HiLIFE, University of Helsinki, Helsinki, Finland	risto.kajanne@helsinki.fi	FinnGen Teams	Administration
Mervi Aavikko	Institute for Molecular Medicine Finland (FIMM), HiLIFE, University of Helsinki, Helsinki, Finland	mervi.aavikko@helsinki.fi	FinnGen Teams	Administration
Henna Palin	Finnish Clinical Biobank Tampere / University of Tampere / The Wellbeing Services County of Pirkanmaa, Tampere, Finland	henna.palin@pirha.fi	FinnGen Teams	Administration
Malla-Maria Linna	Helsinki Biobank / Helsinki University and Hospital District of Helsinki and Uusimaa, Helsinki	malla-maria.linna@hus.fi	FinnGen Teams	Administration
Mitja Kurki	Institute for Molecular Medicine Finland (FIMM), HiLIFE, University of Helsinki, Helsinki, Finland; Broad Institute, Cambridge, MA, United States	mkurki@broadinstitute.org	FinnGen Teams	Analysis
Juha Karjalainen	Institute for Molecular Medicine Finland (FIMM), HiLIFE,	juha.karjalainen@helsinki.fi	FinnGen Teams	Analysis

For peer review only - http://bmjopen.bmj.com/site/about/guidelines.xhtml

	University of Helsinki, Helsinki, Finland			
Pietro Della Briotta Parolo	Institute for Molecular Medicine Finland (FIMM), HiLIFE, University of Helsinki, Helsinki, Finland	pietro.dellabriottaparolo@helsinki.fi	FinnGen Teams	Analysis
Arto Lehisto	Institute for Molecular Medicine Finland (FIMM), HiLIFE, University of Helsinki, Helsinki, Finland	arto.lehisto@helsinki.fi	FinnGen Teams	Analysis
Juha Mehtonen	Institute for Molecular Medicine Finland (FIMM), HiLIFE, University of Helsinki, Helsinki, Finland	juha.mehtonen@helsinki.fi	FinnGen Teams	Analysis
Wei Zhou	Broad Institute, Cambridge, MA, United States	wzhou@broadinstitute.org	FinnGen Teams	Analysis
Masahiro Kanai	Broad Institute, Cambridge, MA, United States	mkanai@broadinstitute.org	FinnGen Teams	Analysis
Mutaamba Maasha	Broad Institute, Cambridge, MA, United States	mmaasha@broadinstitute.org	FinnGen Teams	Analysis
Hannele Laivuori	Institute for Molecular Medicine Finland (FIMM), HiLIFE,	hannele.laivuori@helsinki.fi	FinnGen Teams	Clinical Endpoint Development

For peer review only

	University of Helsinki, Helsinki, Finland			
Aki Havulinna	Institute for Molecular Medicine Finland (FIMM), HiLIFE, University of Helsinki, Helsinki, Finland; Finnish Institute for Health and Welfare (THL), Helsinki, Finland	aki.havulinna@helsinki.fi	FinnGen Teams	Clinical Endpoint Development
Susanna Lemmelä	Institute for Molecular Medicine Finland (FIMM), HiLIFE, University of Helsinki, Helsinki, Finland	susanna.lemmela@helsinki.fi	FinnGen Teams	Clinical Endpoint Development
Tuomo Kiiskinen	Institute for Molecular Medicine Finland (FIMM), HiLIFE, University of Helsinki, Helsinki, Finland	tuomo.kiiskinen@helsinki.fi	FinnGen Teams	Clinical Endpoint Development
L. Elisa Lahtela	Institute for Molecular Medicine Finland (FIMM), HiLIFE, University of Helsinki, Helsinki, Finland	laura.lahtela@helsinki.fi	FinnGen Teams	Clinical Endpoint Development
Mari Kaunisto	Institute for Molecular Medicine Finland (FIMM), HiLIFE,	mari.kaunisto@helsinki.fi	FinnGen Teams	Communication

For peer review only

	University of Helsinki, Helsinki, Finland			
Elina Kilpeläinen	Institute for Molecular Medicine Finland (FIMM), HiLIFE, University of Helsinki, Helsinki, Finland	elina.kilpelainen@helsinki.fi	FinnGen Teams	E-Science
Timo P. Sipilä	Institute for Molecular Medicine Finland (FIMM), HiLIFE, University of Helsinki, Helsinki, Finland	timo.p.sipila@helsinki.fi	FinnGen Teams	E-Science
Oluwaseun Alexander Dada	Institute for Molecular Medicine Finland (FIMM), HiLIFE, University of Helsinki, Helsinki, Finland	alexander.dada@helsinki.fi	FinnGen Teams	E-Science
Awaisa Ghazal	Institute for Molecular Medicine Finland (FIMM), HiLIFE, University of Helsinki, Helsinki, Finland	awaisa.ghazal@helsinki.fi	FinnGen Teams	E-Science
Anastasia Kytölä	Institute for Molecular Medicine Finland (FIMM), HiLIFE, University of Helsinki, Helsinki, Finland	anastasia.shcherban@helsinki.fi	FinnGen Teams	E-Science

For peer review only

Rigbe Weldatsadik	Institute for Molecular Medicine Finland (FIMM), HiLIFE, University of Helsinki, Helsinki, Finland	rigbe.weldatsadik@helsinki.fi	FinnGen Teams	E-Science
Sanni Ruotsalainen	Institute for Molecular Medicine Finland (FIMM), HiLIFE, University of Helsinki, Helsinki, Finland	sanni.ruotsalainen@helsinki.fi	FinnGen Teams	E-Science
Kati Donner	Institute for Molecular Medicine Finland (FIMM), HiLIFE, University of Helsinki, Helsinki, Finland	kati.donner@helsinki.fi	FinnGen Teams	Genotyping
Timo P. Sipilä	Institute for Molecular Medicine Finland (FIMM), HiLIFE, University of Helsinki, Helsinki, Finland	timo.p.sipila@helsinki.fi	FinnGen Teams	Genotyping
Anu Loukola	Helsinki Biobank / Helsinki University and Hospital District of Helsinki and Uusimaa, Helsinki	anu.loukola@hus.fi	FinnGen Teams	Sample Collection Coordination
Päivi Laiho	THL Biobank / Finnish Institute for Health and Welfare (THL), Helsinki, Finland	paivi.laiho@thl.fi	FinnGen Teams	Sample Logistics

For peer review only

Tuuli Sistonen	THL Biobank / Finnish Institute for Health and Welfare (THL), Helsinki, Finland	tuuli.sistonen@thl.fi	FinnGen Teams	Sample Logistics
Essi Kaiharju	THL Biobank / Finnish Institute for Health and Welfare (THL), Helsinki, Finland	essi.kaiharju@thl.fi	FinnGen Teams	Sample Logistics
Markku Laukkanen	THL Biobank / Finnish Institute for Health and Welfare (THL), Helsinki, Finland	markku.laukkanen @thl.fi	FinnGen Teams	Sample Logistics
Elina Järvensivu	THL Biobank / Finnish Institute for Health and Welfare (THL), Helsinki, Finland	elina.jarvensivu@thl .fi	FinnGen Teams	Sample Logistics
Sini Lähteenmäki	THL Biobank / Finnish Institute for Health and Welfare (THL), Helsinki, Finland	sini.lahteenmaki@th l.fi	FinnGen Teams	Sample Logistics
Lotta Männikkö	THL Biobank / Finnish Institute for Health and Welfare (THL), Helsinki, Finland	lotta.mannikko@thl. fi	FinnGen Teams	Sample Logistics
Regis Wong	THL Biobank / Finnish Institute for Health and Welfare (THL), Helsinki, Finland	regis.wong@thl.fi	FinnGen Teams	Sample Logistics

For peer review only - http://bmjopen.bmj.com/site/about/guidelines.xhtml

Auli Toivola	THL Biobank / Finnish Institute for Health and Welfare (THL), Helsinki, Finland	auli.toivola@thl.fi	FinnGen Teams	Sample Logistics
Minna Brunfeldt	THL Biobank / Finnish Institute for Health and Welfare (THL), Helsinki, Finland	minna.brunfeldt@thl .fi	FinnGen Teams	Registry Data Operations
Hannele Mattsson	THL Biobank / Finnish Institute for Health and Welfare (THL), Helsinki, Finland	hannele.mattsson@ thl.fi	FinnGen Teams	Registry Data Operations
Kati Kristiansson	THL Biobank / Finnish Institute for Health and Welfare (THL), Helsinki, Finland	kati.kristiansson@th l.fi	FinnGen Teams	Registry Data Operations
Susanna Lemmelä	Institute for Molecular Medicine Finland (FIMM), HiLIFE, University of Helsinki, Helsinki, Finland	susanna.lemmela@ helsinki.fi	FinnGen Teams	Registry Data Operations
Sami Koskelainen	THL Biobank / Finnish Institute for Health and Welfare (THL), Helsinki, Finland	sami.koskelainen@t hl.fi	FinnGen Teams	Registry Data Operations
Tero Hiekkalinna	THL Biobank / Finnish Institute for Health and Welfare (THL), Helsinki, Finland	tero.hiekkalinna@h elsinki.fi	FinnGen Teams	Registry Data Operations

For peer review only

Teemu Paajanen	THL Biobank / Finnish Institute for Health and Welfare (THL), Helsinki, Finland	teemu.paajanen@thl.fi	FinnGen Teams	Registry Data Operations
Priit Palta	Institute for Molecular Medicine Finland (FIMM), HiLIFE, University of Helsinki, Helsinki, Finland	priit.palta@helsinki.fi	FinnGen Teams	Sequencing Informatics
Kalle Pärn	Institute for Molecular Medicine Finland (FIMM), HiLIFE, University of Helsinki, Helsinki, Finland	kalle.parn@helsinki.fi	FinnGen Teams	Sequencing Informatics
Mart Kals	Institute for Molecular Medicine Finland (FIMM), HiLIFE, University of Helsinki, Helsinki, Finland	mart.kals@helsinki.fi	FinnGen Teams	Sequencing Informatics
Shuang Luo	Institute for Molecular Medicine Finland (FIMM), HiLIFE, University of Helsinki, Helsinki, Finland	shuang.luo@helsinki.fi	FinnGen Teams	Sequencing Informatics
Tarja Laitinen	The Wellbeing Services County of Pirkanmaa, Tampere, Finland	tarja.laitinen@pirha.fi	FinnGen Teams	Trajectory
Mary Pat Reeve	Institute for Molecular Medicine Finland (FIMM), HiLIFE,	mary.reeve@helsinki.fi	FinnGen Teams	Trajectory

For peer review only - http://bmjopen.bmj.com/site/about/guidelines.xhtml

	University of Helsinki, Helsinki, Finland			
Shanmukha Sampath Padmanabhuni	Institute for Molecular Medicine Finland (FIMM), HiLIFE, University of Helsinki, Helsinki, Finland	sam.padmanabhuni@helsinki.fi	FinnGen Teams	Trajectory
Marianna Niemi	University of Tampere, Tampere, Finland	marianna.niemi@tuni.fi	FinnGen Teams	Trajectory
Harri Siirtola	University of Tampere, Tampere, Finland	harri.siirtola@tuni.fi	FinnGen Teams	Trajectory
Javier Gracia-Tabuenca	University of Tampere, Tampere, Finland	javier.graciatabuenca@tuni.fi	FinnGen Teams	Trajectory
Mika Helminen	University of Tampere, Tampere, Finland	mika.helminen@tuni.fi	FinnGen Teams	Trajectory
Tiina Luukkaala	University of Tampere, Tampere, Finland	tiina.luukkaala@tuni.fi	FinnGen Teams	Trajectory
Iida Vähätalo	University of Tampere, Tampere, Finland	iida.vahatalo@epshp.fi	FinnGen Teams	Trajectory
Jyrki Pitkänen	Institute for Molecular Medicine Finland (FIMM), HiLIFE, University of Helsinki, Helsinki, Finland	jyrki.pitkanen@helsinki.fi	FinnGen Teams	Data protection officer

For peer review only

For peer review only - http://bmjopen.bmj.com/site/about/guidelines.xhtml

Marco Hautalahti	Finnish Biobank Cooperative - FINBB	marco.hautalahti@finbb.fi	FinnGen Teams	FINBB - Finnish biobank cooperative
Johanna Mäkelä	Finnish Biobank Cooperative - FINBB	johanna.makela@finbb.fi	FinnGen Teams	FINBB - Finnish biobank cooperative
Sarah Smith	Finnish Biobank Cooperative - FINBB	sarah.smith@finbb.fi	FinnGen Teams	FINBB - Finnish biobank cooperative
Tom Southerington	Finnish Biobank Cooperative - FINBB	tom.southerington@finbb.fi	FinnGen Teams	FINBB - Finnish biobank cooperative

For peer review only

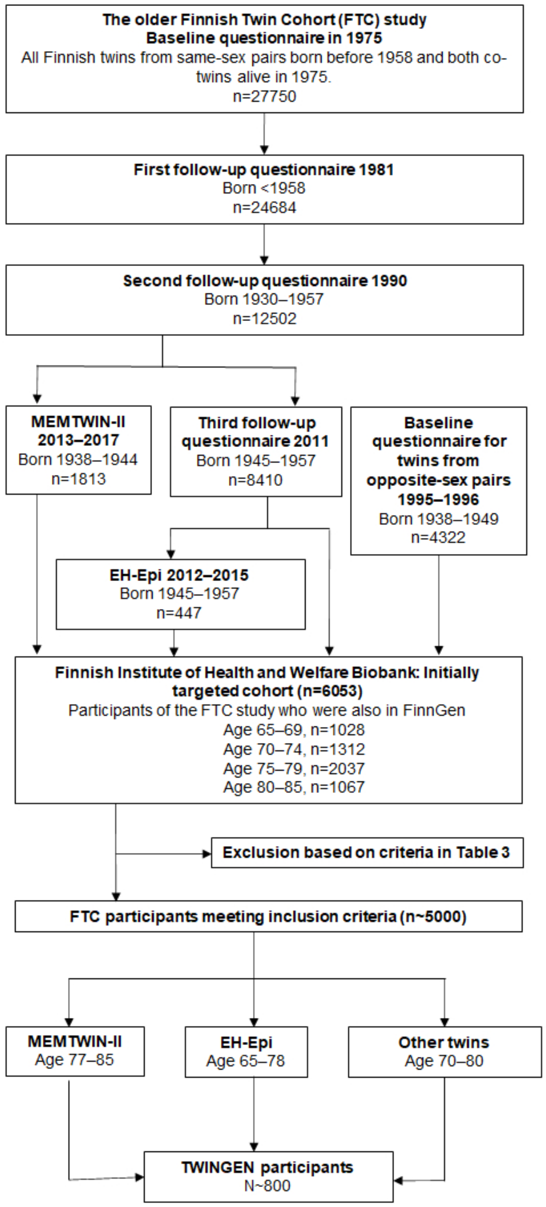

Flowchart of study participant selection

127x277mm (96 x 96 DPI)

Supplementary Figure 1. Blood samples collected in TWINGEN

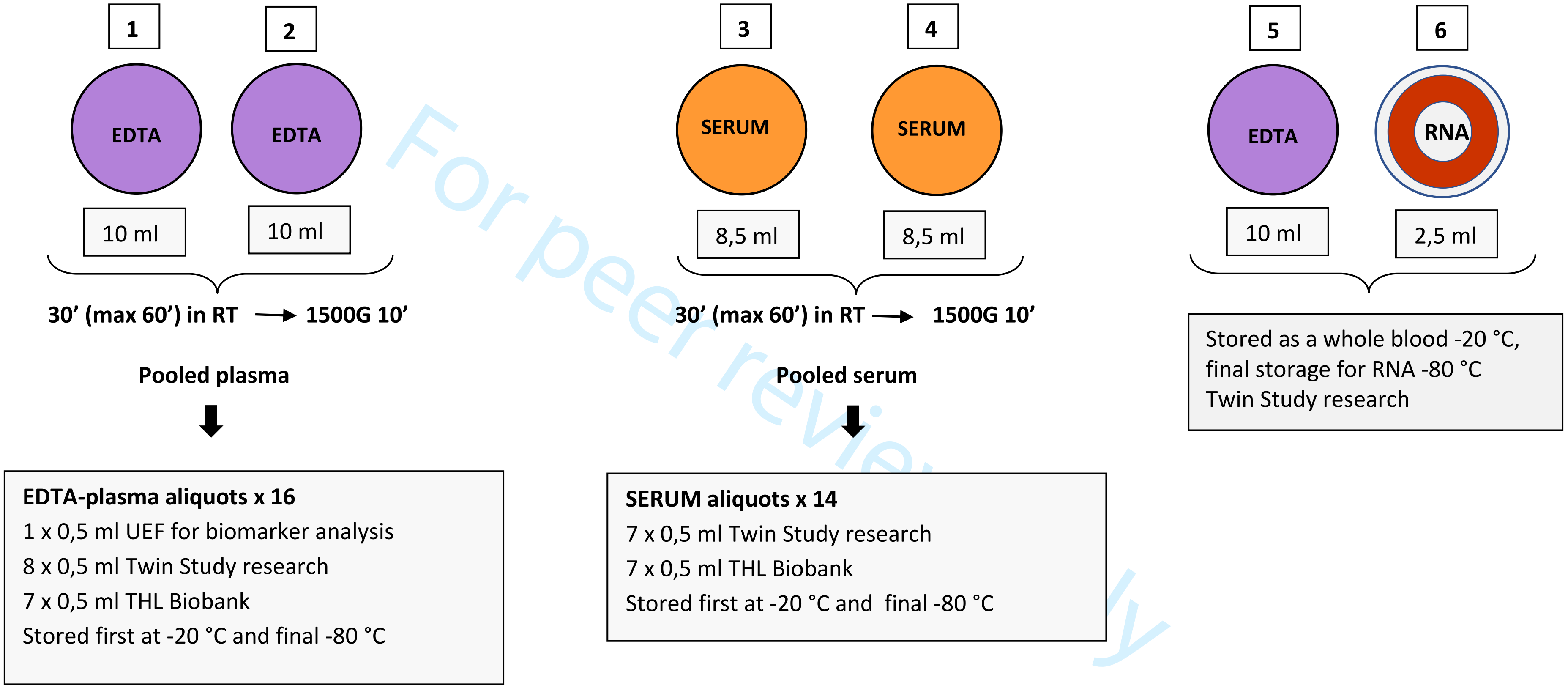

For peer review only

Supplementary Figure 2. Flow-chart of data and planned sub-studies within TWINGEN

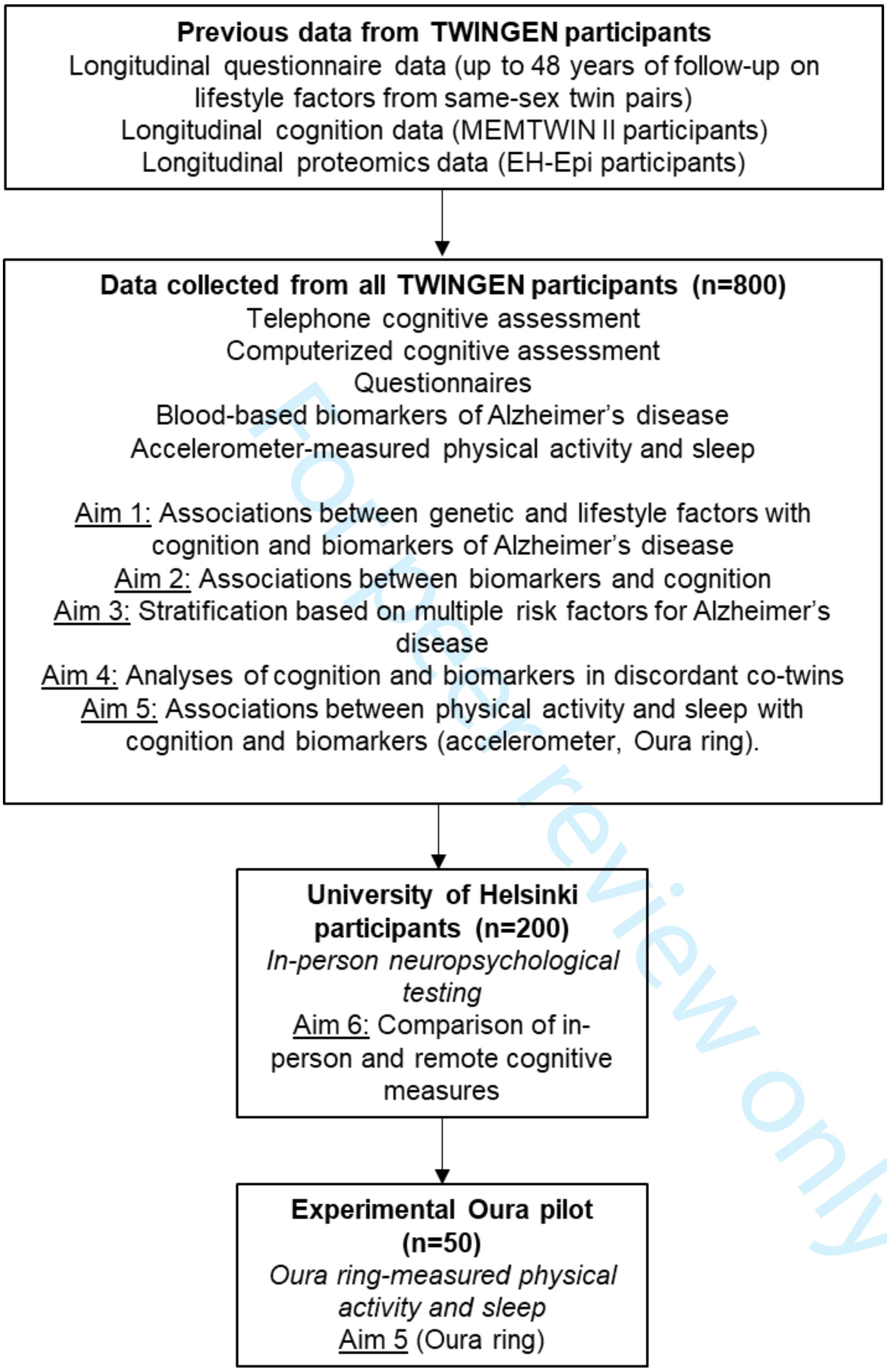

For peer review only

Supplementary Table 1. Previous data from the EH-Epi subcohort

Omics	n pairs (singletons)	Platform	Tissue	Study reference
Genotype	199 (4)	Illumina HumanCoreExome BeadChip	WBC	Drouard et al., 2022
DNA methylation	199 (4)	Illumina HumanMethylation450 BeadChip	WBC	Huang et al., 2020
Transcriptomics	195 (4)	Illumina HumanHT-12 v4 Expression BeadChip	WBC	Huang et al., 2018
miRNA	189 (3)	Illumina HiSeq	WBC	
Metabolomics	212 (10)	Nuclear magnetic resonance spectroscopy	Plasma	Drouard et al., 2022
Proteomics	210 (6)	Olink Explore	Plasma	

WBC = white blood cells

For peer review only

Supplementary Table 2. Primary and secondary references for studies on the older Finnish Twin Cohort study data

	Primary reference (PMID)	Secondary references (PMIDs)
Education	27752535	37295953, 28975875, 23344794, 15368602, 11523706
Chronic or serious illness	31462340	3499347, 7629467, 9039240, 10208188, 15935024, 19210186, 19811382, 19952836, 20516052, 20680407, 21522028, 21612334, 30626223
Smoking	3335880	7198244, 7198250, 6984028, 2661052, 10857247, 12446254, 15053857, 15203778, 17174039, 17181913, 17549066, 19246432, 19423697, 19811391, 20418888, 20418890, 23752247, 24570102, 26359785, 26407342, 27134767, 29054888, 28399944, 29730550, 30001359, 31466478, 32157176, 32361366, 32938311, 35143046, 37322846
Alcohol use	26359785	27351919, 22215005, 20858964, 16222166, 12446254, 10857250, 1590545, 3335880, 3307505, 6984028, 7198250
Sleep	31462340	37322846, 37193395, 32907578, 29047168, 25451441, 23729932, 23509990, 21731146, 20883457, 19015202, 18275555, 17969458, 16230426, 14573377, 11525419, 11403523, 8642234
Medications	29311254	12446254, 27136415, 27723547
Anthropometrics (weight, height)	26979986	10702760, 10754982, 11523706, 11665321, 12953173, 14624724, 15368602, 15971946, 16484449, 18239571, 23298777, 27964777, 31364586, 31761633, 35534559
Blood pressure	33154181	6982602, 6540964, 2858745, 3101779, 3630648, 2661052, 1418922, 10781651, 12006398, 14624727, 16268117,17129598, 19952836, 20229313, 20680407, 29311254, 32520614, 35259029
Cholesterol	33154181	2661052, 9630073, 17940543, 22286219, 23031429, 23258601, 23532744, 24082313
Diabetes	33154181	1418922, 1473616, 10768089, 10753052, 14514580, 15105411, 15935024, 19952836, 20401462, 20680407, 20706830, 23124702, 26678054, 26847990, 36638183
Life satisfaction	11092440	22980535, 22215005, 19015202, 15656900, 15583908, 14982128, 12059849, 11576076, 11229985, 11092440, 9806309, 6540964, 6540959
Loneliness	11092440	See life-satisfaction papers, loneliness is one item in the LS scale
Cognition (TELE/TICS-m)	30315123	11893836, 14512720, 14707319, 16222166, 18025784, 19587088, 20858964, 21619517, 21700581, 23532744, 24082313, 26370660, 27752535, 27589524, 30090846, 30315123, 31477183, 32843700, 32954285, 33154181, 34607247, 35134847
Depressive symptoms	Preprint: 10.31234/osf.io/jvmtz	33629475, 32361366, 31469194, 28202098, 27723547, 27161145, 27055784, 25451441, 21330272, 17181913, 15607014, 15583908, 12558538, 10893486,
Personality (EPI)	3346805	8189349, 9806309, 11576076, 12115554, 19811701, 20639285, 24570102, 24828478, 26362575, 27507146, 6540964, 3335880, 23117535, 25993607, 27089181, 33629475

For peer review only

Accelerometer (physical activity)	30445494	35770444, 32259351, 30626223, 30445494, 30090846, 29925959
Multi-omics data	35259029	Also multiple metabolomics, transcriptomics and methylation (EWAS and epigenetic ageing) analyses
DNA	31462340	Multiple GWAS consortia papers, including PMIDs: 17549066, 22241830, 23752247, 26131930, 26407342, 27089181, 27134767, 27225129, 27911795, 28892072, 30038396, 30482948, 30643251, 31294817, 32157176, 36653562

EPI = Eysenck Personality Inventory; EWAS = epigenome-wide association study; GWAS = genome-wide association study; LS = Life Satisfaction; TELE = telephone assessment for dementia; TICS-m = modified Telephone Interview for Cognitive Status

For peer review only
